# Prognosis of recurrence after complete resection in early-stage lung adenocarcinoma based on molecular alterations: a systematic review and meta-analysis

**DOI:** 10.1038/s41598-023-42851-2

**Published:** 2023-10-31

**Authors:** Chu Zhou, Zhongying Jing, Wei Liu, Zihuan Ma, Siyao Liu, Yueyu Fang

**Affiliations:** 1 Department of Thoracic Surgery, Nanjing Drum Tower Hospital, Medical School, Nanjing University, Nanjing, 210008 China; 2 Beijing ChosenMed Clinical Laboratory Co. Ltd., Beijing, 100176 China; 3 Department of Medical Oncology, Nanjing PuKou People’s Hospital, Nanjing, 211800 China

**Keywords:** Cancer, Genetics, Biomarkers

## Abstract

Molecular biomarkers have the potential to predict the recurrence risk of early-stage lung adenocarcinoma (LUAD) after complete resection, but the study results are controversial. We aimed to clarify the association of molecular alterations with disease-free survival (DFS) and recurrence-free survival (RFS) in early-stage LUAD with R0 resection. Comprehensive searches were conducted in PubMed/MEDLINE, Web of Science, and Cochrane Library for this systematic review and meta-analysis with date restrictions from 2012 to 2022. In the 18 included studies, data from a total of 7417 participants in 11 studies and 4167 participants in 9 studies were collected for the *EGFR* and *KRAS* meta-analyses, respectively. Two studies were assessed as having a moderate risk of bias, and the others were all assessed as having a high individual risk of bias. The molecular alterations in *KRAS* rather than *EGFR,* were associated with a high risk of recurrence for early-stage LUAD patients suffering from R0 resection, especially for those in pStage I, the pooled hazard ratios (HRs) of *KRAS* were 2.71 (95% CI, 1.81–4.06; *I*^2^ = 22%; *P* < 0.00001) and 1.95 (95% CI, 1.25–3.20; *I*^2^ = 57%; *P* = 0.003) with small interstudy heterogeneity in univariate and multivariate analyses, respectively. This finding suggests that molecular alterations in *KRAS* that could be detected by polymerase chain reaction techniques would provide new insight into stratifying risk and personalizing patient postoperative follow-up.

## Introduction

Complete resection is the optimal treatment for early-stage lung adenocarcinoma (LUAD), the most common subtype of lung cancer. However, recurrence occurs in 13–23% of node-negative non-small cell lung cancer patients, with a higher rate in N1 patients, after complete surgical resection^1^. Thus, recurrence hinders the hope of an absolute cure for LUAD with curative resection. Adjuvant cisplatin-based chemotherapy is recommended for early-stage LUAD patients with node-positive disease to prevent recurrence within three or five years. Its benefit for LUAD patients with stage Ib is controversial due to the low recurrence rate^2,3^. Therefore, the selection of appropriate patients is key for the application of adjuvant therapy. Currently, pathological stage (pStage) is regarded as the standard for predicting the prognosis of lung cancer patients. Notable, the emerging role of immunotherapy in early-stage LUAD argues for the use of more modern approaches to recognize patients with a high risk of postoperative recurrence^4^. Various prognostic factors for postoperative recurrence in early-stage LUAD have been reported^5^ but have failed in application.

With the development of molecular identification, some biological events are known to be related to the tumour formation process and metastasis. Clinical studies using various platforms, such as next-generation sequencing (NGS), quantitative reverse transcriptase-polymerase chain reaction (qRT-PCR), mass spectrometry, and microRNA assays, have been conducted to predict the correlation between numerous biomarkers and early recurrence after curative resection^6^. The molecular biomarkers for recurrence prognosis and the molecular heterogeneity of populations with different recurrence times remain virtually unknown. In numerous studies, *ALK* rearrangement, as well as the alterations in *RAS*, *EGFR*, *TP53*, *BRAF*, *HER2*, *PIK3CA* have been estimated as prognostic biomarkers for recurrence in early-stage LUAD patients^7,8^. Liquid biopsies is used for the monitoring the recurrence of lung cancers, which included circulating nucleic acids, circulating proteins and circulating tumour cells (CTCs)^9^. Circulating tumor DNA (ctDNA) can detect residual tumor cells persisting following surgery. A study has demonstrated that ctDNA positivity significantly correlated with increased probability of early tumor recurrence in pathologic stage I lung adenocarcinoma^10^. Moreover, common lung cancer mutations such as *TP53* detected in ctDNA can be utilized to detect cancer recurrence and assess patient response to treatment^9^. However, the results are controversial. Here, we systematically reviewed the studies concerning molecular biomarkers for prognosis in LUAD to address this confusion.

## Materials and methods

This meta-analysis was conducted following the Preferred Reporting Items for Systematic Reviews and Meta-analyses (PRISMA) and Meta-analysis of Observational Studies in Epidemiology (MOOSE) reporting guidelines^11^. The study protocol was registered in the International Prospective Register of Systematic Reviews (PROSPERO; CRD42022355090).

### Search strategy

A systematic search in PubMed/MEDLINE, Web of Science, and Cochrane Library was carried out. All studies were searched on April 20, 2022, with date restrictions (2012–2022), and all languages were included. The following terms identified as keywords were searched in the Medical Subject Headings (MeSH) term database: lung adenocarcinoma, recurrence, genomic characteristics, and mutation. The following keywords and MeSH free words were used: lung adenocarcinoma OR LUAD AND recurrence OR relapse AND mutation OR genomic characteristics. A manual search for relevant articles mentioned in reviews or articles was also performed.

### Study selection

Eligible studies had to meet the following inclusion criteria: (1) genomic characteristics were assessed; (2) the studied cohorts had undergone curative resection and were free of pStage IV lung adenocarcinoma (LUAD); and (3) disease-free survival (DFS) or relapse-free survival (RFS) was reported with hazard ratios (HRs). Studies were excluded for the following reasons: (1) the study was a case report, review article, letter, or meeting abstract; (2) patients were reported without a definite pathological stage (pStage) or with clinical stage only; (3) patients with LUAD that has metastasized; (4) HR was not reported and could not be calculated using the primary data provided in the article; (5) the study included fewer than 200 participants; and (6) papers were not written in English. Two reviewers (CZ, ZJ) independently conducted the screening procedure and assessed the study accessibility. This search procedure was performed following the Preferred Reporting Items for Systematic Reviews and Meta-Analyses (PRISMA) statement^11^.

### Data extraction

Two authors (WL, ZM) independently reviewed the acceptable articles meeting the predefined criteria mentioned above. Any discrepancies were resolved by discussion with a third reviewer (CZ). The following variables were extracted from the enrolled studies: first author and other details (year, country), sample size, pStage, histological subtype, neoadjuvant therapy, adjuvant therapy, type of study, biomarkers tested, detection platform, and outcome measured. When the data needed for meta-analysis were not available in the articles, study authors were contacted.

### Outcomes

The primary outcome of this review was to assess the association of molecular alterations with disease-free survival (DFS) or relapse-free survival (RFS) in early-stage LUAD after complete resection. Subgroup analyses were carried out to determine the clinical value of prognostic biomarkers in pStage I or I–II LUAD populations, in addition, histological classification and technical platforms were also under consideration.

### Statistical analysis

We first summarized all the molecular alterations reported in the eligible studies, including the correlation to LUAD recurrence. The subsequent meta-analysis selectively included molecular characteristics reported by three or more studies. Statistical analyses were performed by Siyao Liu and Zhongying Jing using Review Manager (RevMan), version 5. Any HRs reported or calculated from the available data were used for meta-analysis. Heterogeneity between studies was assessed using *I*^2^ statistics. Significant heterogeneity across studies was identified if *I*^2^ > 50%. Under these circumstances, the estimation of summary effect sizes would prefer a random-effects model than a fixed-effects model. The pooled effect estimates (HRs and 95% CIs) were calculated by random-effects models, accounting for potential interstudy heterogeneity. A funnel plot and Egger statistic were used to assess potential publication bias. All reported *P* values were two-tailed, and *P* < 0.05 was set as statistically significant.

### Assessment of individual study risk of bias

Two authors (CZ, ZJ) used the Quality in Prognosis Studies (QUIPS) tool to independently assess the studies as having a high, moderate, or low risk of bias^12^. We measured six domains for risk of bias: study participation, study attrition, prognostic factor measurement, outcome measurement, study confounding, and statistical analysis and reporting. A study with a low overall risk of bias was designated as having a low risk of bias in all six domains. A study was designated as having a high overall risk of bias if it had a high risk of bias in one or more domains. Disagreements were resolved by discussion or with the involvement of a panel of adjudicators (WL, ZM, SL) when necessary.

## Results

### Eligible studies

**Figure 1:**
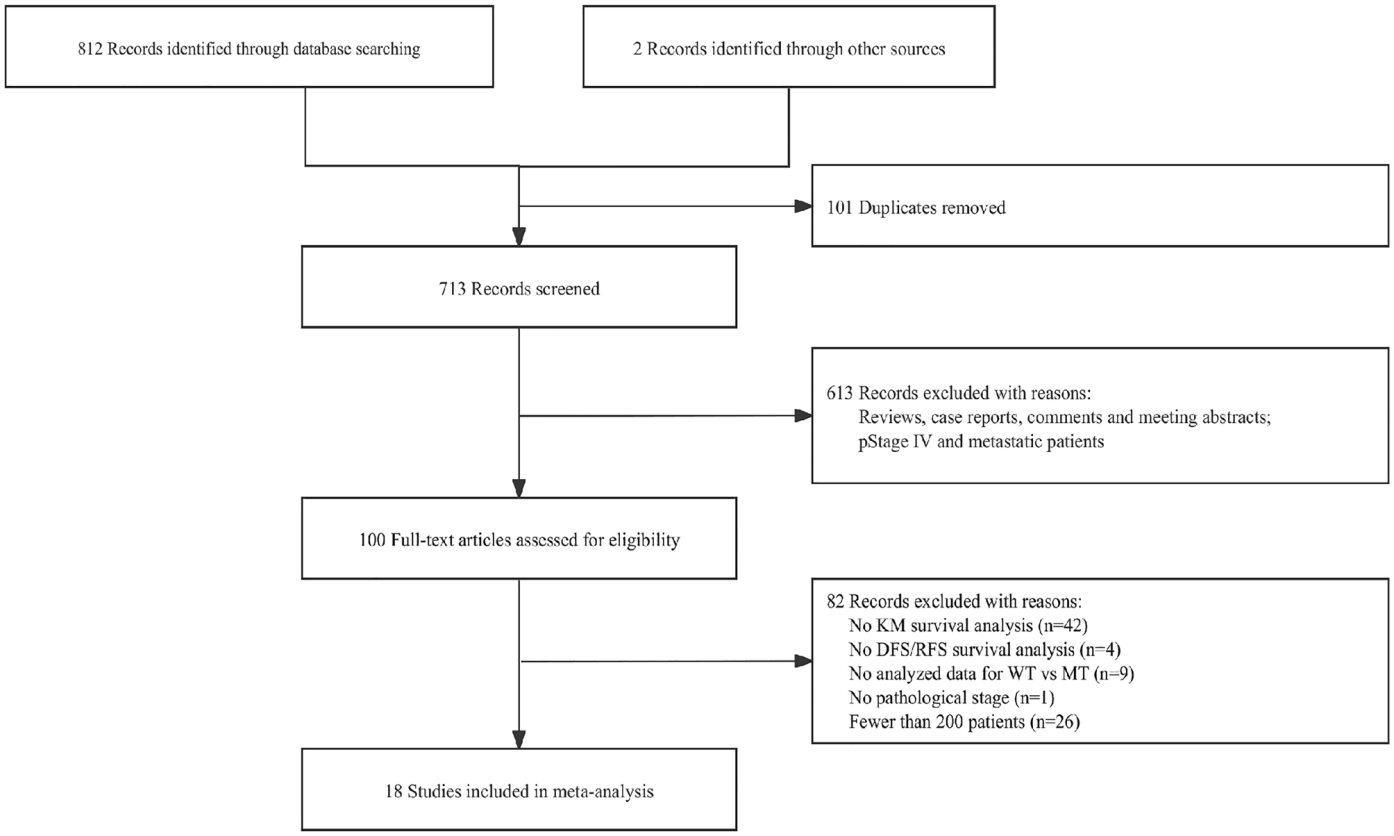
PRISMA flowchart.

A total of 713 studies without duplication were identified through the systematic search online. Following a review of the titles and abstracts, a total of 100 articles related to molecular alterations were carefully chosen by reading the entire text. The prognosis data related to *TP53*^13,14^, *BRAF*^15,16^, *HER2*^16^ and *ALK* rearrangement^17^ were excluded due to a limited number of studies. Finally, 18 studies were included in the final meta-analyses, which studied *EGFR* in 11 articles, and *KRAS* in 9 articles. Figure 1 shows the process of study selection.

Overall, the clinical studies varied in the methodology for detecting molecular alterations as shown in Table 1. Seven studies used the polymerase chain reaction (PCR) to examine *EGFR*^18–21^ or *KRAS* alterations^22–24^ as a marker of disease-free survival; while five studies used the next-generation sequencing (NGS) to detect *EGFR*^13,14,16^ and *KRAS*^14,16,25^ alterations. Multiple detection methods were adopted to verify the molecular alterations by six teams of researchers^15,26–29^. Sanger sequencing and matrix-assisted laser desorption/ionization time-of-flight mass spectrometry (MALDI–TOF–MS) were employed by Kadota et al. and Deng et al., respectively^30,31^.

**Table 1:** Detection methods of molecular biomarkers.

Study	Biomarker	Mutation rate	Method of biomarkers analysis	Detection range of *EGFR/KRAS*	Limit of detection
Aokage et al., 2021^18^	*EGFR*	39.5%	PCR	Mutations in exons 18, 19, 20, and 21	1% mutation
Deng et al., 2021^30^	*EGFR*	61.80%	Sanger sequencing	EGFR (exons 18–22)	NA
Hayasaka et al., 2018^26^	*EGFR*	48.98%	Multiple platform: PCR; direct sequencing method, PNA-LNA PCR	NA	NA
Isaka et al., 2018^27^	*EGFR*	45.60%	Multiple platform: PCR; LH-MSA	EGFR (exon 18–21)	NA
Ito et al., 2020^19^	*EGFR*	49.40%	PNA-LNA PCR	NA	0.1% mutation
Ito et al., 2018^20^	*EGFR*	44.70%	PCR	EGFR point mutations in Ex18 (G719X) or Ex21 (L858R, L861Q), and deletion in Ex19	NA
Kim et al., 2021^14^	*EGFR*/*KRAS*	EGFR: 52.2%; KRAS: 14.3%	NGS	NA	Depth of 1000 ×
Kondo et al., 2022^21^	*EGFR*	52.7%	PCR	Exon 18(G719X), exon 20 (S768I) and exon 21 (L858R, L861Q), insertion in exon 20 and deletion in exon 19	NA
Matsumura et al., 2017^29^	*EGFR*	49%	Multiple platform: PCR; direct sequencing method	NA	NA
Shimizu et al., 2017^16^	*EGFR*/*KRAS*	EGFR: 46.9%; KRAS: 14.9%	NGS	NA	NA
Zhou et al., 2021^13^	*EGFR*	100%	NGS	NA	1000 ×
Izar et al., 2014^28^	*KRAS*	41%	Multiple platform: PCR; FISH	NA	PCR: sensitivity of 95%
Jones et al., 2021^25^	*KRAS*	32%	NGS	NA	NA
Kadota et al., 2016^31^	*KRAS*	27%	MALDI–TOF–MS	NA	NA
Kneuertz et al., 2020^15^	*KRAS*	38.1%	Multiple platform: Sanger sequencing; FISH; NGS	KRAS exon 2 mutation (codon 12 and 13)	NA
Li et al., 2018^22^	*KRAS*	17.4%	PCR	KRAS (exons 2–3)	NA
Ma et al., 2022^23^	*KRAS*	29.2%	PCR	NA	NA
Ohba et al., 2016^24^	*KRAS*	5.5%	PCR	KRAS (codon 12–13)	NA

*PCR* polymerase chain reaction, *NGS* next-generation sequencing, *IHC* immunohistochemistry, *FISH* fluorescence in situ hybridization, *LH-MSA* loop-hybrid mobility shift assay, *MALDI–TOF–MS* matrix-assisted laser desorption/ionization-time of flight/mass spectrometry.

### Patient characteristics

A total of 11,314 LUAD patients were investigated (7417 and 4167 patients involved in *EGFR* and *KRAS* analysis, respectively). The patients’ characteristics are summarized in Table 2. Among five studies, patients with adenocarcinoma in situ (AIS) were included in the survival analysis^18,26,29–31^. Of note, the 8^th^ edition of the International Association for the Study of Lung Cancer (IASLC) tumour node metastasis (TNM) classification system clearly states that AIS is stage 0^32^. Four studies with incorrect pStage information were revised under this guideline^26,29–31^. Moreover, the histology of the patients could not be acquired from several studies^14–16,24,27,28^. The enrolled patients in six studies were restricted to those with invasive LUAD for the final survival analysis^13,19–21,23,25^, and patients with lepidic adenocarcinoma (LPA) and pure ground-glass opacity (GGO) adenocarcinoma were excluded from the study by Ma et al.^23^. As shown in Table 2, approximately half of the studies (8/18) mainly analyzed the pStage I–III LUAD patients with complete resection, and four studies focused on pStage I LUAD patients. There were obvious distinctions in the integrity of therapy information among the studies. No neoadjuvant and adjuvant therapy information was provided in 6^18–20,24,26,31^ and 12 studies^14–16,19,20,22–25,27,30,31^, respectively. In a total of 11/18 studies, the enrolled patients received no neoadjuvant therapy^13–16,21–23,25,27,28,30^, and 1033 patients in two studies did not receive adjuvant treatment^21,28^. These confounding factors among the studies partially contribute to the individual risk of bias and publication bias.

**Table 2:** Study characteristics.

Study	Country	Biomarker	Sample size	Histological subtype	Pathological stage	Neoadjuvant therapy	Adjuvant chemotherapy	Design	Outcome
Aokage et al., 2021^18^	Japan	*EGFR*	489	All	0, IA–IIIB	NS	No: 192; Yes: 104	Retrospective	RFS
Deng et al., 2021^30^	China	*EGFR*	1512	All	**0, I–III**	NO	NS	Retrospective	RFS
Hayasaka et al., 2018^26^	Japan	*EGFR*	835	All	**0, IA–III**	NS	Yes	Retrospective	RFS
Isaka et al., 2018^27^	Japan	*EGFR*	237	NS	I–III	NO	NS	Retrospective	RFS
Ito et al., 2020^19^	Japan	*EGFR*	1155	Invasive LUAD	IA–IIIA	NS	NS	Retrospective	RFS
Ito et al., 2018^20^	Japan	*EGFR*	394	Invasive LUAD	IA–IIA	NS	NS	Retrospective	RFS
Kim et al., 2021^14^	Korea	*EGFR*/*KRAS*	230	NS	IA–IIB	NO	NS	Retrospective	RFI
Kondo et al., 2022^21^	Japan	*EGFR*	721	Invasive LUAD	I	NO	NO	Retrospective	RFS
Matsumura et al., 2017^29^	Japan	*EGFR*	909	All	**0, IA–IIIB**	No EGFR-TKI	No EGFR-TKI	Retrospective	RFS
Shimizu et al., 2017^16^	Japan	*EGFR*/*KRAS*	303	NS	I–III	NO	NS	Retrospective	RFS
Zhou et al., 2021^13^	China	*EGFR*	637	Invasive LUAD	IA–IIIA	NO	No: 575; Yes: 62	Retrospective	DFP
Izar et al., 2014^28^	America	*KRAS*	312	NS	I	NO	NO	Retrospective	DFS
Jones et al., 2021^25^	America	*KRAS*	476	Invasive LUAD	I–III	NO	NS	Prospective	DFS
Kadota et al., 2016^31^	America	*KRAS*	463	All	**0, IA–IB**	NS	NS	Retrospective	CIR
Kneuertz et al., 2020^15^	America	*KRAS*	324	NS	IA–IIIB	NO	NS	Retrospective	DFS
Li et al., 2018^22^	China	*KRAS*	1098	Invasive and minimally invasive LUAD	I–III	NO	NS	Retrospective	DFS
Ma et al., 2022^23^	China	*KRAS*	1181	Invasive LUAD (except LPA and GGO)	I–III	NO	NS	Prospective	RFS
Ohba et al., 2016^24^	Japan	*KRAS*	256	NS	I	NS	NS	Retrospective	DFS

The pathological stage writing in bold letters was revised following the 8th edition of the International Association for the Study of Lung Cancer (IASLC) tumor node metastasis (TNM) classification system.

*LUAD* lung adenocarcinoma, *LPA* lepidic predominant adenocarcinoma, *GGO* ground-glass opacity, *CIR* cumulative incidence of recurrence, *DFP* disease-free proportion, *DFS* disease-free survival, *RFI* recurrence-free interval, *RFS* relapse-free survival, *TKI* tyrosine kinase inhibitors, *NS* not sure.

### Individual study risk of bias

As shown in Supplementary Fig. S1, there were no studies with an overall low risk of bias, while two studies and seventeen studies had an overall moderate risk of bias and high risk of bias, respectively. Detailed information on the individual risk of bias is presented in Supplementary Table S1.

### Outcomes

A meta-analysis of prognostic outcomes (time to event) was statistically analysed using hazard ratios (HRs). Study outcomes were extracted while trying to keep definitions consistent, and endpoint data were clarified from the definition. Almost all the included studies (17/18) clearly defined the study endpoint, and the subtle differences among the studies are shown in Supplementary Table S2. The prognostic outcomes included recurrence-free survival (RFS), disease-free survival (DFS), cumulative incidence of recurrence (CIR), disease-free proportion (DFP), and recurrence-free interval (RFI). The majority of the prognostic outcomes in the studies of postoperative recurrence risk in LUAD patients with *EGFR* alterations and *KRAS* alterations were RFS and DFS, respectively. Aokage et al. and Hayasaka et al. defined RFS as the interval from the date of surgery to the date of first recurrence, date of death due to any cause, or date of last follow-up^18,26^. In 10 studies, the prognostic outcomes were set as the interval from the date of surgery to the date of first recurrence^16,19,20,24,27^ or extended to the last follow-up^13,14,23,29,30^. The endpoint of the others studies was the first recurrence or death from any cause^15,21,25,28^. All the various research endpoints were considered to represent the prognostic outcomes in the survival analysis. We did not aim to compare the impact of various outcome indices on the prognostic results owing to the known heterogeneity in study design.

### *EGFR* mutations and the recurrence risk of LUAD

**Figure 2:**
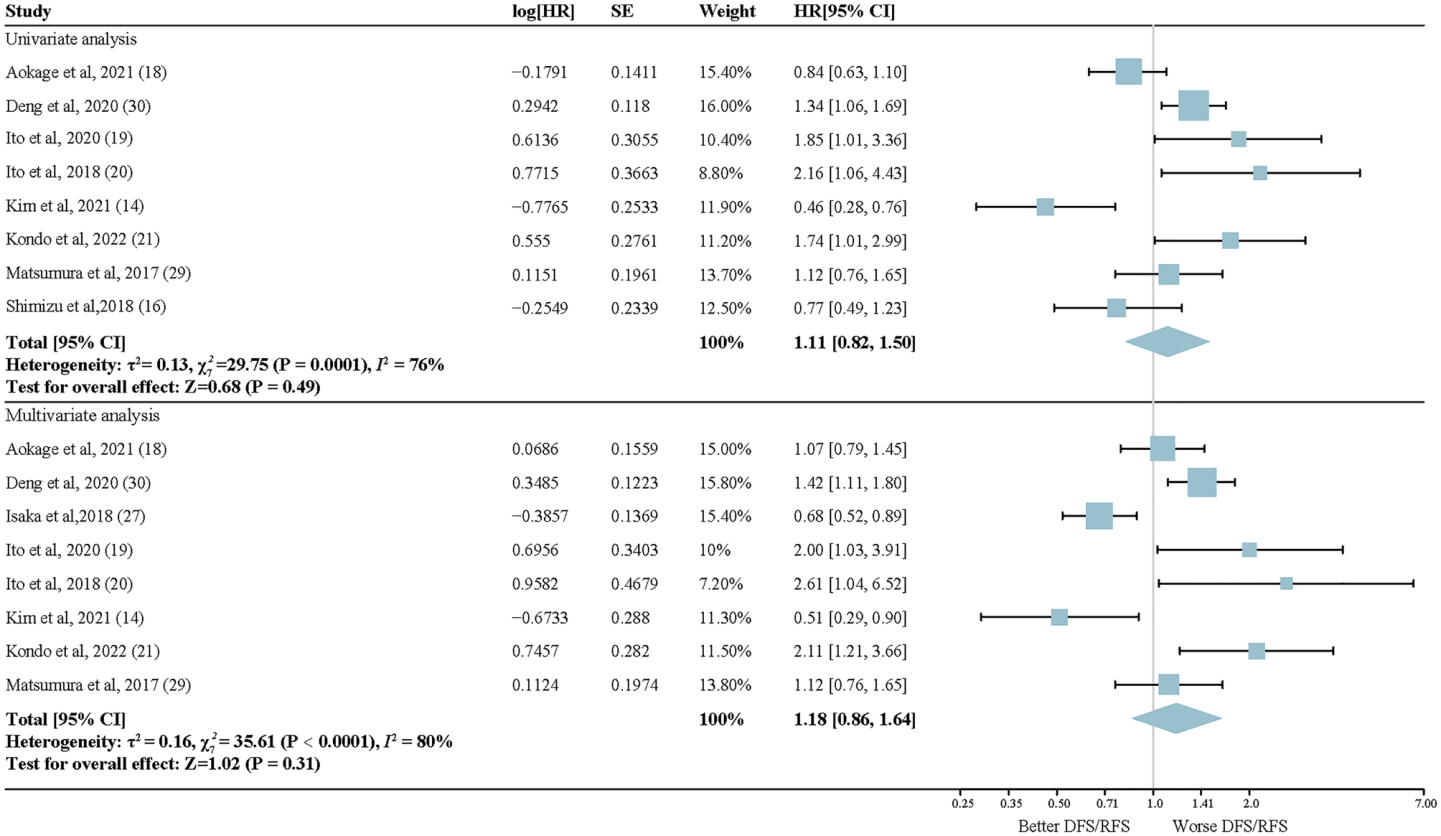
Forest plots of random-effects model for the association between *EGFR* mutation status and reduced DFS/RFS in lung adenocarcinoma. Upper section: Univariate analysis; Lower section: Multivariate analysis. HR, hard ratio.

**Figure 3:**
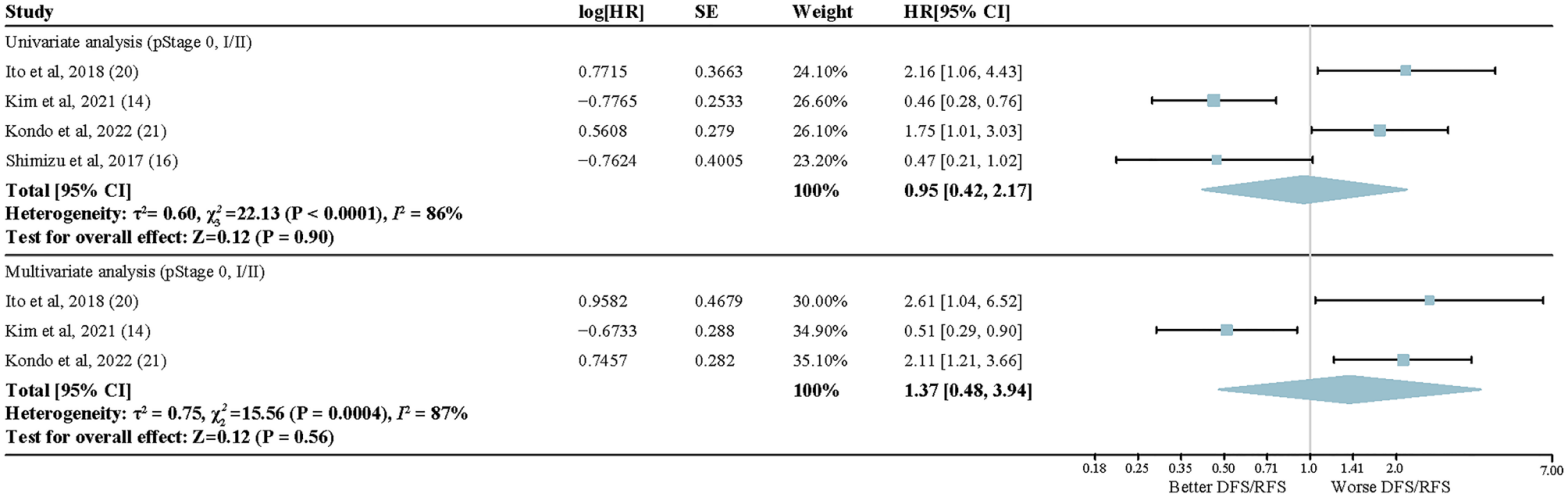
Forest plot describing subgroup analysis of random-effects model for the association between *EGFR* mutation status and DFS/RFS in early-stage lung adenocarcinoma (pStage 0, I/II). Upper section: Univariate analysis; Lower section: Multivariate analysis.

**Figure 4:**
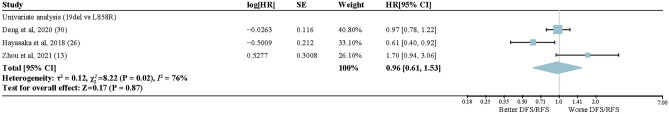
Forest plots of random-effects model for the association between DFS/RFS with 19 Del vs L858R mutation in early-stage lung adenocarcinoma (pStage 0, I/II).

A total of 11 articles with 7417 patients were included in the meta-analysis for postoperative recurrence risk in early-stage LUAD with *EGFR* alterations. There was no statistically significant difference in recurrence risk between the early-stage LUAD patients with *EGFR* alterations and wild-type patients in either univariate (HR, 1.11; 95% CI, 0.82–1.50; *P* = 0.49) or multivariate analysis (HR, 1.18; 95% CI, 0.86–1.64; *P* = 0.31). Of note, statistically significant heterogeneity existed between these articles, as shown in Fig. 2 (univariate: *I*^2^ = 76%; multivariate: *I*^2^ = 80%). Subgroup meta-analysis was performed on pStage 0 and I–II LUAD patients who underwent surgery, as shown in Fig. 3. *EGFR* mutation was not proven to be a prognostic biomarker for postoperative recurrence of LUAD patients with pStage 0 and I–II in either univariate or multivariate analysis, and considerable heterogeneity between these studies could not be ignored (univariate: HR, 0.95; 95% CI, 0.42–2.17; *I*^2^ = 86%; *P* = 0.90; multivariate: HR, 1.37; 95% CI, 0.48–3.94; *I*^2^ = 87%; *P* = 0.56;). In the subgroup analysis using PCR as the detection method, our results revealed a significant association between alterations in *EGFR* and a shorter postoperative DFS/RFS in early-stage LUAD patients when considering multivariate analysis (HR, 1.69; 95% CI, 1.07–2.68; *P* = 0.02). However, in the univariate analysis, no significant association was found between *EGFR* mutation status and DFS/RFS (HR, 1.47; 95% CI, 0.87–2.48; *P* = 0.15) (Supplementary Fig. S2). The results in Supplementary Figs. S3–S6 showed that there was little evidence of reporting bias according to visual examination of the funnel plot, but the Egger tests were not significant. With regard to the alteration sites, exon 19 deletions had no significantly different impact on recurrence compared to the L858R mutation in exon 21 (HR, 0.96; 95% CI, 0.61–1.53; *I*^2^ = 76%; *P* = 0.87) (Fig. 4), and no reporting bias for small-study effects was identified (Supplementary Fig. S7).

Thus, the early-stage LUAD patients with *EGFR* mutation have no difference in relapse risk as against the wild-type patients after complete resection. The various mutation sites in *EGFR*, such as L858R or exon 19 deletions, did not affect the above results. In our subgroup analysis using PCR as the standardized detection method, the multivariate analysis revealed a significant association between *EGFR* mutations and the DFS/RFS of early-stage lung cancer patients.

### *KRAS* mutations and the recurrence risk of LUAD

**Figure 5:**
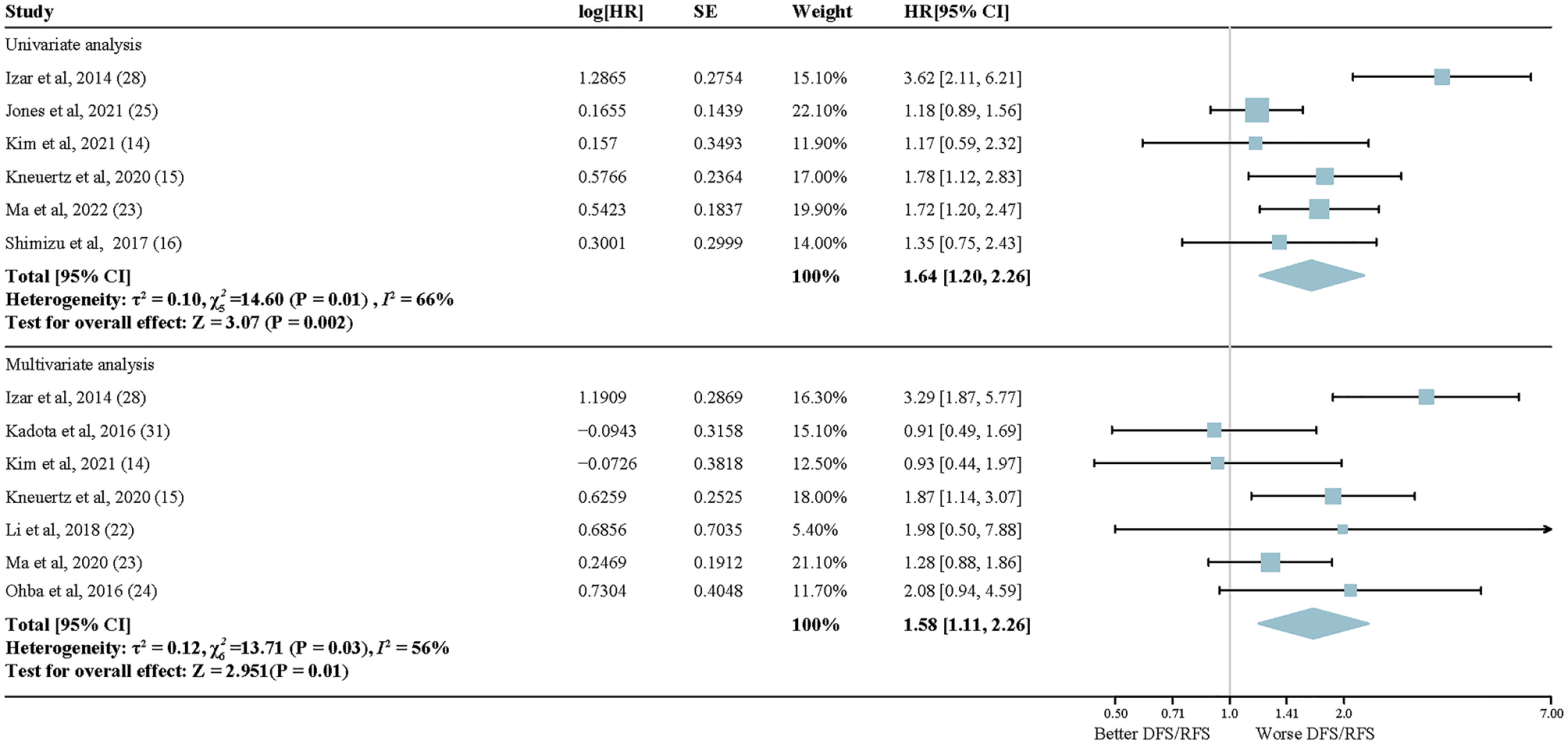
Forest plots of random-effects model for the association between *KRAS* mutation status and reduced DFS/RFS in lung adenocarcinoma. Upper section: Univariate analysis; Lower section: Multivariate analysis.

**Figure 6:**
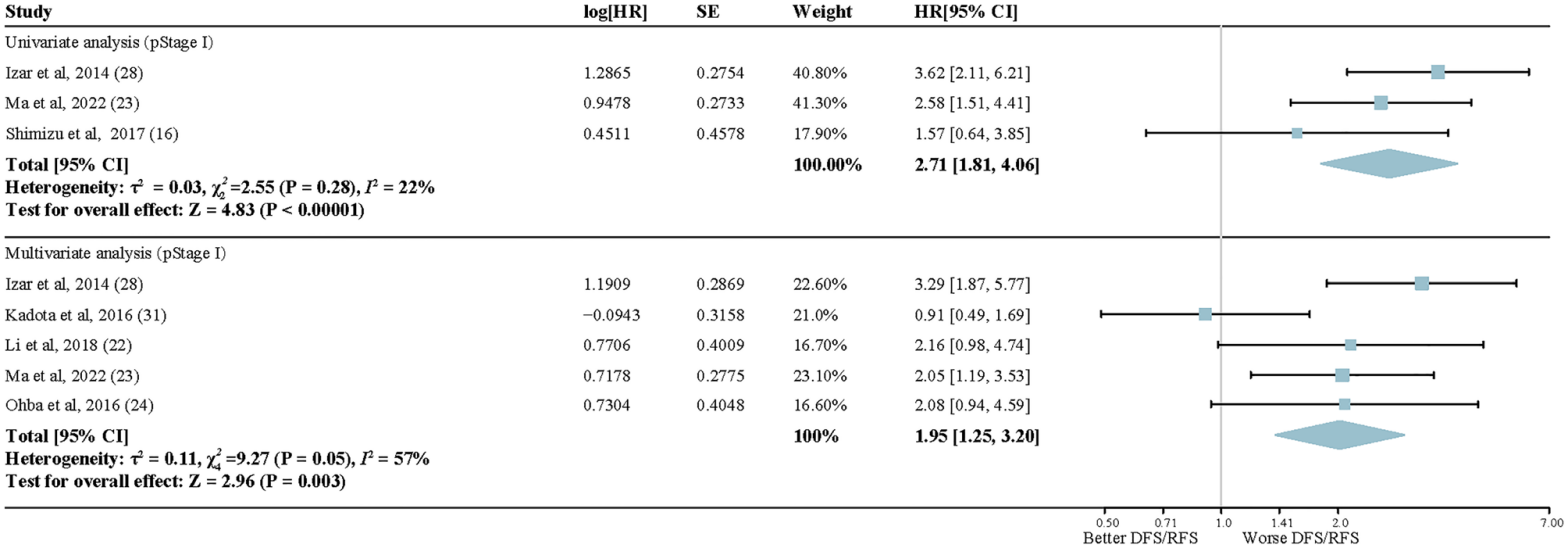
Forest plot describing subgroup analysis of random-effects model for the association between subgroup analysis of *KRAS* mutation status and DFS/RFS in early-stage lung adenocarcinoma (pStage I). Upper section: Univariate analysis; Lower section: Multivariate analysis.

Nine studies of 4167 early-stage LUAD patients were included in the prognosis analysis for *KRAS* mutations. As shown in Fig. 5, molecular alterations in *KRAS* were significantly associated with a shorter postoperative DFS/RFS of early-stage LUAD patients (univariate: HR, 1.64; 95% CI, 1.20–2.26; *P* = 0.002; multivariate: HR, 1.58; 95% CI, 1.11–2.26; *P* = 0.01). Significant heterogeneity existed in the studies irrespective of univariate or multivariate analysis (*I*^2^ = 66%, *I*^2^ = 56%, respectively). Subgroup analysis showed that the heterogeneity in the studies on Americans was much higher than that in the studies on Asians, whether in univariate analysis (Asian: *I*^2^ = 0%; American: *I*^2^ = 85%) or in multivariate analysis (Asian: *I*^2^ = 0%; American: *I*^2^ = 78%) (Supplementary Fig. S8). In pStage I LUAD patients with lobectomy, there was a statistically significant higher recurrence risk for patients with *KRAS* alterations. HRs were 2.71 (95% CI, 1.81–4.06; *P* < 0.00001) and 1.95 (95% CI, 1.25–3.2; *P* = 0.003) in the univariate and multivariate analysis, respectively, with little interstudy heterogeneity (univariate: *I*^2^ = 22%; multivariate: *I*^2^ = 57%) (Fig. 6). In the multivariate analysis using PCR as the detection method, *KRAS* mutation was significant association with a shorter postoperative DFS/RFS in early-stage LUAD patients (HR, 1.43; 95% CI, 1.03–1.98; *P* = 0.03). Conversely, when considering the univariate analysis utilizing NGS as the detection method, no significant association was found between *KRAS* mutation status and DFS/RFS (HR, 1.21; 95% CI, 0.95–1.53; *P* = 0.12) (Supplementary Fig. S9). Reporting bias were absent according to Egger statistic, and few small-study effects were visualized from the funnel plots (Supplementary Figs. S10–S13).

## Discussion

In this systematic review and meta-analysis, molecular biomarkers in early-stage LUAD patients who underwent surgery were examined. For surgical patients, particularly those in pStage I, the meta-analysis indicated that *KRAS* mutation was associated with a greater risk of recurrence, whereas *EGFR* was not a predictive biomarker for recurrence and other molecular biomarkers were not sufficiently explored. According to our evaluation, the included studies had a moderate to high risk of bias because of shoddy study design, crude conduction, poorly chosen study confounders, or biased reporting and analysis. The fact that these methodological flaws are typical in molecular biology research on prognostic biomarkers emphasizes an urgent need for change.

In more recent years, sophisticated techniques based on polymerase chain reaction (PCR) have been adopted to detect molecular alterations in various cancers. Seven of the studies included in the current analyses used PCR to measure gene variants^18–24^, and one used Sanger sequencing technology^30^. One of the prominent limitations of PCR or Sanger sequencing technology is that information on the molecular variation being studied is required at the beginning of the experiment. Next-generation sequencing (NGS) employed in four studies^13,14,16,25^, addresses this limitation and allows for the detection of multiple variations. However, the false-positive and false-negative variants reported by one platform cannot be ignored. Only 5/18 studies verified the test results by fluorescence in situ hybridization (FISH), immunohistochemistry (IHC) or other platforms^15,26–29^. In addition to the diversity of detection technology among the studies, patient characteristics, therapy, and clinical information were not comprehensive due to the retrospective nature of the study. Moreover, we found an incorrectly defined clinical endpoint in some studies. Relapse-free survival (RFS) was regarded as disease-free survival (DFS)^18,26^ or vice versa^16,24^. The studies included in the meta-analysis were primarily conducted in America, Japan, China and Korea, studies from other countries could be excluded owing to lack of valid data available. These various confounding factors in the included studies contributed to the individual risk of bias and reporting bias according to visual observation of funnel plots.

Our continued focus, along with that of other specialists, has been on early recurrence after surgery. A unique prognostic molecular biomarker, detected by a convenient and economic platform such as PCR, will be ideal for clinical practice. The suppressor genes *APC* and *BRCA* detected by clinicians provide a strong indication for evolution into colorectal cancer and breast cancer, respectively^33,34^. In 2016, the team at the University of Oxford discovered that alterations in *POLE* proofreading are ideal biomarkers for recurrence in early-stage colorectal cancer, and these patients have the potential to avoid adjuvant chemotherapy^35^. All these important breakthroughs in molecular biomarkers for cancer screening or recurrence are substantial benefits in reducing mortality and improving overall survival. In LUAD patients who have undergone lobar or sublobar resection? To date, no definite molecular biomarkers of relapse have been applied in clinical practice. Perioperative dynamic changes in circulating tumour DNA (ctDNA) have been shown to be a baseline value for postoperative recurrence^36,37^. The detection of ctDNA in several types of liquid biopsy is currently hindered by economic and time constraints, and a unique preoperative or postoperative time point for liquid biopsies is not fixed in clinical studies. Of note, the panel used for ctDNA level detection consistently includes hotspot mutations, but the partial population with novel single-nucleotide variants will be missed^38^. During the course of this systematic review and meta-analysis, we did not observe any studies continuously focusing on the exploration of molecular biomarkers of relapse and their application in clinical practice. It was also interesting to note that the effect of *EGFR* mutations on the risk of recurrence is controversial in different studies^19,29^. The results of our review addressed this dispute.

Although *EGFR* functions in tumor processing and is a common therapeutic target, it was not proven to be a prognostic biomarker for recurrence in early-stage LUAD with R0 resection in our meta-analysis. To clarify the cause of disparate results in the studies, we thoroughly scrutinized the detailed information, which included the pathological characteristics (such as pStage and histology subtypes), molecular detection platform, and definition of outcome, between two study groups with higher and lower HRs for DFS/RFS. Interestingly, in two separate analyses focusing on high-grade patterns (solid or micropapillary component), *EGFR* mutations were significantly associated with poorer RFS in univariate and multivariate analysis^21,30^. Moreover, when considering the unified detection method of PCR, multivariate analysis revealed *EGFR* mutation was association with worse prognosis. However, it is important to note that these findings should be further investigated with a larger number of studies and patients due to the limited inclusion of literatures. In addition, exon 19 deletions and the L858R mutation account for 90% of all *EGFR* mutations. A large cohort study, which included lung adenocarcinoma patients staged I–IV, demonstrated that compared to patients with the L858R mutation, patients with exon 19 deletions exhibited poor RFS^39^. However, in our study focused on early-stage lung adenocarcinoma, no DFS/RFS difference was observed between patients with alterations in L858R and patients with exon19 deletions. This discrepancy may be attributed to the limited sample size and inclusion of only early-stage lung adenocarcinoma patients in our study. Furthermore, in cases of lung adenocarcinoma, *EGFR* mutation has been associated with various prognostic factors^40^, including gender, smoking status, tumour size^18,20,39,41^, and ground-glass opacities (GGOs) within lung nodules^18,41^. These additional factors may have contributed to the interstudy heterogeneity observed in the meta-analysis. However, due to the limitation of available data in the included studies, we were unable to conduct more detailed subgroup analyses. We also browsed the PubMed library for articles typed in meta-analysis to clarify whether *EGFR* mutation serves as a prognostic biomarker for recurrence in other cancers, but no results were obtained. Here, we confidently assert that *EGFR* alterations did not result in shorter or longer DFS/RFS for early-stage LUAD treated with surgery. Perhaps this is also true for other cancers, and the negative results were not often published.

In our review, *KRAS* mutation, which commonly occurs in codon 12*,* was proven to be a predictive biomarker of postoperative relapse in LUAD patients. Patients with *KRAS* alterations in the early stage have shorter DFS/RFS. Particularly for patients in pStage I, the interstudy heterogeneity was less than 50%. Of the 10 studies in the *KRAS* meta-analysis, one study performed by Ma et al.^23^ was assessed as having a moderate risk of bias, and the others had a high individual risk of bias. The studies involving Americans may be one of the sources of interstudy heterogeneity, as the *I*^2^ was much higher than that in studies from Asians. For the patients in pStage I–III, the relatively high individual risk of bias in studies may largely be attributed to the significant interstudy heterogeneity. In the subgroup analysis using PCR as the detection method, *KRAS* mutations were associated with poorer RFS/DFS in multivariate analysis. However, the subgroup analyses for patients with pStage II, pStage III, or particular histological subtypes were unable to perform due to narrow clinical studies. In one study analyzing the risk of postoperative recurrence with *KRAS* alterations based on histological classification, it was found that *KRAS* mutation was associated with poor RFS in solid predominant tumors, but not in non-solid predominant tumors^31^. The most common amino acid specific mutations are G12C and G12V. Jones et al.’s study have demonstrated that compared to other sites of *KRAS* mutation, *KRAS* G12C mutation was independently associated with worse DFS in the multivariable analysis^25^. Additionally, in a recent study, it was indicated that *KRAS* G12C mutation, rather than non-G12C mutation, served as an independent predictor for early recurrence in early-stage lung adenocarcinoma^42^. This suggests that *KRAS* G12C mutation plays a crucial role in the process of recurrence and further research with a larger number of studies is warranted for a deeper understanding. Moreover, two meta-analyses confirmed that *KRAS* mutation was also a predictor for poor DFS and OS in early-stage colorectal cancer^43,44^. Concerning the function of *KRAS*, we hypothesize that its activation drives cells of minimal residual disease (MRD) into rapid proliferation and thus induces early recurrence and disease deterioration.

In this systematic review, we conducted a thorough assessment of prognostic studies involving molecules in LUAD. For the results to be accurate, cohorts with fewer than 200 patients were excluded. There are still a few limitations, however. Despite using a random-effects model, the main limitation is the interstudy heterogeneity that is reflected in the wide CIs. Moreover, the meta-analysis could not include all of the relevant literature pertaining to this subject due to data unavailability. The studies including in our meta-analysis came from the American, Japan, China, and Korea, which may have introduce selection bias and limited the generalizability of our findings beyond Asia and the American. Among the analysed studies, there were subtle discrepancies in the definition of assessment indices and histological subtypes. Another limitation is, the variety of molecular detection platforms. All common mutation sites discovered by PCR or Sanger sequencing would be covered by the NGS platform. To lessen the bias of the results, future studies should, wherever possible, take essential mutation sites into consideration. More importantly, the prognosis of molecular biomarkers in different histological subtypes should be meta-analysed.

## Conclusion

This meta-analysis showed that *KRAS* mutation, but not *EGFR* mutation was a molecular biomarker for shorter DFS or RFS in early-stage LUAD patients after complete resection. However, the interstudy heterogeneity cannot be ignored and was less than 50% for patients in pStage I. *KRAS* mutation has the potential to identify postoperative recurrence in LUAD populations when used as a clinical biomarker. In an era of personalized surveillance, *KRAS* alterations present an opportunity for guiding future precision medicine.

### Supplementary Information

Supplementary Information 1.

Supplementary Information 2.

## Data Availability

The information of the search strategy and literature list are provided in the Additional files, as well as the detail information of articles used in our meta-analysis. The information authors declare that all data used or analyzed during the current study are available on reasonable request. Researchers can contact the corresponding author by email.
