# Supplementary material for: Prognosis of recurrence after complete resection in early-stage lung adenocarcinoma based on molecular alterations: a systematic review and meta-analysis
Source: Sci Rep. 2023 Oct 31;13:18710. doi: 10.1038/s41598-023-42851-2 (PMC10618289; doi:10.1038/s41598-023-42851-2)
Supplement: Supplementary file 1 — Supplementary Information 1. [file 41598_2023_42851_MOESM1_ESM.zip › Additional Files/Cochrane Library-lung adenocarcinoma OR LUAD AND recurrence OR relapse AND mutation OR genomic characteristics.rtf]

Badeau, M., Lindsay, C., Blais, J., Nshimyumukiza, L., Takwoingi, Y., Langlois, S., . . . et al. (2017). Genomics‐based non‐invasive prenatal testing for detection of fetal chromosomal aneuploidy in pregnant women. Cochrane Database of Systematic Reviews(11). doi:10.1002/14651858.CD011767.pub2
Cameron, L. B., Hitchen, N., Chandran, E., Morris, T., Manser, R., Solomon, B. J., & Jordan, V. (2022). Targeted therapy for advanced anaplastic lymphoma kinase (ALK)‐rearranged non‐small cell lung cancer. Cochrane Database of Systematic Reviews(1). doi:10.1002/14651858.CD013453.pub2
Cornelisse, S., Zagers, M., Kostova, E., Fleischer, K., van Wely, M., & Mastenbroek, S. (2020). Preimplantation genetic testing for aneuploidies (abnormal number of chromosomes) in in vitro fertilisation. Cochrane Database of Systematic Reviews(9). doi:10.1002/14651858.CD005291.pub3
Lee, A., Arasaratnam, M., Chan, DLok Hang, Khasraw, M., Howell, V. M., & Wheeler, H. (2020). Anti‐epidermal growth factor receptor therapy for glioblastoma in adults. Cochrane Database of Systematic Reviews(5). doi:10.1002/14651858.CD013238.pub2
Schmidt‐Hansen, M., Baldwin, D. R., Hasler, E., Zamora, J., Abraira, V., & Roqué i Figuls, M. (2014). PET‐CT for assessing mediastinal lymph node involvement in patients with suspected resectable non‐small cell lung cancer. Cochrane Database of Systematic Reviews(11). doi:10.1002/14651858.CD009519.pub2
Sim, E. H. A., Yang, I. A., Wood‐Baker, R., Bowman, R. V., & Fong, K. M. (2018). Gefitinib for advanced non‐small cell lung cancer. Cochrane Database of Systematic Reviews(1). doi:10.1002/14651858.CD006847.pub2
Tattersall, A., Ryan, N., Wiggans, A. J., Rogozińska, E., & Morrison, J. (2022). Poly(ADP‐ribose) polymerase (PARP) inhibitors for the treatment of ovarian cancer. Cochrane Database of Systematic Reviews(2). doi:10.1002/14651858.CD007929.pub4
Williams, C., & Bryant, A. (2011). Short versus long duration infusions of paclitaxel for any advanced adenocarcinoma. Cochrane Database of Systematic Reviews(5). doi:10.1002/14651858.CD003911.pub2
Yang, Z. Y., Liu, L., Mao, C., Wu, X. Y., Huang, Y. F., Hu, X. F., & Tang, J. L. (2014). Chemotherapy with cetuximab versus chemotherapy alone for chemotherapy‐naive advanced non‐small cell lung cancer. Cochrane Database of Systematic Reviews(11). doi:10.1002/14651858.CD009948.pub2
