# Supplementary material for: Prognosis of recurrence after complete resection in early-stage lung adenocarcinoma based on molecular alterations: a systematic review and meta-analysis
Source: Sci Rep. 2023 Oct 31;13:18710. doi: 10.1038/s41598-023-42851-2 (PMC10618289; doi:10.1038/s41598-023-42851-2)
Supplement: Supplementary file 1 — Supplementary Information 1. [file 41598_2023_42851_MOESM1_ESM.zip › Additional Files/Keywords used and results for searching in different databases..docx]

| Database | Key words | Time | Result | Date |
| --- | --- | --- | --- | --- |
| Pubmed | ((lung adenocarcinoma) AND (recurrence)) AND ((genomic characteristics) OR (mutation)) | 2012-2022 | 659 | 2022-04-20 |
| Web of science | ((TS=(lung adenocarcinoma)) AND TS=(recurrence)) AND (TS=(genomic characteristics) OR TS=(mutational)) | 2012-2022 | 144 | 2022-04-20 |
| Cochrane Library | lung adenocarcinoma in All Text AND recurrence in All Text AND genomic characteristics in All Text | 2012-2022 | 9 | 2022-04-20 |

Additional Table S1: Keywords used and results for searching in different databases.
