# Supplementary material for: Prognosis of recurrence after complete resection in early-stage lung adenocarcinoma based on molecular alterations: a systematic review and meta-analysis
Source: Sci Rep. 2023 Oct 31;13:18710. doi: 10.1038/s41598-023-42851-2 (PMC10618289; doi:10.1038/s41598-023-42851-2)
Supplement: Supplementary file 1 — Supplementary Information 1. [file 41598_2023_42851_MOESM1_ESM.zip › Additional Files/pubmed-lung adenocarcinoma OR LUAD AND recurrence OR relapse AND mutation OR genomic characteristics.rtf]

Comprehensive genomic characterization of squamous cell lung cancers. (2012). Nature, 489(7417), 519-525. doi:10.1038/nature11404
Abdallah, N., Nagasaka, M., Abdulfatah, E., Shi, D., Wozniak, A. J., & Sukari, A. (2018). Non-small cell to small cell lung cancer on PD-1 inhibitors: two cases on potential histologic transformation. Lung Cancer (Auckl), 9, 85-90. doi:10.2147/lctt.S173724
Abdayem, P., & Planchard, D. (2021). Update on molecular pathology and role of liquid biopsy in nonsmall cell lung cancer. Eur Respir Rev, 30(161). doi:10.1183/16000617.0294-2020
AbdulJabbar, K., Raza, S. E. A., Rosenthal, R., Jamal-Hanjani, M., Veeriah, S., Akarca, A., . . . Yuan, Y. (2020). Geospatial immune variability illuminates differential evolution of lung adenocarcinoma. Nat Med, 26(7), 1054-1062. doi:10.1038/s41591-020-0900-x
Abe, M., Osoegawa, A., Karashima, T., Takumi, Y., Kobayashi, R., Hashimoto, T., . . . Sugio, K. (2019). Erlotinib and bevacizumab combination therapy for afatinib-refractory leptomeningeal carcinomatosis from EGFR-mutated lung cancer. Int Cancer Conf J, 8(2), 81-85. doi:10.1007/s13691-019-00358-6
Ahmad, Z., Raza, A., & Patel, M. R. (2015). Endometrial metastasis of lung adenocarcinoma: a report of two cases. Am J Case Rep, 16, 296-299. doi:10.12659/ajcr.892495
Akahori, D., Inoue, Y., Inui, N., Karayama, M., Yasui, H., Hozumi, H., . . . Suda, T. (2021). Comparative assessment of NOIR-SS and ddPCR for ctDNA detection of EGFR L858R mutations in advanced L858R-positive lung adenocarcinomas. Sci Rep, 11(1), 14999. doi:10.1038/s41598-021-94592-9
Akamatsu, H., Kaira, K., Murakami, H., Serizawa, M., Koh, Y., Ono, A., . . . Yamamoto, N. (2014). The impact of clinical outcomes according to EGFR mutation status in patients with locally advanced lung adenocarcinoma who recieved concurrent chemoradiotherapy. Am J Clin Oncol, 37(2), 144-147. doi:10.1097/COC.0b013e31826e04f9
Alidousty, C., Baar, T., Martelotto, L. G., Heydt, C., Wagener, S., Fassunke, J., . . . Schultheis, A. M. (2018). Genetic instability and recurrent MYC amplification in ALK-translocated NSCLC: a central role of TP53 mutations. J Pathol, 246(1), 67-76. doi:10.1002/path.5110
Alkassis, S., Alshare, B., & Ahmed, S. (2021). Maintained Complete Response and Long-Term Survival in Epidermal Growth Factor Receptor Mutated Metastatic Non-Small Cell Lung Cancer with Erlotinib. Cureus, 13(1), e12451. doi:10.7759/cureus.12451
Allen, J. M., Schrock, A. B., Erlich, R. L., Miller, V. A., Stephens, P. J., Ross, J. S., . . . Vafai, D. (2017). Genomic Profiling of Circulating Tumor DNA in Relapsed EGFR-mutated Lung Adenocarcinoma Reveals an Acquired FGFR3-TACC3 Fusion. Clin Lung Cancer, 18(3), e219-e222. doi:10.1016/j.cllc.2016.12.006
Alqaidy, D., & Moran, C. A. (2021). Primary Warthin's-like adenocarcinoma of the lung: A clinicopathological, immunohistochemical, and molecular analysis of three cases. Pathol Res Pract, 227, 153648. doi:10.1016/j.prp.2021.153648
Andreasen, S., Persson, M., Kiss, K., Homøe, P., Heegaard, S., & Stenman, G. (2016). Genomic profiling of a combined large cell neuroendocrine carcinoma of the submandibular gland. Oncol Rep, 35(4), 2177-2182. doi:10.3892/or.2016.4621
Aokage, K., Miyoshi, T., Wakabayashi, M., Ikeno, T., Suzuki, J., Tane, K., . . . Tsuboi, M. (2021). Prognostic influence of epidermal growth factor receptor mutation and radiological ground glass appearance in patients with early-stage lung adenocarcinoma. Lung Cancer, 160, 8-16. doi:10.1016/j.lungcan.2021.07.018
Aoki, M., Ueda, K., Umehara, T., Kamimura, G. O., Tokunaga, T., Harada-Takeda, A., . . . Sato, M. (2020). Targeted Therapy Followed by Cytotoxic Chemotherapy in Preoperative Patients With Locally Advanced Lung Adenocarcinoma. Anticancer Res, 40(5), 2911-2916. doi:10.21873/anticanres.14268
Aomatsu, N., Maeda, K., Uchima, Y., Matsutani, S., Tsujio, G., Miyamoto, H., . . . Takeuchi, K. (2021). Multiple stage IV colorectal cancers in a patient who received multidisciplinary treatment, including chemotherapy and Japanese Kampo medicine: A case report. Neuropeptides, 88, 102160. doi:10.1016/j.npep.2021.102160
Arcila, M. E., Chaft, J. E., Nafa, K., Roy-Chowdhuri, S., Lau, C., Zaidinski, M., . . . Ladanyi, M. (2012). Prevalence, clinicopathologic associations, and molecular spectrum of ERBB2 (HER2) tyrosine kinase mutations in lung adenocarcinomas. Clin Cancer Res, 18(18), 4910-4918. doi:10.1158/1078-0432.Ccr-12-0912
Arcila, M. E., Drilon, A., Sylvester, B. E., Lovly, C. M., Borsu, L., Reva, B., . . . Ladanyi, M. (2015). MAP2K1 (MEK1) Mutations Define a Distinct Subset of Lung Adenocarcinoma Associated with Smoking. Clin Cancer Res, 21(8), 1935-1943. doi:10.1158/1078-0432.Ccr-14-2124
Aydiner, A., Yildiz, I., & Seyidova, A. (2013). Clinical outcomes and prognostic factors associated with the response to erlotinib in non-small-cell lung cancer patients with unknown EGFR mutational status. Asian Pac J Cancer Prev, 14(5), 3255-3261. doi:10.7314/apjcp.2013.14.5.3255
Bae, K., Kim, J. H., Jung, H., Kong, S. Y., Kim, Y. H., Kim, S., . . . Yoon, K. A. (2021). A fusion of CD63-BCAR4 identified in lung adenocarcinoma promotes tumorigenicity and metastasis. Br J Cancer, 124(1), 290-298. doi:10.1038/s41416-020-01146-3
Becker-Santos, D. D., Thu, K. L., English, J. C., Pikor, L. A., Martinez, V. D., Zhang, M., . . . Lam, W. L. (2016). Developmental transcription factor NFIB is a putative target of oncofetal miRNAs and is associated with tumour aggressiveness in lung adenocarcinoma. J Pathol, 240(2), 161-172. doi:10.1002/path.4765
Behrens, C., Solis, L. M., Lin, H., Yuan, P., Tang, X., Kadara, H., . . . Wistuba, II. (2013). EZH2 protein expression associates with the early pathogenesis, tumor progression, and prognosis of non-small cell lung carcinoma. Clin Cancer Res, 19(23), 6556-6565. doi:10.1158/1078-0432.Ccr-12-3946
Bennett, L., Howell, M., Memon, D., Smowton, C., Zhou, C., & Miller, C. J. (2018). Mutation pattern analysis reveals polygenic mini-drivers associated with relapse after surgery in lung adenocarcinoma. Sci Rep, 8(1), 14830. doi:10.1038/s41598-018-33276-3
Bie, Y., Wang, J., Xiong, L., Wang, D., Liao, J., Zhang, Y., & Lin, H. (2021). Lung adenocarcinoma organoids harboring EGFR 19Del and L643V double mutations respond to osimertinib and gefitinib: A case report. Medicine (Baltimore), 100(11), e24793. doi:10.1097/md.0000000000024793
Bittner, N., Balikó, Z., Sárosi, V., László, T., Tóth, E., Kásler, M., & Géczi, L. (2015). Bone Metastases and the EGFR and KRAS Mutation Status in Lung Adenocarcinoma--The Results of Three Year Retrospective Analysis. Pathol Oncol Res, 21(4), 1217-1221. doi:10.1007/s12253-015-9955-2
Bjaanæs, M. M., Nilsen, G., Halvorsen, A. R., Russnes, H. G., Solberg, S., Jørgensen, L., . . . Helland, Å. (2021). Whole genome copy number analyses reveal a highly aberrant genome in TP53 mutant lung adenocarcinoma tumors. BMC Cancer, 21(1), 1089. doi:10.1186/s12885-021-08811-7
Blackhall, F., Thatcher, N., Booton, R., & Kerr, K. (2013). The impact on the multidisciplinary team of molecular profiling for personalized therapy in non-small cell lung cancer. Lung Cancer, 79(2), 101-103. doi:10.1016/j.lungcan.2012.10.016
Bleau, A. M., Freire, J., Pajares, M. J., Zudaire, I., Anton, I., Nistal-Villán, E., . . . Montuenga, L. M. (2014). New syngeneic inflammatory-related lung cancer metastatic model harboring double KRAS/WWOX alterations. Int J Cancer, 135(11), 2516-2527. doi:10.1002/ijc.28574
Bos, M., Gardizi, M., Schildhaus, H. U., Heukamp, L. C., Geist, T., Kaminsky, B., . . . Wolf, J. (2013). Complete metabolic response in a patient with repeatedly relapsed non-small cell lung cancer harboring ROS1 gene rearrangement after treatment with crizotinib. Lung Cancer, 81(1), 142-143. doi:10.1016/j.lungcan.2013.02.018
Brooks, A. N., Choi, P. S., de Waal, L., Sharifnia, T., Imielinski, M., Saksena, G., . . . Meyerson, M. (2014). A pan-cancer analysis of transcriptome changes associated with somatic mutations in U2AF1 reveals commonly altered splicing events. PLoS One, 9(1), e87361. doi:10.1371/journal.pone.0087361
Brunner, A. M., Costa, D. B., Heist, R. S., Garcia, E., Lindeman, N. I., Sholl, L. M., . . . Hammerman, P. S. (2013). Treatment-related toxicities in a phase II trial of dasatinib in patients with squamous cell carcinoma of the lung. J Thorac Oncol, 8(11), 1434-1437. doi:10.1097/JTO.0b013e3182a47162
Bulutay, P., AkyÜrek, N., & MemiŞ, L. (2021). Clinicopathological and Prognostic Significance of the EML4-ALK Translocation and IGFR1, TTF1, Napsin A Expression in Patients with Lung Adenocarcinoma. Turk Patoloji Derg, 37(1), 7-17. doi:10.5146/tjpath.2020.01503
Burkart, J., Shilo, K., Zhao, W., Ozkan, E., Ajam, A., & Otterson, G. A. (2015). Metastatic Squamous Cell Carcinoma Component from an Adenosquamous Carcinoma of the Lung with Identical Epidermal Growth Factor Receptor Mutations. Case Rep Pulmonol, 2015, 283875. doi:10.1155/2015/283875
Cai, D., Li, H., Wang, R., Li, Y., Pan, Y., Hu, H., . . . Chen, H. (2014). Comparison of clinical features, molecular alterations, and prognosis in morphological subgroups of lung invasive mucinous adenocarcinoma. Onco Targets Ther, 7, 2127-2132. doi:10.2147/ott.S70984
Cai, G., Wong, R., Chhieng, D., Levy, G. H., Gettinger, S. N., Herbst, R. S., . . . Hui, P. (2013). Identification of EGFR mutation, KRAS mutation, and ALK gene rearrangement in cytological specimens of primary and metastatic lung adenocarcinoma. Cancer Cytopathol, 121(9), 500-507. doi:10.1002/cncy.21288
Cai, L., Wang, J., Yan, J., Zeng, J., Zhu, L., Liang, J., . . . Jiang, Y. (2020). Genomic Profiling and Prognostic Value Analysis of Genetic Alterations in Chinese Resected Lung Cancer With Invasive Mucinous Adenocarcinoma. Front Oncol, 10, 603671. doi:10.3389/fonc.2020.603671
Campbell, J. D., Alexandrov, A., Kim, J., Wala, J., Berger, A. H., Pedamallu, C. S., . . . Meyerson, M. (2016). Distinct patterns of somatic genome alterations in lung adenocarcinomas and squamous cell carcinomas. Nat Genet, 48(6), 607-616. doi:10.1038/ng.3564
Cao, Y., Zhu, L. Z., Jiang, M. J., & Yuan, Y. (2016). Clinical impacts of a micropapillary pattern in lung adenocarcinoma: a review. Onco Targets Ther, 9, 149-158. doi:10.2147/ott.S94747
Capelletti, M., Dodge, M. E., Ercan, D., Hammerman, P. S., Park, S. I., Kim, J., . . . Jänne, P. A. (2014). Identification of recurrent FGFR3-TACC3 fusion oncogenes from lung adenocarcinoma. Clin Cancer Res, 20(24), 6551-6558. doi:10.1158/1078-0432.Ccr-14-1337
Cardarella, S., Ortiz, T. M., Joshi, V. A., Butaney, M., Jackman, D. M., Kwiatkowski, D. J., . . . Johnson, B. E. (2012). The introduction of systematic genomic testing for patients with non-small-cell lung cancer. J Thorac Oncol, 7(12), 1767-1774. doi:10.1097/JTO.0b013e3182745bcb
Cardoso, M., Paulo, P., Maia, S., & Teixeira, M. R. (2016). Truncating and missense PPM1D mutations in early-onset and/or familial/hereditary prostate cancer patients. Genes Chromosomes Cancer, 55(12), 954-961. doi:10.1002/gcc.22393
Carrot-Zhang, J., Yao, X., Devarakonda, S., Deshpande, A., Damrauer, J. S., Silva, T. C., . . . Imielinski, M. (2021). Whole-genome characterization of lung adenocarcinomas lacking the RTK/RAS/RAF pathway. Cell Rep, 34(5), 108707. doi:10.1016/j.celrep.2021.108707
Caso, R., Sanchez-Vega, F., Tan, K. S., Mastrogiacomo, B., Zhou, J., Jones, G. D., . . . Jones, D. R. (2020). The Underlying Tumor Genomics of Predominant Histologic Subtypes in Lung Adenocarcinoma. J Thorac Oncol, 15(12), 1844-1856. doi:10.1016/j.jtho.2020.08.005
Cassidy, R. J., Zhang, X., Patel, P. R., Shelton, J. W., Escott, C. E., Sica, G. L., . . . Higgins, K. A. (2017). Next-generation sequencing and clinical outcomes of patients with lung adenocarcinoma treated with stereotactic body radiotherapy. Cancer, 123(19), 3681-3690. doi:10.1002/cncr.30794
Cha, Y. J., Kim, H. R., Lee, C. Y., Cho, B. C., & Shim, H. S. (2016). Clinicopathological and prognostic significance of programmed cell death ligand-1 expression in lung adenocarcinoma and its relationship with p53 status. Lung Cancer, 97, 73-80. doi:10.1016/j.lungcan.2016.05.001
Chaft, J. E., Litvak, A., Arcila, M. E., Patel, P., D'Angelo, S. P., Krug, L. M., . . . Azzoli, C. G. (2014). Phase II study of the GI-4000 KRAS vaccine after curative therapy in patients with stage I-III lung adenocarcinoma harboring a KRAS G12C, G12D, or G12V mutation. Clin Lung Cancer, 15(6), 405-410. doi:10.1016/j.cllc.2014.06.002
Chalela, R., Bellosillo, B., Curull, V., Longarón, R., Pascual-Guardia, S., Badenes-Bonet, D., . . . Gea, J. (2019). EGFR and KRAS Mutations in the Non-Tumoral Lung. Prognosis in Patients with Adenocarcinoma. J Clin Med, 8(4). doi:10.3390/jcm8040529
Chang, J. C., Alex, D., Bott, M., Tan, K. S., Seshan, V., Golden, A., . . . Rekhtman, N. (2019). Comprehensive Next-Generation Sequencing Unambiguously Distinguishes Separate Primary Lung Carcinomas From Intrapulmonary Metastases: Comparison with Standard Histopathologic Approach. Clin Cancer Res, 25(23), 7113-7125. doi:10.1158/1078-0432.Ccr-19-1700
Chang, J. C., Montecalvo, J., Borsu, L., Lu, S., Larsen, B. T., Wallace, W. D., . . . Rekhtman, N. (2018). Bronchiolar Adenoma: Expansion of the Concept of Ciliated Muconodular Papillary Tumors With Proposal for Revised Terminology Based on Morphologic, Immunophenotypic, and Genomic Analysis of 25 Cases. Am J Surg Pathol, 42(8), 1010-1026. doi:10.1097/pas.0000000000001086
Chang, J. C., Offin, M., Falcon, C., Brown, D., Houck-Loomis, B. R., Meng, F., . . . Rekhtman, N. (2021). Comprehensive Molecular and Clinicopathologic Analysis of 200 Pulmonary Invasive Mucinous Adenocarcinomas Identifies Distinct Characteristics of Molecular Subtypes. Clin Cancer Res, 27(14), 4066-4076. doi:10.1158/1078-0432.Ccr-21-0423
Chang, W. C., Zhang, Y. Z., Wolf, J. L., Hermelijn, S. M., Schnater, J. M., von der Thüsen, J. H., . . . Nicholson, A. G. (2021). Mucinous adenocarcinoma arising in congenital pulmonary airway malformation: clinicopathological analysis of 37 cases. Histopathology, 78(3), 434-444. doi:10.1111/his.14239
Chen, C., Tang, Y., Qu, W. D., Han, X., Zuo, J. B., Cai, Q. Y., . . . Ke, X. X. (2021). Evaluation of clinical value and potential mechanism of MTFR2 in lung adenocarcinoma via bioinformatics. BMC Cancer, 21(1), 619. doi:10.1186/s12885-021-08378-3
Chen, C., Zhou, P., Zhang, Z., & Liu, Y. (2022). U2AF1 mutation Connects DNA Damage to the Alternative Splicing of RAD51 in Lung Adenocarcinomas. Clin Exp Pharmacol Physiol. doi:10.1111/1440-1681.13646
Chen, K., Chen, W., Cai, J., Yang, F., Lou, F., Wang, X., . . . Wang, J. (2018). Favorable prognosis and high discrepancy of genetic features in surgical patients with multiple primary lung cancers. J Thorac Cardiovasc Surg, 155(1), 371-379.e371. doi:10.1016/j.jtcvs.2017.08.141
Chen, K., Zhao, H., Shi, Y., Yang, F., Wang, L. T., Kang, G., . . . Wang, J. (2019). Perioperative Dynamic Changes in Circulating Tumor DNA in Patients with Lung Cancer (DYNAMIC). Clin Cancer Res, 25(23), 7058-7067. doi:10.1158/1078-0432.Ccr-19-1213
Chen, M., Zhang, J., Berger, A. H., Diolombi, M. S., Ng, C., Fung, J., . . . Pandolfi, P. P. (2019). Compound haploinsufficiency of Dok2 and Dusp4 promotes lung tumorigenesis. J Clin Invest, 129(1), 215-222. doi:10.1172/jci99699
Chen, R., Yang, W., Li, Y., Cheng, X., Nie, Y., Liu, D., & Wang, H. (2022). Effect of immunotherapy on the immune microenvironment in advanced recurrent cervical cancer. Int Immunopharmacol, 106, 108630. doi:10.1016/j.intimp.2022.108630
Chen, S., Yang, S., Zhang, Y., Xiang, J., Zhang, Y., Hu, H., . . . Ye, T. (2021). Clinicopathologic features and prognostic value of epidermal growth factor receptor mutation in patients with pT1a and pT1b invasive lung adenocarcinoma after surgical resection. J Thorac Dis, 13(9), 5496-5507. doi:10.21037/jtd-21-924
Chen, W., Lin, Y., Yu, Y., Wei, P., & Dai, H. (2016). Recurrent bilateral spontaneous pneumothorax secondary to lung adenocarcinoma with epidermal growth factor receptor mutation. Thorac Cancer, 7(2), 257-260. doi:10.1111/1759-7714.12292
Chen, Z., Chen, G., Wang, Z., Yu, J., Zhang, H., Mao, B., & Ma, H. (2021). Comparative molecular profiling of distant metastatic and non-distant metastatic lung adenocarcinoma. Neoplasma, 68(2), 253-261. doi:10.4149/neo_2020_200203N103
Cheng, C., Wang, R., Li, Y., Pan, Y., Zhang, Y., Li, H., . . . Chen, H. (2015). EGFR Exon 18 Mutations in East Asian Patients with Lung Adenocarcinomas: A Comprehensive Investigation of Prevalence, Clinicopathologic Characteristics and Prognosis. Sci Rep, 5, 13959. doi:10.1038/srep13959
Cheng, Y., Wang, Y., Zhao, J., Liu, Y., Gao, H., Ma, K., . . . Li, B. (2018). Real-world EGFR testing in patients with stage IIIB/IV non-small-cell lung cancer in North China: A multicenter, non-interventional study. Thorac Cancer, 9(11), 1461-1469. doi:10.1111/1759-7714.12859
Chiou, J., Chang, Y. C., Jan, Y. H., Tsai, H. F., Yang, C. J., Huang, M. S., . . . Hsiao, M. (2019). Overexpression of BZW1 is an independent poor prognosis marker and its down-regulation suppresses lung adenocarcinoma metastasis. Sci Rep, 9(1), 14624. doi:10.1038/s41598-019-50874-x
Choi, H., Kratz, J., Pham, P., Lee, S., Ray, R., Kwon, Y. W., . . . Kim, I. J. (2012). Development of a rapid and practical mutation screening assay for human lung adenocarcinoma. Int J Oncol, 40(6), 1900-1906. doi:10.3892/ijo.2012.1396
Choi, M. K., Hong, J. Y., Chang, W. J., Kim, M. J., Kim, S. M., Jung, H. A., . . . Ahn, M. J. (2015). A phase II trial of gefitinib monotherapy in pretreated patients with advanced non-small cell lung cancer not harboring activating EGFR mutations: implications of sensitive EGFR mutation test. Cancer Chemother Pharmacol, 75(6), 1229-1236. doi:10.1007/s00280-015-2740-9
Choi, Y., Kim, K. H., Jeong, B. H., Lee, K. J., Kim, H., Kwon, O. J., . . . Um, S. W. (2020). Clinicoradiopathological features and prognosis according to genomic alterations in patients with resected lung adenocarcinoma. J Thorac Dis, 12(10), 5357-5368. doi:10.21037/jtd-20-1716
Cihoric, N., Savic, S., Schneider, S., Ackermann, I., Bichsel-Naef, M., Schmid, R. A., . . . Tapia, C. (2014). Prognostic role of FGFR1 amplification in early-stage non-small cell lung cancer. Br J Cancer, 110(12), 2914-2922. doi:10.1038/bjc.2014.229
Cirauqui, B., Morán, T., Estival, A., Quiroga, V., Etxaniz, O., Balana, C., . . . Margelí, M. (2020). Breast Cancer Patient with Li-Fraumeni Syndrome: A Case Report Highlighting the Importance of Multidisciplinary Management. Case Rep Oncol, 13(1), 130-138. doi:10.1159/000505684
Clifford, H. W., Cassidy, A. P., Vaughn, C., Tsai, E. S., Seres, B., Patel, N., . . . Cassidy, J. W. (2016). Profiling lung adenocarcinoma by liquid biopsy: can one size fit all? Cancer Nanotechnol, 7(1), 10. doi:10.1186/s12645-016-0023-8
Cooper, W. A., Lam, D. C., O'Toole, S. A., & Minna, J. D. (2013). Molecular biology of lung cancer. J Thorac Dis, 5 Suppl 5(Suppl 5), S479-490. doi:10.3978/j.issn.2072-1439.2013.08.03
Crow, M., Lim, N., Ballouz, S., Pavlidis, P., & Gillis, J. (2019). Predictability of human differential gene expression. Proc Natl Acad Sci U S A, 116(13), 6491-6500. doi:10.1073/pnas.1802973116
Cui, M., Han, Y., Li, P., Zhang, J., Ou, Q., Tong, X., . . . Jiang, G. (2020). Molecular and clinicopathological characteristics of ROS1-rearranged non-small-cell lung cancers identified by next-generation sequencing. Mol Oncol, 14(11), 2787-2795. doi:10.1002/1878-0261.12789
Dandachi, N., Tiran, V., Lindenmann, J., Brcic, L., Fink-Neuboeck, N., Kashofer, K., . . . Balic, M. (2017). Frequency and clinical impact of preoperative circulating tumor cells in resectable non-metastatic lung adenocarcinomas. Lung Cancer, 113, 152-157. doi:10.1016/j.lungcan.2017.10.003
D'Angelo, S. P., Janjigian, Y. Y., Ahye, N., Riely, G. J., Chaft, J. E., Sima, C. S., . . . Azzoli, C. G. (2012). Distinct clinical course of EGFR-mutant resected lung cancers: results of testing of 1118 surgical specimens and effects of adjuvant gefitinib and erlotinib. J Thorac Oncol, 7(12), 1815-1822. doi:10.1097/JTO.0b013e31826bb7b2
Das, B. R., Bhaumik, S., Ahmad, F., Mandsaurwala, A., & Satam, H. (2015). Molecular spectrum of somatic EGFR and KRAS gene mutations in non small cell lung carcinoma: determination of frequency, distribution pattern and identification of novel variations in Indian patients. Pathol Oncol Res, 21(3), 675-687. doi:10.1007/s12253-014-9874-7
Davis, R., Deak, K., & Glass, C. H. (2019). Pulmonary Granular Cell Tumors: A Study of 4 Cases Including a Malignant Phenotype. Am J Surg Pathol, 43(10), 1397-1402. doi:10.1097/pas.0000000000001303
De Martino, L., Errico, M. E., Ruotolo, S., Cascone, D., Chiaravalli, S., Collini, P., . . . Quaglietta, L. (2018). Pediatric lung adenocarcinoma presenting with brain metastasis: a case report. J Med Case Rep, 12(1), 243. doi:10.1186/s13256-018-1781-1
Deli, T., Orosz, M., & Jakab, A. (2020). Hormone Replacement Therapy in Cancer Survivors - Review of the Literature. Pathol Oncol Res, 26(1), 63-78. doi:10.1007/s12253-018-00569-x
Deng, C., Zhang, Y., Fu, F., Ma, X., Wen, Z., Ma, Z., . . . Chen, H. (2021). Genetic-pathological prediction for timing and site-specific recurrence pattern in resected lung adenocarcinoma. Eur J Cardiothorac Surg, 60(5), 1223-1231. doi:10.1093/ejcts/ezab288
Deng, C., Zhang, Y., Ma, Z., Fu, F., Deng, L., Li, Y., & Chen, H. (2021). Prognostic value of epidermal growth factor receptor gene mutation in resected lung adenocarcinoma. J Thorac Cardiovasc Surg, 162(3), 664-674.e667. doi:10.1016/j.jtcvs.2020.05.099
Deng, C., Zheng, Q., Zhang, Y., Jin, Y., Shen, X., Nie, X., . . . Chen, H. (2021). Validation of the Novel International Association for the Study of Lung Cancer Grading System for Invasive Pulmonary Adenocarcinoma and Association With Common Driver Mutations. J Thorac Oncol, 16(10), 1684-1693. doi:10.1016/j.jtho.2021.07.006
Deng, Z. M., Liu, L., Qiu, W. H., Zhang, Y. Q., Zhong, H. Y., Liao, P., & Wu, Y. H. (2017). Analysis of genomic variation in lung adenocarcinoma patients revealed the critical role of PI3K complex. PeerJ, 5, e3216. doi:10.7717/peerj.3216
Devarakonda, S., Pellini, B., Verghese, L., Park, H., Morgensztern, D., Govindan, R., . . . Waqar, S. N. (2021). A phase II study of everolimus in patients with advanced solid malignancies with TSC1, TSC2, NF1, NF2 or STK11 mutations. J Thorac Dis, 13(7), 4054-4062. doi:10.21037/jtd-21-195
Di Maio, M., Leighl, N. B., Gallo, C., Feld, R., Ciardiello, F., Butts, C., . . . Gridelli, C. (2012). Quality of life analysis of TORCH, a randomized trial testing first-line erlotinib followed by second-line cisplatin/gemcitabine chemotherapy in advanced non-small-cell lung cancer. J Thorac Oncol, 7(12), 1830-1844. doi:10.1097/JTO.0b013e318275b327
Dimou, A., Barron, G., Merrick, D. T., Kolfenbach, J., & Doebele, R. C. (2020). Granulomatosis with polyangiitis in a patient treated with dabrafenib and trametinib for BRAF V600E positive lung adenocarcinoma. BMC Cancer, 20(1), 177. doi:10.1186/s12885-020-6661-6
Dong, S., Zhao, N., Deng, W., Sun, H. W., Niu, F. Y., Yang, J. J., . . . Wu, Y. L. (2017). Supraclavicular lymph node incisional biopsies have no influence on the prognosis of advanced non-small cell lung cancer patients: a retrospective study. World J Surg Oncol, 15(1), 12. doi:10.1186/s12957-016-1064-5
Dong, Y., Li, Q., Miao, Q., & Li, D. (2021). Erlotinib as a salvage treatment after gefitinib failure for advanced non-small-cell lung cancer patients with brain metastasis: A successful case report and review. Medicine (Baltimore), 100(25), e26450. doi:10.1097/md.0000000000026450
Dong, Y., Liu, Y., Bai, H., & Jiao, S. (2019). Systematic assessment of the clinicopathological prognostic significance of tissue cytokine expression for lung adenocarcinoma based on integrative analysis of TCGA data. Sci Rep, 9(1), 6301. doi:10.1038/s41598-019-42345-0
Dou, Y., Duan, Q., Qi, C., Hou, L., & Wang, H. (2021). An intergenic region ALK fusion identified by DNA sequencing and validated by IHC in an early-stage lung adenocarcinoma. J Cancer Res Clin Oncol, 147(6), 1865-1867. doi:10.1007/s00432-021-03526-5
Durand, M., Logerot, S., Fonrose, X., & Schir, E. (2014). [Treatment with erlotinib after gefitinib induced hepatotoxicity: literature review and case report]. Therapie, 69(2), 163-168. doi:10.2515/therapie/2014017
Eguchi, T., Kadota, K., Park, B. J., Travis, W. D., Jones, D. R., & Adusumilli, P. S. (2014). The new IASLC-ATS-ERS lung adenocarcinoma classification: what the surgeon should know. Semin Thorac Cardiovasc Surg, 26(3), 210-222. doi:10.1053/j.semtcvs.2014.09.002
El Jabbour, T., Dalvi, S. D., Kim, S., Sheehan, C., & Ross, J. S. (2018). Myeloid cell leukemia-1 protein expression and myeloid cell leukemia-1 gene amplification in non small cell lung cancer. Indian J Pathol Microbiol, 61(1), 27-30. doi:10.4103/ijpm.Ijpm_731_16
Errico, A. (2014). Lung cancer: Heterogeneity in space and time. Nat Rev Clin Oncol, 11(12), 684. doi:10.1038/nrclinonc.2014.186
Fan, L., & He, P. (2022). [Research Progress on Spread Through Air Spaces of Lung Cancer]. Zhongguo Fei Ai Za Zhi, 25(1), 54-60. doi:10.3779/j.issn.1009-3419.2021.101.49
Fan, P. D., Narzisi, G., Jayaprakash, A. D., Venturini, E., Robine, N., Smibert, P., . . . Ladanyi, M. (2018). YES1 amplification is a mechanism of acquired resistance to EGFR inhibitors identified by transposon mutagenesis and clinical genomics. Proc Natl Acad Sci U S A, 115(26), E6030-e6038. doi:10.1073/pnas.1717782115
Fang, W., Zhao, S., Liang, Y., Yang, Y., Yang, L., Dong, X., . . . Zhang, L. (2019). Mutation Variants and Co-Mutations as Genomic Modifiers of Response to Afatinib in HER2-Mutant Lung Adenocarcinoma. Oncologist. doi:10.1634/theoncologist.2019-0547
Fang, W., Zhao, S., Liang, Y., Yang, Y., Yang, L., Dong, X., . . . Zhang, L. (2020). Mutation Variants and Co-Mutations as Genomic Modifiers of Response to Afatinib in HER2-Mutant Lung Adenocarcinoma. Oncologist, 25(3), e545-e554. doi:10.1634/theoncologist.2019-0547
Fekete, G. L., & Fekete, L. (2019). Cutaneous leukocytoclastic vasculitis associated with erlotinib treatment: A case report and review of the literature. Exp Ther Med, 17(2), 1128-1131. doi:10.3892/etm.2018.6988
Feng, Y., Zhong, M., Zeng, S., Xiao, D., & Liu, Y. (2018). Metachronous triple primary neoplasms with primary prostate cancer, lung cancer, and colon cancer: A case report. Medicine (Baltimore), 97(26), e11332. doi:10.1097/md.0000000000011332
Ferguson, M. D., Dong, L., Wan, J., Deneve, J. L., Dickson, P. V., Behrman, S. W., . . . Glazer, E. S. (2019). Molecular Alterations Associated with DNA Repair in Pancreatic Adenocarcinoma Are Associated with Sites of Recurrence. J Gastrointest Cancer, 50(2), 285-291. doi:10.1007/s12029-018-0073-8
Finn, S. P., Addeo, A., Dafni, U., Thunnissen, E., Bubendorf, L., Madsen, L. B., . . . Stahel, R. A. (2021). Prognostic Impact of KRAS G12C Mutation in Patients With NSCLC: Results From the European Thoracic Oncology Platform Lungscape Project. J Thorac Oncol, 16(6), 990-1002. doi:10.1016/j.jtho.2021.02.016
Frampton, G. M., Ali, S. M., Rosenzweig, M., Chmielecki, J., Lu, X., Bauer, T. M., . . . Miller, V. A. (2015). Activation of MET via diverse exon 14 splicing alterations occurs in multiple tumor types and confers clinical sensitivity to MET inhibitors. Cancer Discov, 5(8), 850-859. doi:10.1158/2159-8290.Cd-15-0285
Fujibayashi, Y., Tane, S., Kitazume, M., Kuroda, S., Kimura, K., Kitamura, Y., & Nishio, W. (2022). Resected stage I anaplastic lymphoma kinase-positive lung adenocarcinoma has a negative impact on recurrence-free survival. Thorac Cancer, 13(8), 1109-1116. doi:10.1111/1759-7714.14365
Fujii, T., Ohno, N., Matsui, T., Kitahara, S., Matsunaga, T., Sahara, N., . . . Kobayash, S. (2019). [A Patient Surviving More Than Five Years Due to the Effect of Sequential Alternating Chemotherapy Administered to Metachronous Overlapping Cancer of Multiple Myeloma and StageⅣ EGFR Positive Non-Small Cell Lung Cancer]. Gan To Kagaku Ryoho, 46(12), 1899-1902. 
Fujikawa, R., Muraoka, Y., Kashima, J., Yoshida, Y., Ito, K., Watanabe, H., . . . Yatabe, Y. (2022). Clinicopathologic and Genotypic Features of Lung Adenocarcinoma Characterized by the IASLC Grading System. J Thorac Oncol. doi:10.1016/j.jtho.2022.02.005
Fujiu, K., Kobayashi, N., Miyamoto, H., & Suzuki, H. (2012). A case of choroidal metastasis of lung cancer successfully treated with erlotinib. Gan To Kagaku Ryoho, 39(2), 269-271. 
Fujiwara, A., Yoshida, M., Fujimoto, H., Nakahara, H., Ito, K., Nishihama, K., . . . Kobayashi, T. (2018). A Retrospective Comparison of the Clinical Efficacy of Gefitinib, Erlotinib, and Afatinib in Japanese Patients With Non-Small Cell Lung Cancer. Oncol Res, 26(7), 1031-1036. doi:10.3727/096504018x15151523767752
Fujiwara, T., Nakajima, T., Inage, T., Sata, Y., Yamamoto, T., Sakairi, Y., . . . Yoshino, I. (2021). Endobronchial ultrasound-guided transbronchial needle aspiration in patients with previously treated lung cancer. Surg Today, 51(3), 415-421. doi:10.1007/s00595-020-02101-8
Furrukh, M., Kumar, S., Zahid, K. F., Al-Shamly, H. S., Al-Jabri, Z. A., Burney, I. A., & Al-Moundhri, M. S. (2017). Trends and Outcomes of Non-Small-Cell Lung Cancer in Omani Patients: Experience at a university hospital. Sultan Qaboos Univ Med J, 17(3), e301-e308. doi:10.18295/squmj.2017.17.03.007
Gallant, J. N., Sheehan, J. H., Shaver, T. M., Bailey, M., Lipson, D., Chandramohan, R., . . . Lovly, C. M. (2015). EGFR Kinase Domain Duplication (EGFR-KDD) Is a Novel Oncogenic Driver in Lung Cancer That Is Clinically Responsive to Afatinib. Cancer Discov, 5(11), 1155-1163. doi:10.1158/2159-8290.Cd-15-0654
Galvez, C., Jacob, S., Finkelman, B. S., Zhao, J., Tegtmeyer, K., Chae, Y. K., . . . Villaflor, V. (2020). The role of EGFR mutations in predicting recurrence in early and locally advanced lung adenocarcinoma following definitive therapy. Oncotarget, 11(21), 1953-1960. doi:10.18632/oncotarget.27602
Gao, X., Deeb, D., Liu, Y., Liu, P., Zhang, Y., Shaw, J., & Gautam, S. C. (2015). CDDO-Me inhibits tumor growth and prevents recurrence of pancreatic ductal adenocarcinoma. Int J Oncol, 47(6), 2100-2106. doi:10.3892/ijo.2015.3212
Gao, X., Zhao, Y., Bao, Y., Yin, W., Liu, L., Liu, R., . . . Shuai, J. (2019). Poor Prognosis With Coexistence Of EGFR T790M Mutation And Common EGFR-Activating Mutation In Non- Small Cell Lung Cancer. Cancer Manag Res, 11, 9621-9630. doi:10.2147/cmar.S216721
Ge, J., Yao, B., Huang, J., Wu, X., Bao, H., Ou, Q., . . . Chen, J. (2019). Molecular genetic characterization reveals linear tumor evolution in a pulmonary sarcomatoid carcinomas patient with a novel PHF20-NTRK1 fusion: a case report. BMC Cancer, 19(1), 592. doi:10.1186/s12885-019-5780-4
Goeman, F., De Nicola, F., Scalera, S., Sperati, F., Gallo, E., Ciuffreda, L., . . . Maugeri-Saccà, M. (2019). Mutations in the KEAP1-NFE2L2 Pathway Define a Molecular Subset of Rapidly Progressing Lung Adenocarcinoma. J Thorac Oncol, 14(11), 1924-1934. doi:10.1016/j.jtho.2019.07.003
Gong, J., Gregg, J. P., Ma, W., Yoneda, K., Moore, E. H., Daly, M. E., . . . Li, T. (2019). Squamous Cell Transformation of Primary Lung Adenocarcinoma in a Patient With EML4-ALK Fusion Variant 5 Refractory to ALK Inhibitors. J Natl Compr Canc Netw, 17(4), 297-301. doi:10.6004/jnccn.2019.7291
Gong, X., Yi, J., Carmon, K. S., Crumbley, C. A., Xiong, W., Thomas, A., . . . Liu, Q. J. (2015). Aberrant RSPO3-LGR4 signaling in Keap1-deficient lung adenocarcinomas promotes tumor aggressiveness. Oncogene, 34(36), 4692-4701. doi:10.1038/onc.2014.417
Gordon, M. A., Babbs, B., Cochrane, D. R., Bitler, B. G., & Richer, J. K. (2019). The long non-coding RNA MALAT1 promotes ovarian cancer progression by regulating RBFOX2-mediated alternative splicing. Mol Carcinog, 58(2), 196-205. doi:10.1002/mc.22919
Govindan, R. (2014). Cancer. Attack of the clones. Science, 346(6206), 169-170. doi:10.1126/science.1259926
Gow, C. H., Hsieh, M. S., Liu, Y. N., Lee, Y. H., & Shih, J. Y. (2021). Clinicopathological Features and Survival Outcomes of Primary Pulmonary Invasive Mucinous Adenocarcinoma. Cancers (Basel), 13(16). doi:10.3390/cancers13164103
Gow, C. H., Hsieh, M. S., Wu, S. G., & Shih, J. Y. (2017). A comprehensive analysis of clinical outcomes in lung cancer patients harboring a MET exon 14 skipping mutation compared to other driver mutations in an East Asian population. Lung Cancer, 103, 82-89. doi:10.1016/j.lungcan.2016.12.001
Graham, R. P., Treece, A. L., Lindeman, N. I., Vasalos, P., Shan, M., Jennings, L. J., & Rimm, D. L. (2018). Worldwide Frequency of Commonly Detected EGFR Mutations. Arch Pathol Lab Med, 142(2), 163-167. doi:10.5858/arpa.2016-0579-CP
Gridelli, C., Rossi, A., Ciardiello, F., De Marinis, F., Crinò, L., Morabito, A., . . . Perrone, F. (2016). BEVERLY: Rationale and Design of a Randomized Open-Label Phase III Trial Comparing Bevacizumab Plus Erlotinib Versus Erlotinib Alone as First-Line Treatment of Patients With EGFR-Mutated Advanced Nonsquamous Non-Small-Cell Lung Cancer. Clin Lung Cancer, 17(5), 461-465. doi:10.1016/j.cllc.2016.04.001
Gu, Y., Zhu, X., Cao, B., Wu, X., Tong, X., Shao, Y. W., & Liang, L. (2019). Transformation to small cell lung cancer and activation of KRAS during long-term erlotinib maintenance in a patient with non-small cell lung cancer: A case report. Oncol Lett, 17(6), 5219-5223. doi:10.3892/ol.2019.10196
Guadagno, E., Borrelli, G., Pignatiello, S., Donato, A., Presta, I., Arcidiacono, B., . . . Del Basso De Caro, M. (2019). Anti-Apoptotic and Anti-Oxidant Proteins in Glioblastomas: Immunohistochemical Expression of Beclin and DJ-1 and Its Correlation with Prognosis. Int J Mol Sci, 20(16). doi:10.3390/ijms20164066
Guan, J. L., Zhong, W. Z., An, S. J., Yang, J. J., Su, J., Chen, Z. H., . . . Wu, Y. L. (2013). KRAS mutation in patients with lung cancer: a predictor for poor prognosis but not for EGFR-TKIs or chemotherapy. Ann Surg Oncol, 20(4), 1381-1388. doi:10.1245/s10434-012-2754-z
Guerrera, F., Renaud, S., Tabbó, F., Voegeli, A. C., Filosso, P. L., Legrain, M., . . . Massard, G. (2017). Epidermal growth factor receptor mutations are linked to skip N2 lymph node metastasis in resected non-small-cell lung cancer adenocarcinomas. Eur J Cardiothorac Surg, 51(4), 680-688. doi:10.1093/ejcts/ezw362
Hakozaki, T., & Yomota, M. (2019). Acquisition of T790M resistance mutation in a patient with advanced adenocarcinoma harbouring uncommon EGFR mutations: a case report and literature review. Onco Targets Ther, 12, 745-748. doi:10.2147/ott.S190034
Hamada, K., Tian, Y., Fujimoto, M., Takahashi, Y., Kohno, T., Tsuta, K., . . . Arai, E. (2021). DNA hypermethylation of the ZNF132 gene participates in the clinicopathological aggressiveness of 'pan-negative'-type lung adenocarcinomas. Carcinogenesis, 42(2), 169-179. doi:10.1093/carcin/bgaa115
Hamaguchi, R., Okamoto, T., Sato, M., Hasegawa, M., & Wada, H. (2017). Effects of an Alkaline Diet on EGFR-TKI Therapy in EGFR Mutation-positive NSCLC. Anticancer Res, 37(9), 5141-5145. doi:10.21873/anticanres.11934
Hames, M. L., Chen, H., Iams, W., Aston, J., Lovly, C. M., & Horn, L. (2016). Correlation between KRAS mutation status and response to chemotherapy in patients with advanced non-small cell lung cancer☆. Lung Cancer, 92, 29-34. doi:10.1016/j.lungcan.2015.11.004
Han, B., Tjulandin, S., Hagiwara, K., Normanno, N., Wulandari, L., Laktionov, K., . . . Reck, M. (2017). EGFR mutation prevalence in Asia-Pacific and Russian patients with advanced NSCLC of adenocarcinoma and non-adenocarcinoma histology: The IGNITE study. Lung Cancer, 113, 37-44. doi:10.1016/j.lungcan.2017.08.021
Han, J. Y., Kim, S. H., Lee, Y. S., Lee, S. Y., Hwang, J. A., Kim, J. Y., . . . Lee, G. K. (2014). Comparison of targeted next-generation sequencing with conventional sequencing for predicting the responsiveness to epidermal growth factor receptor-tyrosine kinase inhibitor (EGFR-TKI) therapy in never-smokers with lung adenocarcinoma. Lung Cancer, 85(2), 161-167. doi:10.1016/j.lungcan.2014.04.009
Handorf, E. A., McElligott, S., Vachani, A., Langer, C. J., Bristol Demeter, M., Armstrong, K., & Asch, D. A. (2012). Cost effectiveness of personalized therapy for first-line treatment of stage IV and recurrent incurable adenocarcinoma of the lung. J Oncol Pract, 8(5), 267-274. doi:10.1200/jop.2011.000502
Hashimoto, H., Komori, K., Kameda, K., Taguchi, S., & Ozeki, Y. (2022). Successful salvage surgery followed by second ALK-TKI after alectinib failure in a patient with ALK-positive NSCLC. Surg Case Rep, 8(1), 59. doi:10.1186/s40792-022-01408-7
Hata, Y., Yuasa, R., Sato, F., Otsuka, H., Goto, H., Isobe, K., . . . Watanabe, Y. (2013). Ciliated muconodular papillary tumor of the lung: a newly defined low-grade malignant tumor with CT findings reminiscent of adenocarcinoma. Jpn J Clin Oncol, 43(2), 205-207. doi:10.1093/jjco/hys218
Hattori, A., Matsunaga, T., Fukui, M., Takamochi, K., & Suzuki, K. (2022). Prognosis of epidermal growth factor receptor-mutated stage I lung adenocarcinoma with radiologically solid features. Eur J Cardiothorac Surg, 61(4), 769-777. doi:10.1093/ejcts/ezab481
Hayasaka, K., Shiono, S., Matsumura, Y., Yanagawa, N., Suzuki, H., Abe, J., . . . Okada, Y. (2018). Epidermal Growth Factor Receptor Mutation as a Risk Factor for Recurrence in Lung Adenocarcinoma. Ann Thorac Surg, 105(6), 1648-1654. doi:10.1016/j.athoracsur.2018.01.052
Hayashi, H., Okamoto, I., Kimura, H., Sakai, K., Nishimura, Y., Nishio, K., & Nakagawa, K. (2012). Clinical outcomes of thoracic radiotherapy for locally advanced NSCLC with EGFR mutations or EML4-ALK rearrangement. Anticancer Res, 32(10), 4533-4537. 
He, A., Zhang, R., Wang, J., Huang, Z., Liao, W., Li, Y., . . . Wu, L. (2022). TYK2 is a prognostic biomarker and associated with immune infiltration in the lung adenocarcinoma microenvironment. Asia Pac J Clin Oncol, 18(2), e129-e140. doi:10.1111/ajco.13569
He, L. R., Ma, N. F., Chen, J. W., Li, B. K., Guan, X. Y., Liu, M. Z., & Xie, D. (2015). Overexpression of CHD1L is positively associated with metastasis of lung adenocarcinoma and predicts patients poor survival. Oncotarget, 6(31), 31181-31190. doi:10.18632/oncotarget.5070
He, Y., Yu, H., Rozeboom, L., Rivard, C. J., Ellison, K., Dziadziuszko, R., . . . Hirsch, F. R. (2017). LAG-3 Protein Expression in Non-Small Cell Lung Cancer and Its Relationship with PD-1/PD-L1 and Tumor-Infiltrating Lymphocytes. J Thorac Oncol, 12(5), 814-823. doi:10.1016/j.jtho.2017.01.019
Heist, R. S., Shim, H. S., Gingipally, S., Mino-Kenudson, M., Le, L., Gainor, J. F., . . . Iafrate, A. J. (2016). MET Exon 14 Skipping in Non-Small Cell Lung Cancer. Oncologist, 21(4), 481-486. doi:10.1634/theoncologist.2015-0510
Higuchi, M., Owada, Y., Inoue, T., Watanabe, Y., Yamaura, T., Fukuhara, M., . . . Suzuki, H. (2016). FDG-PET in the evaluation of response to nivolumab in recurrent non-small-cell lung cancer. World J Surg Oncol, 14(1), 238. doi:10.1186/s12957-016-0998-y
Hirai, F., Takenoyama, M., Taguchi, K., Toyozawa, R., Inamasu, E., Toyokawa, G., . . . Ichinose, Y. (2014). Experience with erlotinib in lung adenocarcinoma harboring a coexisting KIF5B-RET fusion gene and EGFR mutation: report of a rare case. J Thorac Oncol, 9(5), e37-39. doi:10.1097/jto.0000000000000097
Hirano, R., Uchino, J., Ueno, M., Fujita, M., & Watanabe, K. (2016). Low-dose Epidermal Growth Factor Receptor (EGFR)- Tyrosine Kinase Inhibition of EGFR Mutation-positive Lung Cancer: Therapeutic Benefits and Associations Between Dosage, Efficacy and Body Surface Area. Asian Pac J Cancer Prev, 17(2), 785-789. doi:10.7314/apjcp.2016.17.2.785
Hochmair, M. J., Kolb, R., Wurm, R., Zach, H., & Bittner, N. (2022). Nintedanib plus Docetaxel after Immune Checkpoint Inhibitor Failure in Patients with Advanced Non-Small-Cell Lung Cancer: A Case Series. Case Rep Oncol, 15(1), 138-148. doi:10.1159/000520939
Honda, K. (2022). Development of Biomarkers to Predict Recurrence by Determining the Metastatic Ability of Cancer Cells. J Nippon Med Sch, 89(1), 24-32. doi:10.1272/jnms.JNMS.2022_89-118
Hsu, Y. C., Chang, Y. H., Chang, G. C., Ho, B. C., Yuan, S. S., Li, Y. C., . . . Chen, H. Y. (2019). Tumor mutation burden and recurrent tumors in hereditary lung cancer. Cancer Med, 8(5), 2179-2187. doi:10.1002/cam4.2120
Hu, C., Shu, L., Chen, C., Fan, S., Liang, Q., Zheng, H., . . . Wu, F. (2022). A prediction model integrated genomic alterations and immune signatures of tumor immune microenvironment for early recurrence of stage I NSCLC after curative resection. Transl Lung Cancer Res, 11(1), 24-42. doi:10.21037/tlcr-21-751
Hu, S. Y., Hsieh, M. S., Hsu, H. H., Tsai, T. M., Chiang, X. H., Tsou, K. C., . . . Chen, J. S. (2018). Correlation of tumor spread through air spaces and clinicopathological characteristics in surgically resected lung adenocarcinomas. Lung Cancer, 126, 189-193. doi:10.1016/j.lungcan.2018.11.003
Hu, X., Han, B., Gu, A., Zhang, Y., Jiao, S. C., Wang, C. L., . . . Sun, Y. (2014). A single-arm, multicenter, safety-monitoring, phase IV study of icotinib in treating advanced non-small cell lung cancer (NSCLC). Lung Cancer, 86(2), 207-212. doi:10.1016/j.lungcan.2014.08.014
Huang, H. J., & Chen, X. Y. (2016). [Fetal adenocarcinoma of the lung: a clinicopathologic analysis of six cases]. Zhonghua Bing Li Xue Za Zhi, 45(9), 617-621. doi:10.3760/cma.j.issn.0529-5807.2016.09.006
Huang, J., Mo, H., Zhang, W., Chen, X., Qu, D., Wang, X., . . . Xu, B. (2019). Promising efficacy of SHR-1210, a novel anti-programmed cell death 1 antibody, in patients with advanced gastric and gastroesophageal junction cancer in China. Cancer, 125(5), 742-749. doi:10.1002/cncr.31855
Huang, X., Yang, Y., Wang, P., Han-Zhang, H., & Ding, L. (2021). A heavily pre-treated adenocarcinoma patient with EGFR exon 20 insertion mutation responded to pembrolizumab plus nab-paclitaxel/bevacizumab: a case report. Ann Palliat Med, 10(6), 6997-7002. doi:10.21037/apm-20-1307
Hulsbergen, A. F. C., Abunimer, A. M., Ida, F., Kavouridis, V. K., Cho, L. D., Tewarie, I. A., . . . Smith, T. R. (2021). Neurosurgical resection for locally recurrent brain metastasis. Neuro Oncol, 23(12), 2085-2094. doi:10.1093/neuonc/noab173
Hwang, D. H., Sholl, L. M., Rojas-Rudilla, V., Hall, D. L., Shivdasani, P., Garcia, E. P., . . . Dong, F. (2016). KRAS and NKX2-1 Mutations in Invasive Mucinous Adenocarcinoma of the Lung. J Thorac Oncol, 11(4), 496-503. doi:10.1016/j.jtho.2016.01.010
Hwang, J. A., Lee, J. Y., Kim, W. S., Song, J. S., Rho, J. K., Choi, C. M., & Lee, J. C. (2016). Clinical Implications of Isolated Bone Failure Without Systemic Disease Progression During EGFR-TKI Treatment. Clin Lung Cancer, 17(6), 573-580.e571. doi:10.1016/j.cllc.2016.05.018
Ichikawa, T., Saruwatari, K., Mimaki, S., Sugano, M., Aokage, K., Kojima, M., . . . Ishii, G. (2017). Immunohistochemical and genetic characteristics of lung cancer mimicking organizing pneumonia. Lung Cancer, 113, 134-139. doi:10.1016/j.lungcan.2017.10.001
Ichiki, Y., Iwanami, T., Kakizoe, K., Hamatsu, T., Suehiro, T., Yoneda, K., . . . Sugimachi, K. (2017). [Analysis of Advanced or Postoperative Recurrent Non-small Lung Cancer Cases Treated with Nivolumab]. J uoeh, 39(4), 291-297. doi:10.7888/juoeh.39.291
Ichiki, Y., Taira, A., Chikaishi, Y., Matsumiya, H., Mori, M., Kanayama, M., . . . Tanaka, F. (2019). Prognostic factors of advanced or postoperative recurrent non-small cell lung cancer targeted with immune check point inhibitors. J Thorac Dis, 11(4), 1117-1123. doi:10.21037/jtd.2019.04.41
Igarashi, Y., Hamabashiri, M., Nishi, M., Sugi, Y., Kakimoto, H., Uchiyama, M., . . . Kamimura, H. (2021). [A Case of Rheumatoid Arthritis Caused by Pembrolizumab Treatment for Non-Small Cell Lung Cancer]. Gan To Kagaku Ryoho, 48(6), 837-839. 
Igawa, S., Sato, Y., Ishihara, M., Kasajima, M., Kusuhara, S., Nakahara, Y., . . . Masuda, N. (2016). EGFR Mutation Genotype Impact on the Efficacy of Pemetrexed in Patients with Nonsquamous Nonsmall Cell Lung Cancer. Asian Pac J Cancer Prev, 17(7), 3249-3253. 
Iijima, Y., Hirotsu, Y., Mochizuki, H., Amemiya, K., Oyama, T., Uchida, Y., . . . Omata, M. (2018). Dynamic Changes and Drug-Induced Selection of Resistant Clones in a Patient With EGFR-Mutated Adenocarcinoma That Acquired T790M Mutation and Transformed to Small-Cell Lung Cancer. Clin Lung Cancer, 19(6), e843-e847. doi:10.1016/j.cllc.2018.07.002
Ikebe, S., Amiya, R., Minami, S., Ihara, S., Higuchi, Y., & Komuta, K. (2021). Osimertinib-induced cardiac failure with QT prolongation and torsade de pointes in a patient with advanced pulmonary adenocarcinoma. Int Cancer Conf J, 10(1), 68-71. doi:10.1007/s13691-020-00450-2
Ikeda, T., Nakamura, Y., Yamaguchi, H., Tomonaga, N., Doi, S., Nakatomi, K., . . . Kohno, S. (2012). Direct comparison of 3 PCR methods in detecting EGFR mutations in patients with advanced non-small-cell lung cancer. Clin Lung Cancer, 13(5), 369-374. doi:10.1016/j.cllc.2012.01.008
Imielinski, M., Berger, A. H., Hammerman, P. S., Hernandez, B., Pugh, T. J., Hodis, E., . . . Meyerson, M. (2012). Mapping the hallmarks of lung adenocarcinoma with massively parallel sequencing. Cell, 150(6), 1107-1120. doi:10.1016/j.cell.2012.08.029
Inoue, Y., Inui, N., Asada, K., Karayama, M., Matsuda, H., Yokomura, K., . . . Suda, T. (2015). Phase II study of erlotinib in elderly patients with non-small cell lung cancer harboring epidermal growth factor receptor mutations. Cancer Chemother Pharmacol, 76(1), 155-161. doi:10.1007/s00280-015-2784-x
Isaka, T., Nakayama, H., Ito, H., Yokose, T., Yamada, K., & Masuda, M. (2018). Impact of the epidermal growth factor receptor mutation status on the prognosis of recurrent adenocarcinoma of the lung after curative surgery. BMC Cancer, 18(1), 959. doi:10.1186/s12885-018-4849-9
Isaksson, S., George, A. M., Jönsson, M., Cirenajwis, H., Jönsson, P., Bendahl, P. O., . . . Planck, M. (2019). Pre-operative plasma cell-free circulating tumor DNA and serum protein tumor markers as predictors of lung adenocarcinoma recurrence. Acta Oncol, 58(8), 1079-1086. doi:10.1080/0284186x.2019.1610573
Ishida, H., Shimizu, Y., Sakaguchi, H., Nitanda, H., Kaneko, K., Yamazaki, N., . . . Kobayashi, K. (2019). Distinctive clinicopathological features of adenocarcinoma in situ and minimally invasive adenocarcinoma of the lung: A retrospective study. Lung Cancer, 129, 16-21. doi:10.1016/j.lungcan.2018.12.020
Ishii, H., Azuma, K., Kawahara, A., Kinoshita, T., Matsuo, N., Naito, Y., . . . Hoshino, T. (2020). Predictive value of CD73 expression for the efficacy of immune checkpoint inhibitors in NSCLC. Thorac Cancer, 11(4), 950-955. doi:10.1111/1759-7714.13346
Isobe, K., Kakimoto, A., Mikami, T., Kaburaki, K., Kobayashi, H., Yoshizawa, T., . . . Homma, S. (2018). PD-L1 mRNA expression in EGFR-mutant lung adenocarcinoma. Oncol Rep, 40(1), 331-338. doi:10.3892/or.2018.6442
Ito, M., Codony-Servat, J., Giménez-Capitán, A., Serra-Mitjans, M., Pérez-Ochoa, F., Llige, D., . . . Rosell, R. (2020). Src-Homology 2 Domain-Containing Phosphatase 2 in Resected EGFR Mutation-Positive Lung Adenocarcinoma. JTO Clin Res Rep, 1(4), 100084. doi:10.1016/j.jtocrr.2020.100084
Ito, M., Miyata, Y., Hirano, S., Kimura, S., Irisuna, F., Ikeda, K., . . . Okada, M. (2019). Synchronicity of genetic variants between primary sites and metastatic lymph nodes, and prognostic impact in nodal metastatic lung adenocarcinoma. J Cancer Res Clin Oncol, 145(9), 2325-2333. doi:10.1007/s00432-019-02978-0
Ito, M., Miyata, Y., Kushitani, K., Yoshiya, T., Kai, Y., Tsutani, Y., . . . Okada, M. (2018). Increased risk of recurrence in resected EGFR-positive pN0M0 invasive lung adenocarcinoma. Thorac Cancer, 9(12), 1594-1602. doi:10.1111/1759-7714.12866
Ito, M., Miyata, Y., Tsutani, Y., Ito, H., Nakayama, H., Imai, K., . . . Okada, M. (2020). Positive EGFR mutation status is a risk of recurrence in pN0-1 lung adenocarcinoma when combined with pathological stage and histological subtype: A retrospective multi-center analysis. Lung Cancer, 141, 107-113. doi:10.1016/j.lungcan.2020.01.018
Izar, B., Zhou, H., Heist, R. S., Azzoli, C. G., Muzikansky, A., Scribner, E. E., . . . Lanuti, M. (2014). The prognostic impact of KRAS, its codon and amino acid specific mutations, on survival in resected stage I lung adenocarcinoma. J Thorac Oncol, 9(9), 1363-1369. doi:10.1097/jto.0000000000000266
Jabbour, S. K., Kim, S., Haider, S. A., Xu, X., Wu, A., Surakanti, S., . . . Zou, W. (2015). Reduction in Tumor Volume by Cone Beam Computed Tomography Predicts Overall Survival in Non-Small Cell Lung Cancer Treated With Chemoradiation Therapy. Int J Radiat Oncol Biol Phys, 92(3), 627-633. doi:10.1016/j.ijrobp.2015.02.017
Jakubek, Y., Lang, W., Vattathil, S., Garcia, M., Xu, L., Huang, L., . . . Kadara, H. (2016). Genomic Landscape Established by Allelic Imbalance in the Cancerization Field of a Normal Appearing Airway. Cancer Res, 76(13), 3676-3683. doi:10.1158/0008-5472.Can-15-3064
Jeon, J. H., Kang, C. H., Kim, H. S., Seong, Y. W., Park, I. K., & Kim, Y. T. (2015). Prognostic and predictive role of epidermal growth factor receptor mutation in recurrent pulmonary adenocarcinoma after curative resection. Eur J Cardiothorac Surg, 47(3), 556-562. doi:10.1093/ejcts/ezu177
Jia, M., Yu, S., Cao, L., Sun, P. L., & Gao, H. (2020). Clinicopathologic Features and Genetic Alterations in Adenocarcinoma In Situ and Minimally Invasive Adenocarcinoma of the Lung: Long-Term Follow-Up Study of 121 Asian Patients. Ann Surg Oncol, 27(8), 3052-3063. doi:10.1245/s10434-020-08241-y
Jia, P., & Zhao, Z. (2014). VarWalker: personalized mutation network analysis of putative cancer genes from next-generation sequencing data. PLoS Comput Biol, 10(2), e1003460. doi:10.1371/journal.pcbi.1003460
Jia, X., He, Q., Xing, X., Yang, Y., & Ma, Y. (2022). A Novel LOC101927967 Intergenic Region ALK Fusion Identified by NGS and Validated by IHC and FISH in a Patient with Early Stage Adenocarcinoma of Lung. Onco Targets Ther, 15, 251-254. doi:10.2147/ott.S347200
Jia, Z., Xing, J., Li, J., Wang, W., Wang, Y., Song, Y., . . . Li, S. (2021). HER2 transmembrane domain mutation: comprehensive characteristics and real-world evidence of treatment response in Chinese lung adenocarcinoma. Transl Lung Cancer Res, 10(3), 1383-1396. doi:10.21037/tlcr-21-107
Jiang, Y., Liu, X., Lv, D. L., & Zhao, X. L. (2022). Kirsten rat sarcoma viral oncogene homolog G12C mutant advanced non-small-cell lung cancer treated with MEK1/2 inhibitor trametinib: a case report. Anticancer Drugs, 33(1), e752-e755. doi:10.1097/cad.0000000000001176
Jiang, Y., Shou, L., Guo, Q., Bao, Y., Xu, X., An, S., & Lu, J. (2021). Small-cell lung cancer transformation from EGFR-mutant adenocarcinoma after EGFR-TKIs resistance: A case report. Medicine (Baltimore), 100(32), e26911. doi:10.1097/md.0000000000026911
Jin, Y., Chen, M., & Yu, X. (2016). Differences among lesions with exon 19, exon 21 EGFR mutations and wild types in surgically resected non-small cell lung cancer. Sci Rep, 6, 31636. doi:10.1038/srep31636
Jing, W., Ma, J. T., & Han, C. B. (2020). Metastatic Breast Cancer Coexisting With HER-2 Amplification and EGFR Exon 19 Deletion Benefits From EGFR-TKI Therapy: A Case Report. Front Oncol, 10, 771. doi:10.3389/fonc.2020.00771
Jones, G. D., Caso, R., Tan, K. S., Mastrogiacomo, B., Sanchez-Vega, F., Liu, Y., . . . Isbell, J. M. (2021). KRAS (G12C) Mutation Is Associated with Increased Risk of Recurrence in Surgically Resected Lung Adenocarcinoma. Clin Cancer Res, 27(9), 2604-2612. doi:10.1158/1078-0432.Ccr-20-4772
Ju, Q., Huang, T., Zhang, Y., Wu, L., Geng, J., Mu, X., . . . Zhang, J. (2021). Systemic immune-inflammation index predicts prognosis in patients with different EGFR-mutant lung adenocarcinoma. Medicine (Baltimore), 100(6), e24640. doi:10.1097/md.0000000000024640
Kachala, S. S., Bograd, A. J., Villena-Vargas, J., Suzuki, K., Servais, E. L., Kadota, K., . . . Adusumilli, P. S. (2014). Mesothelin overexpression is a marker of tumor aggressiveness and is associated with reduced recurrence-free and overall survival in early-stage lung adenocarcinoma. Clin Cancer Res, 20(4), 1020-1028. doi:10.1158/1078-0432.Ccr-13-1862
Kadara, H., Choi, M., Zhang, J., Parra, E. R., Rodriguez-Canales, J., Gaffney, S. G., . . . Herbst, R. S. (2017). Whole-exome sequencing and immune profiling of early-stage lung adenocarcinoma with fully annotated clinical follow-up. Ann Oncol, 28(1), 75-82. doi:10.1093/annonc/mdw436
Kadota, K., Sima, C. S., Arcila, M. E., Hedvat, C., Kris, M. G., Jones, D. R., . . . Travis, W. D. (2016). KRAS Mutation Is a Significant Prognostic Factor in Early-stage Lung Adenocarcinoma. Am J Surg Pathol, 40(12), 1579-1590. doi:10.1097/pas.0000000000000744
Kaira, K., Yamamoto, N., Kenmotsu, H., Murakami, H., Ono, A., Naito, T., . . . Takahashi, T. (2014). Prognostic impact of 18F-FDG uptake on PET in non-small cell lung cancer patients with postoperative recurrence following platinum-based chemotherapy. Respir Investig, 52(2), 121-128. doi:10.1016/j.resinv.2013.08.008
Kakimi, K., Matsushita, H., Masuzawa, K., Karasaki, T., Kobayashi, Y., Nagaoka, K., . . . Nakajima, J. (2020). Adoptive transfer of zoledronate-expanded autologous Vγ9Vδ2 T-cells in patients with treatment-refractory non-small-cell lung cancer: a multicenter, open-label, single-arm, phase 2 study. J Immunother Cancer, 8(2). doi:10.1136/jitc-2020-001185
Kamiyoshihara, M., Igai, H., Ibe, T., Ohsawa, F., Yoshikawa, R., Shimizu, K., . . . Kuwano, H. (2018). [Multidisciplinary Approach to Recurrence after Resection of Primary Lung Cancer]. Kyobu Geka, 71(4), 302-310. 
Kamiyoshihara, M., Igai, H., Matsuura, N., Yazawa, T., & Ohsawa, F. (2021). [Alectinib for an Octogenarian Patient with Poor Performance Status and ALK Fusion Gene-Positive Lung Cancer-A Case Report]. Gan To Kagaku Ryoho, 48(8), 1053-1055. 
Kaneda, T., Yoshioka, H., Tamiya, M., Tamiya, A., Hata, A., Okada, A., . . . Katakami, N. (2018). Differential efficacy of cisplatin plus pemetrexed between L858R and Del-19 in advanced EGFR-mutant non-squamous non-small cell lung cancer. BMC Cancer, 18(1), 6. doi:10.1186/s12885-017-3952-7
Karachaliou, N., Sosa, A. E., Barron, F. B., Gonzalez Cao, M., Santarpia, M., & Rosell, R. (2017). Pharmacological management of relapsed/refractory NSCLC with chemical drugs. Expert Opin Pharmacother, 18(3), 295-304. doi:10.1080/14656566.2017.1285284
Kaseda, K., Asakura, K., Kazama, A., & Ozawa, Y. (2017). Clinicopathological and prognostic features of surgically resected pathological stage I lung adenocarcinoma harboring epidermal growth factor receptor and K-ras mutation. Thorac Cancer, 8(3), 229-237. doi:10.1111/1759-7714.12428
Katakura, S., Kobayashi, N., Somekawa, K., Masumoto, N., Kudo, M., & Kaneko, T. (2019). Non-small cell lung cancer with mesenchymal-epithelial transition gene exon 14 skipping mutation treated with crizotinib. Respirol Case Rep, 7(7), e00453. doi:10.1002/rcr2.453
Katano, T., Oda, T., Sekine, A., Sato, M., Yamaya, T., Sato, Y., . . . Ogura, T. (2020). Five cases of BRAF V600E-mutant lung adenocarcinoma with high expression of programmed death ligand 1. Respir Med Case Rep, 30, 101071. doi:10.1016/j.rmcr.2020.101071
Kato, T., Koriyama, C., Khan, N., Samukawa, T., Yanagi, M., Hamada, T., . . . Akiba, S. (2012). EGFR mutations and human papillomavirus in lung cancer. Lung Cancer, 78(2), 144-147. doi:10.1016/j.lungcan.2012.08.011
Kato, Y., Kato, Y., Minegishi, Y., Suzuki, T., Nakamichi, S., Matsumoto, M., . . . Gemma, A. (2021). Efficacy with Trastuzumab Deruxtecan for Non-Small-Cell Lung Cancer Harboring HER2 Exon 20 Insertion Mutation in a Patient with a Poor Performance Status: A Case Report. Onco Targets Ther, 14, 5315-5319. doi:10.2147/ott.S341290
Katono, K., Kasajima, M., Ishihara, M., Hayashi, N., Nagashima, Y., Igawa, S., & Masuda, N. (2013). [A case of lung adenocarcinoma with coexisting G719X and T790M EGFR mutations in which erlotinib was effective for the treatment of leptomeningeal carcinomatosis]. Gan To Kagaku Ryoho, 40(3), 375-377. 
Katzendorn, O., Peters, I., Dubrowinskaja, N., Tezval, H., Tabrizi, P. F., von Klot, C. A., . . . Serth, J. (2021). DNA methylation of tumor associated calcium signal transducer 2 (TACSTD2) loci shows association with clinically aggressive renal cell cancers. BMC Cancer, 21(1), 444. doi:10.1186/s12885-021-08172-1
Kawaguchi, Y., Hanaoka, J., Hayashi, H., Mizusaki, N., Iihara, H., Itoh, Y., & Sugiyama, T. (2017). Clinical Efficacy of Afatinib Treatment for a Patient with Leptomeningeal Carcinomatosis. Chemotherapy, 62(3), 147-150. doi:10.1159/000454727
Kawai, H., Iguchi, K., Takayashiki, N., Okauchi, S., & Satoh, H. (2020). Metachronous Isolated Contralateral Lung Metastasis from Pulmonary Adenosquamous Carcinoma with EGFR Mutation. Acta Medica (Hradec Kralove), 63(3), 141-144. doi:10.14712/18059694.2020.33
Kazdal, D., Harms, A., Endris, V., Penzel, R., Kriegsmann, M., Eichhorn, F., . . . Warth, A. (2017). Prevalence of somatic mitochondrial mutations and spatial distribution of mitochondria in non-small cell lung cancer. Br J Cancer, 117(2), 220-226. doi:10.1038/bjc.2017.155
Khosravi, N., Caetano, M. S., Cumpian, A. M., Unver, N., De la Garza Ramos, C., Noble, O., . . . Moghaddam, S. J. (2018). IL22 Promotes Kras-Mutant Lung Cancer by Induction of a Protumor Immune Response and Protection of Stemness Properties. Cancer Immunol Res, 6(7), 788-797. doi:10.1158/2326-6066.Cir-17-0655
Kim, D., Lee, Y. S., Kim, D. H., & Bae, S. C. (2020). Lung Cancer Staging and Associated Genetic and Epigenetic Events. Mol Cells, 43(1), 1-9. doi:10.14348/molcells.2020.2246
Kim, H. C., Kang, Y. R., Ji, W., Kim, Y. J., Yoon, S., Lee, J. C., & Choi, C. M. (2019). Frequency and clinical features of BRAF mutations among patients with stage III/IV lung adenocarcinoma without EGFR/ALK aberrations. Onco Targets Ther, 12, 6045-6052. doi:10.2147/ott.S213928
Kim, H. J., Lee, K. Y., Kim, Y. C., Kim, K. S., Lee, S. Y., Jang, T. W., . . . Kim, S. Y. (2012). Detection and comparison of peptide nucleic acid-mediated real-time polymerase chain reaction clamping and direct gene sequencing for epidermal growth factor receptor mutations in patients with non-small cell lung cancer. Lung Cancer, 75(3), 321-325. doi:10.1016/j.lungcan.2011.08.005
Kim, I. A., Hur, J. Y., Kim, H. J., Lee, S. A., Hwang, J. J., Kim, W. S., & Lee, K. Y. (2021). Targeted Next-Generation Sequencing Analysis Predicts the Recurrence in Resected Lung Adenocarcinoma Harboring EGFR Mutations. Cancers (Basel), 13(14). doi:10.3390/cancers13143632
Kim, I. A., Hur, J. Y., Kim, H. J., Park, J. H., Hwang, J. J., Lee, S. A., . . . Lee, K. Y. (2021). Targeted Next-Generation Sequencing Analysis for Recurrence in Early-Stage Lung Adenocarcinoma. Ann Surg Oncol, 28(7), 3983-3993. doi:10.1245/s10434-020-09276-x
Kim, I. A., Lee, J. S., Kim, H. J., Kim, W. S., & Lee, K. Y. (2018). Cumulative smoking dose affects the clinical outcomes of EGFR-mutated lung adenocarcinoma patients treated with EGFR-TKIs: a retrospective study. BMC Cancer, 18(1), 768. doi:10.1186/s12885-018-4691-0
Kim, J. E., Kim, H., Choe, J. Y., Sun, P., Jheon, S., & Chung, J. H. (2013). High expression of Sonic hedgehog signaling proteins is related to the favorable outcome, EGFR mutation, and lepidic predominant subtype in primary lung adenocarcinoma. Ann Surg Oncol, 20 Suppl 3, S570-576. doi:10.1245/s10434-013-3022-6
Kim, L., Kim, Y. S., Lee, J. S., Choi, S. J., Park, I. S., Han, J. Y., . . . Chu, Y. C. (2017). Ciliated muconodular papillary tumor of the lung harboring BRAF V600E mutation and p16(INK4a) overexpression without proliferative activity may represent an example of oncogene-induced senescence. J Thorac Dis, 9(12), E1039-e1044. doi:10.21037/jtd.2017.11.120
Kim, M. H., Kim, H. R., Cho, B. C., Bae, M. K., Kim, E. Y., Lee, C. Y., . . . Kim, J. H. (2014). Impact of cigarette smoking on response to epidermal growth factor receptor (EGFR)-tyrosine kinase inhibitors in lung adenocarcinoma with activating EGFR mutations. Lung Cancer, 84(2), 196-202. doi:10.1016/j.lungcan.2014.01.022
Kim, M. H., Shim, H. S., Kang, D. R., Jung, J. Y., Lee, C. Y., Kim, D. J., . . . Cho, B. C. (2014). Clinical and prognostic implications of ALK and ROS1 rearrangements in never-smokers with surgically resected lung adenocarcinoma. Lung Cancer, 83(3), 389-395. doi:10.1016/j.lungcan.2014.01.003
Kim, M. S., Chung, N. G., Kim, M. S., Yoo, N. J., & Lee, S. H. (2013). Somatic mutation of IL7R exon 6 in acute leukemias and solid cancers. Hum Pathol, 44(4), 551-555. doi:10.1016/j.humpath.2012.06.017
Kim, Y., Hammerman, P. S., Kim, J., Yoon, J. A., Lee, Y., Sun, J. M., . . . Park, K. (2014). Integrative and comparative genomic analysis of lung squamous cell carcinomas in East Asian patients. J Clin Oncol, 32(2), 121-128. doi:10.1200/jco.2013.50.8556
Kim, Y. I., Paeng, J. C., Park, Y. S., Cheon, G. J., Lee, D. S., Chung, J. K., & Kang, K. W. (2018). Relation of EGFR Mutation Status to Metabolic Activity in Localized Lung Adenocarcinoma and Its Influence on the Use of FDG PET/CT Parameters in Prognosis. AJR Am J Roentgenol, 210(6), 1346-1351. doi:10.2214/ajr.17.18916
Kim, Y. T., Seong, Y. W., Jung, Y. J., Jeon, Y. K., Park, I. K., Kang, C. H., & Kim, J. H. (2013). The presence of mutations in epidermal growth factor receptor gene is not a prognostic factor for long-term outcome after surgical resection of non-small-cell lung cancer. J Thorac Oncol, 8(2), 171-178. doi:10.1097/JTO.0b013e318277a3bb
Kimura, T., Nakamura, H., Omura, A., Ike, A., Hiroshima, T., Maniwa, T., . . . Okami, J. (2020). Novel imprint cytological classification is correlated with tumor spread through air spaces in lung adenocarcinoma. Lung Cancer, 148, 62-68. doi:10.1016/j.lungcan.2020.08.005
Kirienko, M., Sollini, M., Corbetta, M., Voulaz, E., Gozzi, N., Interlenghi, M., . . . Chiti, A. (2021). Radiomics and gene expression profile to characterise the disease and predict outcome in patients with lung cancer. Eur J Nucl Med Mol Imaging, 48(11), 3643-3655. doi:10.1007/s00259-021-05371-7
Kiriu, T., Tamura, D., Tachihara, M., Sekiya, R., Hazama, D., Katsurada, M., . . . Nishimura, Y. (2018). Successful Osimertinib Rechallenge with Steroid Therapy after Osimertinib-induced Interstitial Lung Disease. Intern Med, 57(1), 91-95. doi:10.2169/internalmedicine.8947-17
Kishi, N., Ito, M., Miyata, Y., Kanai, A., Handa, Y., Tsutani, Y., . . . Okada, M. (2020). Intense Expression of EGFR L858R Characterizes the Micropapillary Component and L858R Is Associated with the Risk of Recurrence in pN0M0 Lung Adenocarcinoma with the Micropapillary Component. Ann Surg Oncol, 27(3), 945-955. doi:10.1245/s10434-019-07854-2
Kishikawa, T., Kasai, T., Okada, M., Nakachi, I., Soda, S., Arai, R., . . . Sata, M. (2020). Osimertinib, a third-generation EGFR tyrosine kinase inhibitor: A retrospective multicenter study of its real-world efficacy and safety in advanced/recurrent non-small cell lung carcinoma. Thorac Cancer, 11(4), 935-942. doi:10.1111/1759-7714.13378
Kitade, H., Yamada, T., Igarashi, S., Hokkoku, K., Mori, M., Shintaku, K., . . . Yano, S. (2013). [Efficacy of low-dose erlotinib against gefitinib-induced hepatotoxicity in a patient with lung adenocarcinoma harboring EGFR mutations]. Gan To Kagaku Ryoho, 40(1), 79-81. 
Kitahara, H., Okamoto, T., Shimamatsu, S., Kohno, M., Morodomi, Y., Tagawa, T., . . . Mori, M. (2020). LINE-1 Hypomethylation Is Associated With Malignant Traits and Cell Proliferation in Lung Adenocarcinoma. Anticancer Res, 40(10), 5659-5666. doi:10.21873/anticanres.14579
Kneuertz, P. J., Carbone, D. P., D'Souza, D. M., Shilo, K., Abdel-Rasoul, M., Zhao, W., . . . Merritt, R. E. (2020). Prognostic value and therapeutic implications of expanded molecular testing for resected early stage lung adenocarcinoma. Lung Cancer, 143, 60-66. doi:10.1016/j.lungcan.2020.03.012
Ko, E., Lee, B. B., Kim, Y., Lee, E. J., Cho, E. Y., Han, J., . . . Kim, D. H. (2013). Association of RASSF1A and p63 with poor recurrence-free survival in node-negative stage I-II non-small cell lung cancer. Clin Cancer Res, 19(5), 1204-1212. doi:10.1158/1078-0432.Ccr-12-2848
Ko, J. H., Gu, W., Lim, I., Bang, H., Ko, E. A., & Zhou, T. (2014). Ion channel gene expression in lung adenocarcinoma: potential role in prognosis and diagnosis. PLoS One, 9(1), e86569. doi:10.1371/journal.pone.0086569
Kobayashi, H., Wakuda, K., & Takahashi, T. (2016). Effectiveness of afatinib in lung cancer with paralytic ileus due to peritoneal carcinomatosis. Respirol Case Rep, 4(6), e00197. doi:10.1002/rcr2.197
Kobayashi, K., Kaira, K., Iemura, H., Shinomiya, S., Hashimoto, K., Miura, Y., . . . Kagamu, H. (2021). Combination of immune check inhibitor and immunomodulatory arabinomannan extracted from Mycobacterium tuberculosis: A case report. Mol Clin Oncol, 15(5), 227. doi:10.3892/mco.2021.2390
Kobayashi, K., Naoki, K., Kuroda, A., Yasuda, H., Kawada, I., Soejima, K., & Betsuyaku, T. (2018). EGFR-mutant Non-small Cell Lung Cancer Accompanied by Transient Asymptomatic Pulmonary Opacities Successfully Treated with "Stop-And-Go" Osimertinib. Intern Med, 57(7), 1007-1010. doi:10.2169/internalmedicine.9609-17
Kohno, M., Okamoto, T., Suda, K., Shimokawa, M., Kitahara, H., Shimamatsu, S., . . . Maehara, Y. (2014). Prognostic and therapeutic implications of aromatase expression in lung adenocarcinomas with EGFR mutations. Clin Cancer Res, 20(13), 3613-3622. doi:10.1158/1078-0432.Ccr-13-2683
Kondo, N., Takuwa, T., Hashimoto, M., Yoneda, K., Matsumoto, S., Shiraishi, T., & Hasegawa, S. (2015). Gene Mutation Analysis in Determining Late Recurrence of Adenocarcinoma of the Lung. Ann Thorac Surg, 100(2), 711-713. doi:10.1016/j.athoracsur.2014.09.074
Kondo, Y., Ichinose, J., Ninomiya, H., Hashimoto, K., Matsuura, Y., Nakao, M., . . . Mun, M. (2022). Combination of epidermal growth factor receptor mutation and the presence of high-grade patterns is associated with recurrence in resected stage I lung adenocarcinoma. Interact Cardiovasc Thorac Surg. doi:10.1093/icvts/ivac062
Kosaka, T., Yamaki, E., Mogi, A., & Kuwano, H. (2014). A case of lung adenocarcinoma with postoperative recurrence of multiple bone metastases that showed a gradual complete response to combined administration of erlotinib and zoledronic acid. Tumori, 100(2), e45-48. doi:10.1700/1491.16433
Kowalczuk, O., Kozlowski, M., Milewski, R., Minarowski, L., & Reszec, J. (2015). Significance of EGFR signaling pathway genetic alterations in radically resected non-small cell lung cancers from a Polish cohort. One institutional study. Adv Med Sci, 60(2), 277-286. doi:10.1016/j.advms.2015.05.004
Koyama, N., & Uchida, Y. (2013). Clinical significance of erlotinib monotherapy for gefitinib-resistant non-small cell lung cancer with EGFR mutations. Anticancer Res, 33(11), 5083-5089. 
Kratz, J. R., Li, J. Z., Tsui, J., Lee, J. C., Ding, V. W., Rao, A. A., . . . Jablons, D. M. (2021). Genetic and immunologic features of recurrent stage I lung adenocarcinoma. Sci Rep, 11(1), 23690. doi:10.1038/s41598-021-02946-0
Kriegsmann, M., Harms, A., Kazdal, D., Fischer, S., Stenzinger, A., Leichsenring, J., . . . Warth, A. (2018). Analysis of the proliferative activity in lung adenocarcinomas with specific driver mutations. Pathol Res Pract, 214(3), 408-416. doi:10.1016/j.prp.2017.12.018
Kristiansen, C., Olsen, K. E., Hansen, N. C., Hansen, K. H., & Hansen, O. (2012). Epidermal growth factor receptor mutations in synchronous recurrent lung cancer in an 82-year-old woman. A case story. Acta Oncol, 51(7), 948-949. doi:10.3109/0284186x.2011.652739
Kuang, M., Shen, X., Yuan, C., Hu, H., Zhang, Y., Pan, Y., . . . Sun, Y. (2018). Clinical Significance of Complex Glandular Patterns in Lung Adenocarcinoma: Clinicopathologic and Molecular Study in a Large Series of Cases. Am J Clin Pathol, 150(1), 65-73. doi:10.1093/ajcp/aqy032
Kudo, Y., Shimada, Y., Saji, H., Kato, Y., Yoshida, K., Matsubayashi, J., . . . Ikeda, N. (2015). Prognostic Factors for Survival After Recurrence in Patients With Completely Resected Lung Adenocarcinoma: Important Roles of Epidermal Growth Factor Receptor Mutation Status and the Current Staging System. Clin Lung Cancer, 16(6), e213-221. doi:10.1016/j.cllc.2015.04.005
Kunimasa, K., Nishino, K., Kukita, Y., Matsumoto, S., Kawachi, H., Kawamura, T., . . . Kumagai, T. (2021). Late recurrence of lung adenocarcinoma harboring EGFR exon 20 insertion (A763_Y764insFQEA) mutation successfully treated with osimertinib. Cancer Genet, 256-257, 57-61. doi:10.1016/j.cancergen.2021.04.001
Kunimasa, K., Okami, J., Takenaka, S., Honma, K., Kukita, Y., Nagata, S., . . . Kumagai, T. (2021). Conversion Surgery for Advanced Thoracic SMARCA4-Deficient Undifferentiated Tumor With Atezolizumab in Combination With Bevacizumab, Paclitaxel, and Carboplatin Treatment: A Case Report. JTO Clin Res Rep, 2(11), 100235. doi:10.1016/j.jtocrr.2021.100235
Kunimasa, K., Tachihara, M., Terashita, T., & Nishimura, Y. (2015). Gradually Enlarging Crazy-paving Appearance. Intern Med, 54(21), 2791-2792. doi:10.2169/internalmedicine.54.5127
Lacour, M., Hiltbrunner, S., Lee, S. Y., Soltermann, A., Rushing, E. J., Soldini, D., . . . Curioni-Fontecedro, A. (2019). Adjuvant Chemotherapy Increases Programmed Death-Ligand 1 (PD-L1) Expression in Non-small Cell Lung Cancer Recurrence. Clin Lung Cancer, 20(5), 391-396. doi:10.1016/j.cllc.2019.05.013
Laderian, B., Mundi, P., Fojo, T., & S, E. Bates. (2020). Emerging Therapeutic Implications of STK11 Mutation: Case Series. Oncologist, 25(9), 733-737. doi:10.1634/theoncologist.2019-0846
Lai, L., Meng, W., Wei, J., Zhang, X., Tan, Z., Lu, Y., & Hou, E. (2021). Transformation of NSCLC to SCLC after 1st- and 3rd-generation EGFR-TKI resistance and response to EP regimen and erlotinib: 2 CARE-compliant case reports. Medicine (Baltimore), 100(10), e25046. doi:10.1097/md.0000000000025046
Lai, W. V., Lebas, L., Barnes, T. A., Milia, J., Ni, A., Gautschi, O., . . . Li, B. T. (2019). Afatinib in patients with metastatic or recurrent HER2-mutant lung cancers: a retrospective international multicentre study. Eur J Cancer, 109, 28-35. doi:10.1016/j.ejca.2018.11.030
Lan, Y., Zhou, S., Feng, W., Qiao, Y., Du, X., & Li, F. (2021). Association of tumor mutation burden and epidermal growth factor receptor inhibitor history with survival in patients with metastatic stage III/IV non-small-cell lung cancer: A retrospective study. Clinics (Sao Paulo), 76, e2251. doi:10.6061/clinics/2021/e2251
Laurie, S. A., Goss, G. D., Shepherd, F. A., Reaume, M. N., Nicholas, G., Philip, L., . . . Leighl, N. B. (2014). A phase II trial of saracatinib, an inhibitor of src kinases, in previously-treated advanced non-small-cell lung cancer: the princess margaret hospital phase II consortium. Clin Lung Cancer, 15(1), 52-57. doi:10.1016/j.cllc.2013.08.001
Lazar, V., Suo, C., Orear, C., van den Oord, J., Balogh, Z., Guegan, J., . . . Pawitan, Y. (2013). Integrated molecular portrait of non-small cell lung cancers. BMC Med Genomics, 6, 53. doi:10.1186/1755-8794-6-53
Lee, G. D., Lee, S. E., Oh, D. Y., Yu, D. B., Jeong, H. M., Kim, J., . . . Kim, H. R. (2017). MET Exon 14 Skipping Mutations in Lung Adenocarcinoma: Clinicopathologic Implications and Prognostic Values. J Thorac Oncol, 12(8), 1233-1246. doi:10.1016/j.jtho.2017.04.031
Lee, J., Lee, S. E., Kang, S. Y., Do, I. G., Lee, S., Ha, S. Y., . . . Kim, K. M. (2013). Identification of ROS1 rearrangement in gastric adenocarcinoma. Cancer, 119(9), 1627-1635. doi:10.1002/cncr.27967
Lee, J. M., Kim, H. G., Shin, S. Y., & Lee, S. H. (2021). Clinical application of next-generation sequencing for the management of desmoid tumors: A case report and literature review. Medicine (Baltimore), 100(1), e24238. doi:10.1097/md.0000000000024238
Lee, J. S., Kim, H. R., Lee, C. Y., Shin, M., & Shim, H. S. (2013). EGFR and TTF-1 gene amplification in surgically resected lung adenocarcinomas: clinicopathologic significance and effect on response to EGFR-tyrosine kinase inhibitors in recurred cases. Ann Surg Oncol, 20(9), 3015-3022. doi:10.1245/s10434-013-2937-2
Lee, J. Y., Ku, B. M., Lim, S. H., Lee, M. Y., Kim, H., Kim, M., . . . Ahn, M. J. (2015). The BIM Deletion Polymorphism and its Clinical Implication in Patients with EGFR-Mutant Non-Small-Cell Lung Cancer Treated with EGFR Tyrosine Kinase Inhibitors. J Thorac Oncol, 10(6), 903-909. doi:10.1097/jto.0000000000000535
Lee, K., Kim, H. R., Kim, D. K., Kim, Y. H., Park, S. I., Choi, S. H., & Han, J. (2017). Post-recurrence survival analysis of stage I non-small-cell lung cancer. Asian Cardiovasc Thorac Ann, 25(9), 623-629. doi:10.1177/0218492317737641
Lee, S. H., Lee, J. K., Ahn, M. J., Kim, D. W., Sun, J. M., Keam, B., . . . Park, K. (2017). Vandetanib in pretreated patients with advanced non-small cell lung cancer-harboring RET rearrangement: a phase II clinical trial. Ann Oncol, 28(2), 292-297. doi:10.1093/annonc/mdw559
Lee, T. F., Tseng, Y. C., Nguyen, P. A., Li, Y. C., Ho, C. C., & Wu, C. W. (2018). Enhanced YAP expression leads to EGFR TKI resistance in lung adenocarcinomas. Sci Rep, 8(1), 271. doi:10.1038/s41598-017-18527-z
Lee, V. H., Tin, V. P., Choy, T. S., Lam, K. O., Choi, C. W., Chung, L. P., . . . Wong, M. P. (2013). Association of exon 19 and 21 EGFR mutation patterns with treatment outcome after first-line tyrosine kinase inhibitor in metastatic non-small-cell lung cancer. J Thorac Oncol, 8(9), 1148-1155. doi:10.1097/JTO.0b013e31829f684a
Lee, Y., Jeon, J. H., Goh, S. H., Roh, H., Yun, J. Y., Kwon, N. J., . . . Han, J. Y. (2019). The clinical impact of family history of cancer in female never-smoker lung adenocarcinoma. Lung Cancer, 136, 15-22. doi:10.1016/j.lungcan.2019.07.031
Lengel, H. B., Connolly, J. G., Jones, G. D., Caso, R., Zhou, J., Sanchez-Vega, F., . . . Jones, D. R. (2021). The Emerging Importance of Tumor Genomics in Operable Non-Small Cell Lung Cancer. Cancers (Basel), 13(15). doi:10.3390/cancers13153656
Li, B., Cui, Y., Diehn, M., & Li, R. (2017). Development and Validation of an Individualized Immune Prognostic Signature in Early-Stage Nonsquamous Non-Small Cell Lung Cancer. JAMA Oncol, 3(11), 1529-1537. doi:10.1001/jamaoncol.2017.1609
Li, C., Shen, Y., Hu, F., Chu, T., Yang, X., Shao, J., . . . Zhang, X. (2020). Micropapillary pattern is associated with the development of brain metastases and the reduction of survival time in EGFR-mutation lung adenocarcinoma patients with surgery. Lung Cancer, 141, 72-77. doi:10.1016/j.lungcan.2020.01.007
Li, G., Wang, G., Guo, Y., Li, S., Zhang, Y., Li, J., & Peng, B. (2020). Development of a novel prognostic score combining clinicopathologic variables, gene expression, and mutation profiles for lung adenocarcinoma. World J Surg Oncol, 18(1), 249. doi:10.1186/s12957-020-02025-0
Li, H., Dong, S., Zhang, D., Guo, Z., Li, C., Xiang, J., . . . Li, W. (2021). Targeted Sequencing Facilitated Diagnosis of an Uncommon Patient Harboring Both Multiple Primary and Intrapulmonary Metastatic Lung Cancer: A Case Report. Onco Targets Ther, 14, 3455-3459. doi:10.2147/ott.S309155
Li, H., Hardin, H., Zaeem, M., Huang, W., Hu, R., & Lloyd, R. V. (2021). LncRNA expression and SDHB mutations in pheochromocytomas and paragangliomas. Ann Diagn Pathol, 55, 151801. doi:10.1016/j.anndiagpath.2021.151801
Li, H. S., Yang, G. J., & Wang, Y. (2021). Case Report: Dacomitinib May Not Benefit Patients Who Develop Rare Compound Mutations After Later-Line Osimertinib Treatment. Front Oncol, 11, 649843. doi:10.3389/fonc.2021.649843
Li, J., Lin, X., Li, X., Zhang, W., & Sun, D. (2022). Somatic mutations combined with clinical features can predict the postoperative prognosis of stage IIIA lung adenocarcinoma. Ann Transl Med, 10(4), 187. doi:10.21037/atm-22-130
Li, J., You, W., Zheng, D., Yan, B., Ma, X., Pan, Y., . . . Chen, H. (2018). A comprehensive evaluation of clinicopathologic characteristics, molecular features and prognosis in lung adenocarcinoma with solid component. J Cancer Res Clin Oncol, 144(4), 725-734. doi:10.1007/s00432-018-2588-6
Li, L. H., Wu, P., Lee, J. Y., Li, P. R., Hsieh, W. Y., Ho, C. C., . . . Chen, H. W. (2014). Hinokitiol induces DNA damage and autophagy followed by cell cycle arrest and senescence in gefitinib-resistant lung adenocarcinoma cells. PLoS One, 9(8), e104203. doi:10.1371/journal.pone.0104203
Li, N., Zeng, Y., Tai, M., Lin, B., Zhu, D., Luo, Y., . . . Huang, J. (2021). Analysis of the Prognostic Value and Gene Expression Mechanism of SHOX2 in Lung Adenocarcinoma. Front Mol Biosci, 8, 688274. doi:10.3389/fmolb.2021.688274
Li, R., Li, X., Xue, R., Yang, F., Wang, S., Li, Y., . . . Wang, J. (2018). Early metastasis detected in patients with multifocal pulmonary ground-glass opacities (GGOs). Thorax, 73(3), 290-292. doi:10.1136/thoraxjnl-2017-210169
Li, R., Liu, J., Fang, Z., Liang, Z., & Chen, X. (2020). Identification of Mutations Related to Cisplatin-Resistance and Prognosis of Patients With Lung Adenocarcinoma. Front Pharmacol, 11, 572627. doi:10.3389/fphar.2020.572627
Li, S., Zhou, F., Ren, S., & Zhou, C. (2014). Response to pemetrexed rechallenge after acquired resistance of EGFR-TKI in a patient with advanced NSCLC. Lung Cancer, 84(2), 203-205. doi:10.1016/j.lungcan.2014.02.010
Li, S. H., Hsieh, M. H., & Fang, Y. F. (2015). Afatinib in Treatment-Naive Patients With EGFR-Mutated Lung Adenocarcinoma With Brain Metastasis: A Case Series. Medicine (Baltimore), 94(41), e1739. doi:10.1097/md.0000000000001739
Li, T., Piperdi, B., Walsh, W. V., Kim, M., Beckett, L. A., Gucalp, R., . . . Perez-Soler, R. (2017). Randomized Phase 2 Trial of Pharmacodynamic Separation of Pemetrexed and Intercalated Erlotinib Versus Pemetrexed Alone for Advanced Nonsquamous, Non-small-cell Lung Cancer. Clin Lung Cancer, 18(1), 60-67. doi:10.1016/j.cllc.2016.10.003
Li, W., Liu, S., Su, S., Chen, Y., & Sun, G. (2021). Construction and validation of a novel prognostic signature of microRNAs in lung adenocarcinoma. PeerJ, 9, e10470. doi:10.7717/peerj.10470
Li, W., Wu, M., Wang, Q., Xu, K., Lin, F., Wang, Q., & Guo, R. (2020). A comparative genomics analysis of lung adenocarcinoma for Chinese population by using panel of recurrent mutations. J Biomed Res, 35(1), 11-20. doi:10.7555/jbr.34.20200068
Li, X. F., Ren, P., Shen, W. Z., Jin, X., & Zhang, J. (2020). The expression, modulation and use of cancer-testis antigens as potential biomarkers for cancer immunotherapy. Am J Transl Res, 12(11), 7002-7019. 
Li, Y., Zhang, F. S., Guo, L., & Ying, J. M. (2018). [Detection of circulating tumor DNA in epidermal growth factor receptor-TKI relapsed non-small cell lung cancer patients using next-generation sequencing and an analysis of the resistant mechanisms]. Zhonghua Bing Li Xue Za Zhi, 47(12), 904-909. doi:10.3760/cma.j.issn.0529-5807.2018.12.002
Li, Y., Zhang, H. B., Chen, X., Yang, X., Ye, Y., Bekaii-Saab, T., . . . Zhang, Y. (2020). A Rare EGFR-SEPT14 Fusion in a Patient with Colorectal Adenocarcinoma Responding to Erlotinib. Oncologist, 25(3), 203-207. doi:10.1634/theoncologist.2019-0405
Lim, C. A., Banyi, N., Tucker, T., Ionescu, D. N., & Melosky, B. (2022). A Case of ALK-Rearranged Combined Lung Adenocarcinoma and Neuroendocrine Carcinoma with Diffuse Bone Metastasis and Partial Response to Alectinib. Curr Oncol, 29(2), 848-852. doi:10.3390/curroncol29020072
Lim, S. H., Lee, J. Y., Sun, J. M., Ahn, J. S., Park, K., & Ahn, M. J. (2014). Comparison of clinical outcomes following gefitinib and erlotinib treatment in non-small-cell lung cancer patients harboring an epidermal growth factor receptor mutation in either exon 19 or 21. J Thorac Oncol, 9(4), 506-511. doi:10.1097/jto.0000000000000095
Lim, S. M., Choi, J. W., Hong, M. H., Jung, D., Lee, C. Y., Park, S. Y., . . . Kim, H. R. (2019). Indoor radon exposure increases tumor mutation burden in never-smoker patients with lung adenocarcinoma. Lung Cancer, 131, 139-146. doi:10.1016/j.lungcan.2019.04.002
Lin, C. Y., Wu, Y. M., Hsieh, M. H., Wang, C. W., Wu, C. Y., Chen, Y. J., & Fang, Y. F. (2017). Prognostic implication of EGFR gene mutations and histological classification in patients with resected stage I lung adenocarcinoma. PLoS One, 12(10), e0186567. doi:10.1371/journal.pone.0186567
Lin, M. W., Wu, C. T., Kuo, S. W., Chang, Y. L., & Yang, P. C. (2014). Clinicopathology and genetic profile of synchronous multiple small adenocarcinomas: implication for surgical treatment of an uncommon lung malignancy. Ann Surg Oncol, 21(8), 2555-2562. doi:10.1245/s10434-014-3642-5
Lin, M. W., Wu, C. T., Shih, J. Y., Chang, Y. L., & Yang, P. C. (2014). Clinicopathologic characteristics and prognostic significance of EGFR and p53 mutations in surgically resected lung adenocarcinomas ≤2 cm in maximal dimension. J Surg Oncol, 110(2), 99-106. doi:10.1002/jso.23628
Lissa, D., Ishigame, T., Noro, R., Tucker, M. J., Bliskovsky, V., Shema, S., . . . Robles, A. I. (2018). HOXA9 methylation and blood vessel invasion in FFPE tissues for prognostic stratification of stage I lung adenocarcinoma patients. Lung Cancer, 122, 151-159. doi:10.1016/j.lungcan.2018.05.021
Liu, J., Xia, L., Peng, Y., Huang, Y. S., & Yang, Z. Z. (2021). Gastric metastasis and transformation of primary lung adenocarcinoma to small cell cancer after acquired resistance to epidermal growth factor receptor tyrosine kinase inhibitors: A case report. Medicine (Baltimore), 100(39), e27289. doi:10.1097/md.0000000000027289
Liu, L., Huang, J., Wang, K., Li, L., Li, Y., Yuan, J., & Wei, S. (2015). Identification of hallmarks of lung adenocarcinoma prognosis using whole genome sequencing. Oncotarget, 6(35), 38016-38028. doi:10.18632/oncotarget.5697
Liu, M., Zhou, C., & Zheng, J. (2015). Cigarette smoking impairs the response of EGFR-TKIs therapy in lung adenocarcinoma patients by promoting EGFR signaling and epithelial-mesenchymal transition. Am J Transl Res, 7(10), 2026-2035. 
Liu, P., Wang, C., Wu, S., Gao, J., & Zeng, X. (2014). [Clinicopathologic correlation and ALK rearrangement in adenocarcinoma of lung]. Zhonghua Bing Li Xue Za Zhi, 43(4), 241-245. 
Liu, S., Liu, N., Xiao, M., Wang, L., & Wang, E. H. (2020). First case of bronchiolar adenoma lined purely by mucinous luminal cells with molecular analysis: A case report. Medicine (Baltimore), 99(39), e22322. doi:10.1097/md.0000000000022322
Liu, S., Wang, J., Luo, X., Li, X., Miao, Y., Wang, L., . . . Wang, E. H. (2020). Coexistence of Low-Grade Fetal Adenocarcinoma and Adenocarcinoma in situ of the Lung Harboring Different Genetic Mutations: A Case Report and Review of Literature. Onco Targets Ther, 13, 6675-6680. doi:10.2147/ott.S260993
Liu, W., Wei, H., Gao, Z., Chen, G., Liu, Y., Gao, X., . . . Xiao, J. (2018). COL5A1 may contribute the metastasis of lung adenocarcinoma. Gene, 665, 57-66. doi:10.1016/j.gene.2018.04.066
Liu, W. S., Zhao, L. J., Pang, Q. S., Yuan, Z. Y., Li, B., & Wang, P. (2014). Prognostic value of epidermal growth factor receptor mutations in resected lung adenocarcinomas. Med Oncol, 31(1), 771. doi:10.1007/s12032-013-0771-9
Liu, X., Jia, Y., Shi, C., Kong, D., Wu, Y., Zhang, T., . . . Wang, D. (2021). CYP4B1 is a prognostic biomarker and potential therapeutic target in lung adenocarcinoma. PLoS One, 16(2), e0247020. doi:10.1371/journal.pone.0247020
Liu, X., Li, X., Zhang, C., Jin, J., Wang, Z., Xiao, R., . . . Yang, F. (2022). EGFR mutation is not a prognostic factor for CNS metastasis in curatively resected lung adenocarcinoma patients. Lung Cancer, 167, 78-86. doi:10.1016/j.lungcan.2022.03.013
Lococo, F., Gandolfi, G., Rossi, G., Pinto, C., Rapicetta, C., Cavazza, A., . . . Ciarrocchi, A. (2016). Deep Sequencing Analysis Reveals That KRAS Mutation Is a Marker of Poor Prognosis in Patients with Pulmonary Sarcomatoid Carcinoma. J Thorac Oncol, 11(8), 1282-1292. doi:10.1016/j.jtho.2016.04.020
Lorch, G., Sivaprakasam, K., Zismann, V., Perdigones, N., Contente-Cuomo, T., Nazareno, A., . . . Hendricks, W. P. D. (2019). Identification of Recurrent Activating HER2 Mutations in Primary Canine Pulmonary Adenocarcinoma. Clin Cancer Res, 25(19), 5866-5877. doi:10.1158/1078-0432.Ccr-19-1145
Lounglaithong, K., Bychkov, A., & Sampatanukul, P. (2018). Aberrant promoter methylation of the PAQR3 gene is associated with prostate cancer. Pathol Res Pract, 214(1), 126-129. doi:10.1016/j.prp.2017.10.010
Lozano, M. D., Labiano, T., Zudaire, I., Subtil, J. C., Gúrpide, A., Echeveste, J. I., . . . Pérez-Gracia, J. L. (2015). Variations in molecular profile in NSCLC can be analyzed using cytological samples: development of EGFR resistance mutations and coexistence of ALK-EML4 translocation in an EGFR-sensitive patient. Int J Surg Pathol, 23(2), 111-115. doi:10.1177/1066896914539551
Lu, Q., Ma, Y., An, Z., Zhao, T., Xu, Z., & Chen, H. (2018). Epidermal growth factor receptor mutation accelerates radiographic progression in lung adenocarcinoma presented as a solitary ground-glass opacity. J Thorac Dis, 10(11), 6030-6039. doi:10.21037/jtd.2018.10.19
Lu, X., Peled, N., Greer, J., Wu, W., Choi, P., Berger, A. H., . . . Collisson, E. A. (2017). MET Exon 14 Mutation Encodes an Actionable Therapeutic Target in Lung Adenocarcinoma. Cancer Res, 77(16), 4498-4505. doi:10.1158/0008-5472.Can-16-1944
Lui, N. S., Benson, J., He, H., Imielski, B. R., Kunder, C. A., Liou, D. Z., . . . Shrager, J. B. (2020). Sub-solid lung adenocarcinoma in Asian versus Caucasian patients: different biology but similar outcomes. J Thorac Dis, 12(5), 2161-2171. doi:10.21037/jtd.2020.04.37
Luo, S. Y., Sit, K. Y., Sihoe, A. D., Suen, W. S., Au, W. K., Tang, X., . . . Lam, D. C. (2014). Aberrant large tumor suppressor 2 (LATS2) gene expression correlates with EGFR mutation and survival in lung adenocarcinomas. Lung Cancer, 85(2), 282-292. doi:10.1016/j.lungcan.2014.05.025
Lv, C., An, C., Feng, Q., Ma, Y., Li, S., Wang, J., . . . Yang, Y. (2015). A Retrospective Study of Stage I to IIIa Lung Adenocarcinoma After Resection: What Is the Optimal Adjuvant Modality for Patients With an EGFR Mutation? Clin Lung Cancer, 16(6), e173-181. doi:10.1016/j.cllc.2015.04.002
Lv, T., Zou, J., Liu, H., Shen, Q., Lu, Z., Zhou, X., . . . Song, Y. (2017). Detection of oncogenic mutations in resected bronchial margins by next-generation sequencing indicates early relapse in stage IA lung adenocarcinoma patients. Oncotarget, 8(25), 40643-40653. doi:10.18632/oncotarget.16539
Lv, Y. L., Liu, H. B., Yuan, D. M., Zhou, L., Jin, S. X., & Song, Y. (2019). Carcinoembryonic antigen in pleural effusion of patients with lung adenocarcinoma: a predictive marker for EGFR mutation. Transl Cancer Res, 8(4), 1027-1034. doi:10.21037/tcr.2019.06.10
Ma, X., Cheng, J., Zhao, P., Li, L., Tao, K., & Chen, H. (2020). DNA methylation profiling to predict recurrence risk in stage Ι lung adenocarcinoma: Development and validation of a nomogram to clinical management. J Cell Mol Med, 24(13), 7576-7589. doi:10.1111/jcmm.15393
Ma, Y., Zheng, X., Zhao, H., Fang, W., Zhang, Y., Ge, J., . . . Zhang, L. (2018). First-in-Human Phase I Study of AC0010, a Mutant-Selective EGFR Inhibitor in Non-Small Cell Lung Cancer: Safety, Efficacy, and Potential Mechanism of Resistance. J Thorac Oncol, 13(7), 968-977. doi:10.1016/j.jtho.2018.03.025
Ma, Z., Zhang, Y., Deng, C., Fu, F., Deng, L., Li, Y., & Chen, H. (2022). The prognostic value of Kirsten rat sarcoma viral oncogene homolog mutations in resected lung adenocarcinoma differs according to clinical features. J Thorac Cardiovasc Surg, 163(1), e73-e85. doi:10.1016/j.jtcvs.2020.05.097
Mackay, H. L., Moore, D., Hall, C., Birkbak, N. J., Jamal-Hanjani, M., Karim, S. A., . . . Muller, P. A. J. (2018). Genomic instability in mutant p53 cancer cells upon entotic engulfment. Nat Commun, 9(1), 3070. doi:10.1038/s41467-018-05368-1
Maeng, C. H., Lee, H. Y., Kim, Y. W., Choi, M. K., Hong, J. Y., Jung, H. A., . . . Ahn, M. J. (2013). High-throughput molecular genotyping for small biopsy samples in advanced non-small cell lung cancer patients. Anticancer Res, 33(11), 5127-5133. 
Mainetti, C., Guillod, C., & Leoni-Parvex, S. (2016). Successful Treatment of Relapsing Bowen's Disease with Ingenol Mebutate: The Use of Dermoscopy to Monitor the Therapeutic Response. Dermatology, 232 Suppl 1, 9-13. doi:10.1159/000447389
Majd, N. K., Metrus, N. R., Santos-Pinheiro, F., Trevino, C. R., Fuller, G. N., Huse, J. T., . . . Penas-Prado, M. (2019). RBM10 truncation in astroblastoma in a patient with history of mandibular ameloblastoma: A case report. Cancer Genet, 231-232, 41-45. doi:10.1016/j.cancergen.2019.01.001
Majewski, I. J., Mittempergher, L., Davidson, N. M., Bosma, A., Willems, S. M., Horlings, H. M., . . . Bernards, R. (2013). Identification of recurrent FGFR3 fusion genes in lung cancer through kinome-centred RNA sequencing. J Pathol, 230(3), 270-276. doi:10.1002/path.4209
Mak, R. H., Hermann, G., Lewis, J. H., Aerts, H. J., Baldini, E. H., Chen, A. B., . . . Sher, D. J. (2015). Outcomes by tumor histology and KRAS mutation status after lung stereotactic body radiation therapy for early-stage non-small-cell lung cancer. Clin Lung Cancer, 16(1), 24-32. doi:10.1016/j.cllc.2014.09.005
Maki, S., Igawa, S., Otani, S., Nagashima, Y., Kimura, M., Hiyoshi, Y., . . . Masuda, N. (2012). [Efficacy of chemotherapy with carboplatin-paclitaxel plus bevacizumab for previously treated patients with advanced non-small cell carcinoma]. Gan To Kagaku Ryoho, 39(13), 2509-2512. 
Makimoto, G., Ninomiya, K., Kubo, T., Sunami, R., Kato, Y., Ichihara, E., . . . Kiura, K. (2021). A novel osimertinib-resistant human lung adenocarcinoma cell line harbouring mutant EGFR and activated IGF1R. Jpn J Clin Oncol, 51(6), 956-965. doi:10.1093/jjco/hyab048
Mäki-Nevala, S., Sarhadi, V. K., Rönty, M., Kettunen, E., Husgafvel-Pursiainen, K., Wolff, H., . . . Knuutila, S. (2016). Hot spot mutations in Finnish non-small cell lung cancers. Lung Cancer, 99, 102-110. doi:10.1016/j.lungcan.2016.06.024
Manceau, G., Letouzé, E., Guichard, C., Didelot, A., Cazes, A., Corté, H., . . . Blons, H. (2013). Recurrent inactivating mutations of ARID2 in non-small cell lung carcinoma. Int J Cancer, 132(9), 2217-2221. doi:10.1002/ijc.27900
Marcoux, N., Gettinger, S. N., O'Kane, G., Arbour, K. C., Neal, J. W., Husain, H., . . . Sequist, L. V. (2019). EGFR-Mutant Adenocarcinomas That Transform to Small-Cell Lung Cancer and Other Neuroendocrine Carcinomas: Clinical Outcomes. J Clin Oncol, 37(4), 278-285. doi:10.1200/jco.18.01585
Mas, C., Boda, B., CaulFuty, M., Huang, S., Wiszniewski, L., & Constant, S. (2015). Antitumour efficacy of the selumetinib and trametinib MEK inhibitors in a combined human airway-tumour-stroma lung cancer model. J Biotechnol, 205, 111-119. doi:10.1016/j.jbiotec.2015.01.012
Masubuchi, H., Maeno, T., Uchida, M., Kono, S., Suzuki, M., Takemura, M., . . . Kurabayashi, M. (2015). A case of Trousseau syndrome caused by pulmonary adenocarcinoma that was controlled for one year and 10 months with thrombosis treatment using an EGFR tyrosine kinase inhibitor and chemotherapy. Respir Med Case Rep, 15, 101-105. doi:10.1016/j.rmcr.2015.05.001
Matsuda, A., Fuchimoto, Y., Wada, S., Tanaka, T., Takeguchi, T., Mitsumune, S., . . . Fujimoto, N. (2019). Rebiopsy with Thoracoscopy under Local Anesthesia for the Detection of EGFR T790M Mutation. Case Rep Oncol, 12(3), 918-921. doi:10.1159/000504932
Matsui, T., Sakakura, N., Koyama, S., Nakanishi, K., Sasaki, E., Kato, S., . . . Yatabe, Y. (2021). Comparison of Surgical Outcomes Between Invasive Mucinous and Non-Mucinous Lung Adenocarcinoma. Ann Thorac Surg, 112(4), 1118-1126. doi:10.1016/j.athoracsur.2020.09.042
Matsumoto, Y., Kawaguchi, T., Yamamoto, N., Sawa, K., Yoshimoto, N., Suzumura, T., . . . Hirata, K. (2017). Interstitial Lung Disease Induced by Osimertinib for Epidermal Growth Factor Receptor (EGFR) T790M-positive Non-small Cell Lung Cancer. Intern Med, 56(17), 2325-2328. doi:10.2169/internalmedicine.8467-16
Matsumura, M., Okudela, K., Kojima, Y., Umeda, S., Tateishi, Y., Sekine, A., . . . Ohashi, K. (2016). A Histopathological Feature of EGFR-Mutated Lung Adenocarcinomas with Highly Malignant Potential - An Implication of Micropapillary Element. PLoS One, 11(11), e0166795. doi:10.1371/journal.pone.0166795
Matsumura, M., Okudela, K., Nakashima, Y., Mitsui, H., Denda-Nagai, K., Suzuki, T., . . . Ohashi, K. (2019). Specific expression of MUC21 in micropapillary elements of lung adenocarcinomas - Implications for the progression of EGFR-mutated lung adenocarcinomas. PLoS One, 14(4), e0215237. doi:10.1371/journal.pone.0215237
Matsumura, Y., Owada, Y., Yamaura, T., Muto, S., Osugi, J., Hoshino, M., . . . Gotoh, M. (2016). Epidermal growth factor receptor gene mutation as risk factor for recurrence in patients with surgically resected lung adenocarcinoma: a matched-pair analysis. Interact Cardiovasc Thorac Surg, 23(2), 216-222. doi:10.1093/icvts/ivw116
Matsumura, Y., Suzuki, H., Ohira, T., Shiono, S., Abe, J., Sagawa, M., . . . Okada, Y. (2017). Matched-pair analysis of a multi-institutional cohort reveals that epidermal growth factor receptor mutation is not a risk factor for postoperative recurrence of lung adenocarcinoma. Lung Cancer, 114, 23-30. doi:10.1016/j.lungcan.2017.09.003
Matsushima, J., Yazawa, T., Suzuki, M., Takahashi, Y., Ota, S., Nakajima, T., . . . Nakatani, Y. (2017). Clinicopathological, immunohistochemical, and mutational analyses of pulmonary enteric adenocarcinoma: usefulness of SATB2 and β-catenin immunostaining for differentiation from metastatic colorectal carcinoma. Hum Pathol, 64, 179-185. doi:10.1016/j.humpath.2017.04.006
Matsuura, Y., Ninomiya, H., Ichinose, J., Nakao, M., Okumura, S., Nishio, M., & Mun, M. (2022). Prognostic impact and distinctive characteristics of surgically resected anaplastic lymphoma kinase-rearranged lung adenocarcinoma. J Thorac Cardiovasc Surg, 163(2), 441-451.e441. doi:10.1016/j.jtcvs.2020.09.120
McFadden, D. G., Politi, K., Bhutkar, A., Chen, F. K., Song, X., Pirun, M., . . . Varmus, H. (2016). Mutational landscape of EGFR-, MYC-, and Kras-driven genetically engineered mouse models of lung adenocarcinoma. Proc Natl Acad Sci U S A, 113(42), E6409-e6417. doi:10.1073/pnas.1613601113
Mehta, M. P., & Kotecha, R. (2018). Postoperative Management of Resected Brain Metastases: When Can Radiotherapy Be Deferred? J Clin Oncol, Jco2018793232. doi:10.1200/jco.2018.79.3232
Miao, Q., Zhang, L., Zheng, X., Jiang, K., Wu, B., & Lin, G. (2021). Transformation of a cold to hot tumor and a durable response to immunotherapy in a patient with non-small cell lung cancer after chemoradiotherapy: a case report. Ann Palliat Med, 10(4), 4982-4986. doi:10.21037/apm-21-761
Minemura, H., Yokouchi, H., Azuma, K., Hirai, K., Sekine, S., Oshima, K., . . . Munakata, M. (2015). A phase II trial of erlotinib monotherapy for pretreated elderly patients with advanced EGFR wild-type non-small cell lung cancer. BMC Res Notes, 8, 220. doi:10.1186/s13104-015-1214-9
Mitchell, K. A., Nichols, N., Tang, W., Walling, J., Stevenson, H., Pineda, M., . . . Ryan, B. M. (2019). Recurrent PTPRT/JAK2 mutations in lung adenocarcinoma among African Americans. Nat Commun, 10(1), 5735. doi:10.1038/s41467-019-13732-y
Mitchell, K. A., Nichols, N., Tang, W., Walling, J., Stevenson, H., Pineda, M., . . . Ryan, B. M. (2020). Author Correction: Recurrent PTPRT/JAK2 mutations in lung adenocarcinoma among African Americans. Nat Commun, 11(1), 700. doi:10.1038/s41467-020-14448-0
Mitchell, K. G., Parra, E. R., Zhang, J., Nelson, D. B., Corsini, E. M., Villalobos, P., . . . Antonoff, M. B. (2020). LKB1/STK11 Expression in Lung Adenocarcinoma and Associations With Patterns of Recurrence. Ann Thorac Surg, 110(4), 1131-1138. doi:10.1016/j.athoracsur.2020.03.114
Miyagaki, H., Yamasaki, M., Takahashi, T., Kurokawa, Y., Miyata, H., Nakajima, K., . . . Doki, Y. (2012). DOK2 as a marker of poor prognosis of patients with gastric adenocarcinoma after curative resection. Ann Surg Oncol, 19(5), 1560-1567. doi:10.1245/s10434-011-2157-6
Miyawaki, M., Naoki, K., Yoda, S., Nakayama, S., Satomi, R., Sato, T., . . . Soejima, K. (2017). Erlotinib as second- or third-line treatment in elderly patients with advanced non-small cell lung cancer: Keio Lung Oncology Group Study 001 (KLOG001). Mol Clin Oncol, 6(3), 409-414. doi:10.3892/mco.2017.1154
Morales-Oyarvide, V., & Mino-Kenudson, M. (2014). High-grade lung adenocarcinomas with micropapillary and/or solid patterns: a review. Curr Opin Pulm Med, 20(4), 317-323. doi:10.1097/mcp.0000000000000070
Morio, A., Nakahara, K., Futagawa, T., & Suzuki, K. (2016). [Epidermal Growth Factor Receptor-mutated Advanced Lung Adenocarcinoma with Long-term Tumor-free Survival by Chemotherapy Followed by Tyrosine Kinase Inhibitor Treatment after Reduction Surgery]. Kyobu Geka, 69(13), 1051-1054. 
Morishita, M., Suzuki, M., Watanabe, H., Morita, C., Ishida, A., Hashimoto, M., . . . Sugiyama, H. (2021). Diagnosis of miliary nodules as lung adenocarcinoma by cryobiopsy: A case report. Thorac Cancer, 12(10), 1613-1616. doi:10.1111/1759-7714.13946
Moriya, R., Hokari, S., Shibata, S., Koizumi, T., Tetsuka, T., Ito, K., . . . Tsukada, H. (2017). Histological Transformation to Large Cell Neuroendocrine Carcinoma from Lung Adenocarcinoma Harboring an EGFR Mutation: An Autopsy Case Report. Intern Med, 56(15), 2013-2017. doi:10.2169/internalmedicine.56.7452
Morris, P. G., Reiner, A. S., Szenberg, O. R., Clarke, J. L., Panageas, K. S., Perez, H. R., . . . Omuro, A. M. (2012). Leptomeningeal metastasis from non-small cell lung cancer: survival and the impact of whole brain radiotherapy. J Thorac Oncol, 7(2), 382-385. doi:10.1097/JTO.0b013e3182398e4f
Motono, N., Funasaki, A., Sekimura, A., Usuda, K., & Uramoto, H. (2018). Prognostic value of epidermal growth factor receptor mutations and histologic subtypes with lung adenocarcinoma. Med Oncol, 35(3), 22. doi:10.1007/s12032-018-1082-y
Murayama, T., Nakaoku, T., Enari, M., Nishimura, T., Tominaga, K., Nakata, A., . . . Gotoh, N. (2016). Oncogenic Fusion Gene CD74-NRG1 Confers Cancer Stem Cell-like Properties in Lung Cancer through a IGF2 Autocrine/Paracrine Circuit. Cancer Res, 76(4), 974-983. doi:10.1158/0008-5472.Can-15-2135
Muto, S., Ozaki, Y., Okabe, N., Matsumura, Y., Hasegawa, T., Shio, Y., . . . Suzuki, H. (2020). Successful Treatment of Combined Large Cell Neuroendocrine Carcinoma Harboring an EGFR Mutation with EGFR-TKIs plus Bevacizumab: A Case Report. Case Rep Oncol, 13(3), 1387-1392. doi:10.1159/000511112
Na, II, Kim, H. R., Lee, J. K., Park, S. H., Kim, C. H., Koh, J. S., . . . Choe du, H. (2012). Epidermal growth factor receptor mutations in female patients with postoperative recurrent non-small-cell lung cancer. J Cancer Res Ther, 8(3), 373-378. doi:10.4103/0973-1482.103515
Nakajima, N., Yoshizawa, A., Rokutan-Kurata, M., Noguchi, M., Teramoto, Y., Sumiyoshi, S., . . . Haga, H. (2021). Prognostic significance of cribriform adenocarcinoma of the lung: validation analysis of 1,057 Japanese patients with resected lung adenocarcinoma and a review of the literature. Transl Lung Cancer Res, 10(1), 117-127. doi:10.21037/tlcr-20-612
Nakamichi, S., Kubota, K., Horinouchi, H., Kanda, S., Fujiwara, Y., Nokihara, H., . . . Tamura, T. (2013). Successful EGFR-TKI rechallenge of leptomeningeal carcinomatosis after gefitinib-induced interstitial lung disease. Jpn J Clin Oncol, 43(4), 422-425. doi:10.1093/jjco/hyt012
Nakano, Y., Isobe, K., Kobayashi, H., Kaburaki, K., Isshiki, T., Sakamoto, S., . . . Kishi, K. (2020). Clinical importance of long non‑coding RNA LINC00460 expression in EGFR‑mutant lung adenocarcinoma. Int J Oncol, 56(1), 243-257. doi:10.3892/ijo.2019.4919
Nakano, Y., Koide, N., Koyama, Y., Nitta, K., & Koizumi, T. (2021). Lung adenocarcinoma initially presenting as Trousseau's syndrome treated successfully with pembrolizumab: A case report. Thorac Cancer, 12(4), 557-559. doi:10.1111/1759-7714.13794
Neri, S., Menju, T., Sowa, T., Yutaka, Y., Nakajima, D., Hamaji, M., . . . Date, H. (2019). Prognostic impact of microscopic vessel invasion and visceral pleural invasion and their correlations with epithelial-mesenchymal transition, cancer stemness, and treatment failure in lung adenocarcinoma. Lung Cancer, 128, 13-19. doi:10.1016/j.lungcan.2018.12.001
Nguyen, N. C., & Osman, M. M. (2018). Normalized Subtraction of Serial Brain Magnetic Resonance Images and Fludeoxyglucose-Positron Emission Tomography Images for Tumor Treatment Monitoring: Case Report and Method Description. J Clin Imaging Sci, 8, 25. doi:10.4103/jcis.JCIS_14_18
Nie, Y., Gao, W., Li, N., Chen, W., Wang, H., Li, C., . . . Liu, H. (2017). Relationship between EGFR gene mutation and local metastasis of resectable lung adenocarcinoma. World J Surg Oncol, 15(1), 55. doi:10.1186/s12957-017-1103-x
Niederst, M. J., Sequist, L. V., Poirier, J. T., Mermel, C. H., Lockerman, E. L., Garcia, A. R., . . . Engelman, J. A. (2015). RB loss in resistant EGFR mutant lung adenocarcinomas that transform to small-cell lung cancer. Nat Commun, 6, 6377. doi:10.1038/ncomms7377
Ning, Y., Liu, W., Guan, X., Xie, X., & Zhang, Y. (2019). CPSF3 is a promising prognostic biomarker and predicts recurrence of non-small cell lung cancer. Oncol Lett, 18(3), 2835-2844. doi:10.3892/ol.2019.10659
Nishii, K., Ohashi, K., Tamura, T., Ninomiya, K., Matsubara, T., Senoo, S., . . . Kiura, K. (2020). Detection of epidermal growth factor receptor mutations in exhaled breath condensate using droplet digital polymerase chain reaction. Oncol Lett, 20(6), 393. doi:10.3892/ol.2020.12256
Nishii, T., Yokose, T., Miyagi, Y., Daigo, Y., Isaka, T., Furumoto, H., . . . Masuda, M. (2017). Prognostic value of EGFR mutations in surgically resected pathological stage I lung adenocarcinoma. Asia Pac J Clin Oncol, 13(5), e204-e211. doi:10.1111/ajco.12512
Nishikawa, S., Menju, T., Takahashi, K., Miyata, R., Chen-Yoshikawa, T. F., Sonobe, M., . . . Date, H. (2019). Statins may have double-edged effects in patients with lung adenocarcinoma after lung resection. Cancer Manag Res, 11, 3419-3432. doi:10.2147/cmar.S200819
Nomura, K., Aokage, K., Nakai, T., Sakashita, S., Miyoshi, T., Tane, K., . . . Ishii, G. (2021). Prognostic impact of extranodal extension in patients with pN1-N2 lung adenocarcinoma. J Cancer Res Clin Oncol, 147(12), 3699-3707. doi:10.1007/s00432-021-03608-4
Noonan, S. A., Patil, T., Gao, D., King, G. G., Thibault, J. R., Lu, X., . . . Camidge, D. R. (2018). Baseline and On-Treatment Characteristics of Serum Tumor Markers in Stage IV Oncogene-Addicted Adenocarcinoma of the Lung. J Thorac Oncol, 13(1), 134-138. doi:10.1016/j.jtho.2017.08.005
Noro, R., Honda, K., Nagashima, K., Motoi, N., Kunugi, S., Matsubayashi, J., . . . Kubota, K. (2022). Alpha-actinin-4 (ACTN4) gene amplification is a predictive biomarker for adjuvant chemotherapy with tegafur/uracil in stage I lung adenocarcinomas. Cancer Sci, 113(3), 1002-1009. doi:10.1111/cas.15228
Noro, R., Honda, K., Tsuta, K., Ishii, G., Maeshima, A. M., Miura, N., . . . Yamada, T. (2013). Distinct outcome of stage I lung adenocarcinoma with ACTN4 cell motility gene amplification. Ann Oncol, 24(10), 2594-2600. doi:10.1093/annonc/mdt293
Noro, R., Kobayashi, K., Usuki, J., Yomota, M., Nishitsuji, M., Shimokawa, T., . . . Gemma, A. (2020). Bevacizumab plus chemotherapy in nonsquamous non-small cell lung cancer patients with malignant pleural effusion uncontrolled by tube drainage or pleurodesis: A phase II study North East Japan Study group trial NEJ013B. Thorac Cancer, 11(7), 1876-1884. doi:10.1111/1759-7714.13472
Notsuda, H., Sakurada, A., Endo, C., Okada, Y., Horii, A., Shima, H., & Kondo, T. (2013). p190A RhoGAP is involved in EGFR pathways and promotes proliferation, invasion and migration in lung adenocarcinoma cells. Int J Oncol, 43(5), 1569-1577. doi:10.3892/ijo.2013.2096
Nukii, Y., Miyamoto, A., Mochizuki, S., Moriguchi, S., Takahashi, Y., Ogawa, K., . . . Kishi, K. (2019). Pneumatosis intestinalis induced by osimertinib in a patient with lung adenocarcinoma harbouring epidermal growth factor receptor gene mutation with simultaneously detected exon 19 deletion and T790 M point mutation: a case report. BMC Cancer, 19(1), 186. doi:10.1186/s12885-019-5399-5
Ohashi, R., Umezu, H., Sato, A., Abé, T., Kondo, S., Daigo, K., . . . Ajioka, Y. (2020). Frequent Germline and Somatic Single Nucleotide Variants in the Promoter Region of the Ribosomal RNA Gene in Japanese Lung Adenocarcinoma Patients. Cells, 9(11). doi:10.3390/cells9112409
O'Hayer, K., Farber, J., Yeo, C. J., & Sama, A. R. (2015). HER-2-Positive Ampullary Adenocarcinoma: A Case Report. Case Rep Pancreat Cancer, 1(1), 7-10. doi:10.1089/crpc.2015.29004.koh
Ohba, T., Toyokawa, G., Kometani, T., Nosaki, K., Hirai, F., Yamaguchi, M., . . . Sugio, K. (2014). Mutations of the EGFR and K-ras genes in resected stage I lung adenocarcinoma and their clinical significance. Surg Today, 44(3), 478-486. doi:10.1007/s00595-013-0589-2
Ohba, T., Toyokawa, G., Osoegawa, A., Hirai, F., Yamaguchi, M., Taguchi, K., . . . Sugio, K. (2016). Mutations of the EGFR, K-ras, EML4-ALK, and BRAF genes in resected pathological stage I lung adenocarcinoma. Surg Today, 46(9), 1091-1098. doi:10.1007/s00595-015-1295-z
Ohtaki, Y., Shimizu, K., Kakegawa, S., Nagashima, T., Nakano, T., Atsumi, J., . . . Takeyoshi, I. (2014). Postrecurrence survival of surgically resected pulmonary adenocarcinoma patients according to EGFR and KRAS mutation status. Mol Clin Oncol, 2(2), 187-196. doi:10.3892/mco.2013.237
Oiwa, H., Aokage, K., Suzuki, A., Sato, K., Kuroe, T., Mimaki, S., . . . Ishii, G. (2021). Clinicopathological, gene expression and genetic features of stage I lung adenocarcinoma with necrosis. Lung Cancer, 159, 74-83. doi:10.1016/j.lungcan.2021.07.001
Okamoto, T., Kitahara, H., Shimamatsu, S., Katsura, M., Takada, K., Fujishita, T., . . . Maehara, Y. (2016). Prognostic Impact of EGFR Driver Mutations on Postoperative Disease Recurrence in Lung Adenocarcinoma. Anticancer Res, 36(6), 3057-3063. 
Okamoto, T., Kohno, M., Ito, K., Takada, K., Katsura, M., Morodomi, Y., . . . Maehara, Y. (2017). Clinical Significance of DNA Damage Response Factors and Chromosomal Instability in Primary Lung Adenocarcinoma. Anticancer Res, 37(4), 1729-1735. doi:10.21873/anticanres.11505
Okita, R., Yukawa, T., Nojima, Y., Maeda, A., Saisho, S., Shimizu, K., & Nakata, M. (2016). MHC class I chain-related molecule A and B expression is upregulated by cisplatin and associated with good prognosis in patients with non-small cell lung cancer. Cancer Immunol Immunother, 65(5), 499-509. doi:10.1007/s00262-016-1814-9
Okuma, Y., Hosomi, Y., & Imamura, A. (2015). Lung cancer patients harboring epidermal growth factor receptor mutation among those infected by human immunodeficiency virus. Onco Targets Ther, 8, 111-115. doi:10.2147/ott.S76712
Okuma, Y., Hosomi, Y., Nagamata, M., Yamada, Y., Sekihara, K., Kato, K., . . . Okamura, T. (2013). Clinical outcomes after first-line EGFR inhibitor treatment for patients with NSCLC, EGFR mutation, and poor performance status. Anticancer Res, 33(11), 5057-5064. 
Ono, A., Isaka, M., Serizawa, M., Omae, K., Kojima, H., Nakashima, K., . . . Ohde, Y. (2019). Genetic alterations of driver genes as independent prognostic factors for disease-free survival in patients with resected non-small cell lung cancer. Lung Cancer, 128, 152-157. doi:10.1016/j.lungcan.2018.12.005
Ono, A., Kenmotsu, H., Watanabe, M., Serizawa, M., Mori, K., Imai, H., . . . Takahashi, T. (2014). Mutant allele frequency predicts the efficacy of EGFR-TKIs in lung adenocarcinoma harboring the L858R mutation. Ann Oncol, 25(10), 1948-1953. doi:10.1093/annonc/mdu251
Ono, Y., Takada, K., Osoegawa, A., Kinoshita, F., Oba, T., Tsukamoto, S., . . . Mori, M. (2021). First-line osimertinib for leptomeningeal metastasis from lung adenocarcinoma with EGFR mutation as the initial and solitary site of postoperative recurrence. Int Cancer Conf J, 10(1), 78-82. doi:10.1007/s13691-020-00453-z
Onozato, M. L., Kovach, A. E., Yeap, B. Y., Morales-Oyarvide, V., Klepeis, V. E., Tammireddy, S., . . . Mino-Kenudson, M. (2013). Tumor islands in resected early-stage lung adenocarcinomas are associated with unique clinicopathologic and molecular characteristics and worse prognosis. Am J Surg Pathol, 37(2), 287-294. doi:10.1097/PAS.0b013e31826885fb
Ortiz-Cuaran, S., Scheffler, M., Plenker, D., Dahmen, L., Scheel, A. H., Fernandez-Cuesta, L., . . . Sos, M. L. (2016). Heterogeneous Mechanisms of Primary and Acquired Resistance to Third-Generation EGFR Inhibitors. Clin Cancer Res, 22(19), 4837-4847. doi:10.1158/1078-0432.Ccr-15-1915
Osawa, J., Shimada, Y., Maehara, S., Hagiwara, M., Kakihana, M., Kajiwara, N., . . . Ikeda, N. (2021). Clinical usefulness of the 3-tier classification according to the proportion of morphological patterns for patients with pathological stage I invasive lung adenocarcinoma. Gen Thorac Cardiovasc Surg, 69(6), 943-949. doi:10.1007/s11748-020-01559-0
Ota, T., Hasegawa, Y., Okimura, A., Sakashita, K., Sunami, T., Yukimoto, K., . . . Fukuoka, M. (2018). Breast metastasis from EGFR-mutated lung adenocarcinoma: A case report and review of the literature. Clin Case Rep, 6(8), 1510-1516. doi:10.1002/ccr3.1636
Otsuka, T., Tanaka, A., Azukizawa, H., Sasaki, S., Ishijima, M., Matsuki, T., . . . Kijima, T. (2017). Successful treatment with gefitinib after Stevens-Johnson syndrome associated with afatinib therapy in a patient with adenocarcinoma of the lung. Int Cancer Conf J, 6(1), 38-41. doi:10.1007/s13691-016-0269-3
Ouyang, W. W., Li, Q. Y., Yang, W. G., Su, S. F., Wu, L. J., Yang, Y., & Lu, B. (2021). Genetic characteristics of a patient with multiple primary cancers: A case report. World J Clin Cases, 9(28), 8563-8570. doi:10.12998/wjcc.v9.i28.8563
Paik, J. H., Choi, C. M., Kim, H., Jang, S. J., Choe, G., Kim, D. K., . . . Chung, J. H. (2012). Clinicopathologic implication of ALK rearrangement in surgically resected lung cancer: a proposal of diagnostic algorithm for ALK-rearranged adenocarcinoma. Lung Cancer, 76(3), 403-409. doi:10.1016/j.lungcan.2011.11.008
Park, C. K., Oh, I. J., Choi, Y. D., Jang, T. W., Lee, J. E., Ryu, J. S., . . . Kim, Y. C. (2018). A Prospective Observational Study Evaluating the Correlation of c-MET Expression and EGFR Gene Mutation with Response to Erlotinib as Second-Line Treatment for Patients with Advanced/Metastatic Non-Small-Cell Lung Cancer. Oncology, 94(6), 373-382. doi:10.1159/000486896
Park, I. K., Hyun, K., Kim, E. R., Park, S., Kang, C. H., & Kim, Y. T. (2018). The prognostic effect of the epidermal growth factor receptor gene mutation on recurrence dynamics of lung adenocarcinoma. Eur J Cardiothorac Surg, 54(6), 1022-1027. doi:10.1093/ejcts/ezy220
Park, J. H., You, G. L., Ahn, M. J., Kim, S. W., Hong, M. H., Han, J. Y., . . . Kang, J. H. (2021). Real-world outcomes of anti-PD1 antibodies in platinum-refractory, PD-L1-positive recurrent and/or metastatic non-small cell lung cancer, and its potential practical predictors: first report from Korean Cancer Study Group LU19-05. J Cancer Res Clin Oncol, 147(8), 2459-2469. doi:10.1007/s00432-021-03527-4
Park, S., Ahn, B. C., Lim, S. W., Sun, J. M., Kim, H. R., Hong, M. H., . . . Ahn, M. J. (2018). Characteristics and Outcome of ROS1-Positive Non-Small Cell Lung Cancer Patients in Routine Clinical Practice. J Thorac Oncol, 13(9), 1373-1382. doi:10.1016/j.jtho.2018.05.026
Pazarentzos, E., Giannikopoulos, P., Hrustanovic, G., St John, J., Olivas, V. R., Gubens, M. A., . . . Bivona, T. G. (2016). Oncogenic activation of the PI3-kinase p110β isoform via the tumor-derived PIK3Cβ(D1067V) kinase domain mutation. Oncogene, 35(9), 1198-1205. doi:10.1038/onc.2015.173
Peinado, P., Andrades, A., Cuadros, M., Rodriguez, M. I., Coira, I. F., Garcia, D. J., . . . Medina, P. P. (2022). Multi-omic alterations of the SWI/SNF complex define a clinical subgroup in lung adenocarcinoma. Clin Epigenetics, 14(1), 42. doi:10.1186/s13148-022-01261-3
Peinado, P., Andrades, A., Martorell-Marugán, J., Haswell, J. R., Slack, F. J., Carmona-Sáez, P., & Medina, P. P. (2021). The SWI/SNF complex regulates the expression of miR-222, a tumor suppressor microRNA in lung adenocarcinoma. Hum Mol Genet, 30(23), 2263-2271. doi:10.1093/hmg/ddab187
Pelosi, G., Gasparini, P., Conte, D., Fabbri, A., Perrone, F., Tamborini, E., . . . Sozzi, G. (2016). Synergistic Activation upon MET and ALK Coamplification Sustains Targeted Therapy in Sarcomatoid Carcinoma, a Deadly Subtype of Lung Cancer. J Thorac Oncol, 11(5), 718-728. doi:10.1016/j.jtho.2016.01.009
Pezzuto, A., Terzo, F., Graziani, M. L., Ricci, A., Bruno, P., & Mariotta, S. (2017). Lung cancer requires multidisciplinary treatment to improve patient survival: A case report. Oncol Lett, 14(3), 3035-3038. doi:10.3892/ol.2017.6511
Pietrasz, D., Pécuchet, N., Fabre, E., Blons, H., Chevalier, L., Taly, V., . . . Bachet, J. B. (2016). [What future for circulating tumor DNA? Current data and prospects in colorectal, non-small cell lung and pancreatic cancers]. Bull Cancer, 103(1), 55-65. doi:10.1016/j.bulcan.2015.10.017
Pikor, L. A., Ramnarine, V. R., Lam, S., & Lam, W. L. (2013). Genetic alterations defining NSCLC subtypes and their therapeutic implications. Lung Cancer, 82(2), 179-189. doi:10.1016/j.lungcan.2013.07.025
Pirazzoli, V., Ayeni, D., Meador, C. B., Sanganahalli, B. G., Hyder, F., de Stanchina, E., . . . Politi, K. (2016). Afatinib plus Cetuximab Delays Resistance Compared to Single-Agent Erlotinib or Afatinib in Mouse Models of TKI-Naïve EGFR L858R-Induced Lung Adenocarcinoma. Clin Cancer Res, 22(2), 426-435. doi:10.1158/1078-0432.Ccr-15-0620
Planchard, D., Besse, B., Groen, H. J. M., Souquet, P. J., Quoix, E., Baik, C. S., . . . Johnson, B. E. (2016). Dabrafenib plus trametinib in patients with previously treated BRAF(V600E)-mutant metastatic non-small cell lung cancer: an open-label, multicentre phase 2 trial. Lancet Oncol, 17(7), 984-993. doi:10.1016/s1470-2045(16)30146-2
Planck, M., Edlund, K., Botling, J., Micke, P., Isaksson, S., & Staaf, J. (2013). Genomic and transcriptional alterations in lung adenocarcinoma in relation to EGFR and KRAS mutation status. PLoS One, 8(10), e78614. doi:10.1371/journal.pone.0078614
Plenker, D., Riedel, M., Brägelmann, J., Dammert, M. A., Chauhan, R., Knowles, P. P., . . . Sos, M. L. (2017). Drugging the catalytically inactive state of RET kinase in RET-rearranged tumors. Sci Transl Med, 9(394). doi:10.1126/scitranslmed.aah6144
Plodkowski, A. J., Drilon, A., Halpenny, D. F., O'Driscoll, D., Blair, D., Litvak, A. M., . . . Ginsberg, M. S. (2015). From genotype to phenotype: Are there imaging characteristics associated with lung adenocarcinomas harboring RET and ROS1 rearrangements? Lung Cancer, 90(2), 321-325. doi:10.1016/j.lungcan.2015.09.018
Polonio-Alcalá, E., Rabionet, M., Ruiz-Martínez, S., Palomeras, S., Porta, R., Vásquez-Dongo, C., . . . Ciurana, J. (2021). Polycaprolactone Electrospun Scaffolds Produce an Enrichment of Lung Cancer Stem Cells in Sensitive and Resistant EGFRm Lung Adenocarcinoma. Cancers (Basel), 13(21). doi:10.3390/cancers13215320
Press, R. H., Zhang, C., Cassidy, R. J., Ferris, M. J., Zhong, J., Steuer, C. E., . . . Higgins, K. A. (2018). Targeted sequencing and intracranial outcomes of patients with lung adenocarcinoma brain metastases treated with radiotherapy. Cancer, 124(17), 3586-3595. doi:10.1002/cncr.31589
Pros, E., Saigi, M., Alameda, D., Gomez-Mariano, G., Martinez-Delgado, B., Alburquerque-Bejar, J. J., . . . Sanchez-Cespedes, M. (2020). Genome-wide profiling of non-smoking-related lung cancer cells reveals common RB1 rearrangements associated with histopathologic transformation in EGFR-mutant tumors. Ann Oncol, 31(2), 274-282. doi:10.1016/j.annonc.2019.09.001
Pu, X., Xu, T., Ge, C., He, Y., Yang, X., & Chang, P. (2020). A case of durvalumab-treated double primary cancers of the colon and lung. Ann Palliat Med, 9(5), 3614-3622. doi:10.21037/apm-20-1086
Qi, L., Li, X., He, L., Cheng, G., Cai, Y., Xue, K., & Li, M. (2021). Comparison of Diagnostic Performance of Spread Through Airspaces of Lung Adenocarcinoma Based on Morphological Analysis and Perinodular and Intranodular Radiomic Features on Chest CT Images. Front Oncol, 11, 654413. doi:10.3389/fonc.2021.654413
Qiu, T., Li, W., Zhang, F., Wang, B., & Ying, J. (2020). Major challenges in accurate mutation detection of multifocal lung adenocarcinoma by next-generation sequencing. Cancer Biol Ther, 21(2), 170-177. doi:10.1080/15384047.2019.1674070
Qu, R., Ye, F., Tu, D., Cai, Y., & Fu, X. (2021). Clinical Features and Surgical Treatment of Synchronous Multiple Primary Lung Adenocarcinomas With Different EGFR Mutations. Front Oncol, 11, 785777. doi:10.3389/fonc.2021.785777
Quek, K., Li, J., Estecio, M., Zhang, J., Fujimoto, J., Roarty, E., . . . Zhang, J. (2017). DNA methylation intratumor heterogeneity in localized lung adenocarcinomas. Oncotarget, 8(13), 21994-22002. doi:10.18632/oncotarget.15777
Rabadán, R., Mohamedi, Y., Rubin, U., Chu, T., Alghalith, A. N., Elliott, O., . . . Cámara, P. G. (2020). Identification of relevant genetic alterations in cancer using topological data analysis. Nat Commun, 11(1), 3808. doi:10.1038/s41467-020-17659-7
Rau, K. M., Chen, H. K., Shiu, L. Y., Chao, T. L., Lo, Y. P., Wang, C. C., . . . Huang, C. C. (2016). Discordance of Mutation Statuses of Epidermal Growth Factor Receptor and K-ras between Primary Adenocarcinoma of Lung and Brain Metastasis. Int J Mol Sci, 17(4), 524. doi:10.3390/ijms17040524
Ren, X., Cai, X., Li, J., Zhang, X., Yu, J., Song, X., . . . Song, X. (2020). Histological transformation of lung adenocarcinoma to small cell lung cancer with mutant C797S conferring acquired resistance to osimertinib. J Int Med Res, 48(6), 300060520927918. doi:10.1177/0300060520927918
Renaud, S., Falcoz, P. E., Schaëffer, M., Guenot, D., Romain, B., Olland, A., . . . Massard, G. (2015). Prognostic value of the KRAS G12V mutation in 841 surgically resected Caucasian lung adenocarcinoma cases. Br J Cancer, 113(8), 1206-1215. doi:10.1038/bjc.2015.327
Reuben, A., Gittelman, R., Gao, J., Zhang, J., Yusko, E. C., Wu, C. J., . . . Zhang, J. (2017). TCR Repertoire Intratumor Heterogeneity in Localized Lung Adenocarcinomas: An Association with Predicted Neoantigen Heterogeneity and Postsurgical Recurrence. Cancer Discov, 7(10), 1088-1097. doi:10.1158/2159-8290.Cd-17-0256
Ricaurte, L. M., Arrieta, O., Zatarain-Barrón, Z. L., & Cardona, A. F. (2018). Comprehensive review of fetal adenocarcinoma of the lung. Lung Cancer (Auckl), 9, 57-63. doi:10.2147/lctt.S137410
Roch, B., Coffy, A., Jean-Baptiste, S., Palaysi, E., Daures, J. P., Pujol, J. L., & Bommart, S. (2020). Cachexia - sarcopenia as a determinant of disease control rate and survival in non-small lung cancer patients receiving immune-checkpoint inhibitors. Lung Cancer, 143, 19-26. doi:10.1016/j.lungcan.2020.03.003
Rodrigues, F. S., Miranda, V. S., Carneiro-Lobo, T. C., Scalabrini, L. C., Kruspig, B., Levantini, E., . . . Bassères, D. S. (2020). IKKβ Kinase Promotes Stemness, Migration, and Invasion in KRAS-Driven Lung Adenocarcinoma Cells. Int J Mol Sci, 21(16). doi:10.3390/ijms21165806
Roncaroli, F., Chatterjee, D., Giannini, C., Pereira, M., La Rosa, S., Brouland, J. P., . . . Radotra, B. (2020). Primary papillary epithelial tumour of the sella: expanding the spectrum of TTF-1-positive sellar lesions. Neuropathol Appl Neurobiol, 46(5), 493-505. doi:10.1111/nan.12622
Sahin, I., Saat, H., Aksoy, S., Dizdar, O., Erdem, H. B., & Bahsi, T. (2022). Liquid biopsy: Novel perspectives on the importance and spectrum of PIK3CA, PTEN and RET mutations in solid tumors. Mol Clin Oncol, 16(1), 1. doi:10.3892/mco.2021.2434
Saji, H., Sakai, H., Kimura, H., Miyazawa, T., Marushima, H., & Nakamura, H. (2017). Survival significance of epidermal growth factor receptor tyrosine kinase inhibitors and current staging system for survival after recurrence in patients with completely resected lung adenocarcinoma. Onco Targets Ther, 10, 4135-4141. doi:10.2147/ott.S136569
Sakaguchi, M., Maebayashi, T., Aizawa, T., Ishibashi, N., & Saito, T. (2020). Successful treatment of nonsmall cell lung cancer patients with leptomeningeal metastases using whole brain radiotherapy and tyrosine kinase inhibitors. J Cancer Res Ther, 16(4), 930-932. doi:10.4103/jcrt.JCRT_1343_16
Sakai, A., Kasahara, K., & Sone, T. (2013). Detection of EGFR T790M Mutation in Pericardial Effusion from a Non-Small Cell Lung Cancer Patient with Erlotinib Therapy. Case Rep Oncol, 6(1), 15-20. doi:10.1159/000345947
Sakai, A., Tagami, M., Kakehashi, A., Katsuyama-Yoshikawa, A., Misawa, N., Wanibuchi, H., . . . Honda, S. (2020). Expression, intracellular localization, and mutation of EGFR in conjunctival squamous cell carcinoma and the association with prognosis and treatment. PLoS One, 15(8), e0238120. doi:10.1371/journal.pone.0238120
Sakai, H., Kimura, H., Otsubo, K., Miyazawa, T., Marushima, H., Kojima, K., . . . Saji, H. (2022). Minichromosome maintenance 2 is an independent predictor of survival in patients with lung adenocarcinoma. Mol Clin Oncol, 16(1), 22. doi:10.3892/mco.2021.2455
Sakai, T., Udagawa, H., Matsumoto, S., Yoh, K., Nosaki, K., Ikeda, T., . . . Ishii, G. (2021). Morphological, immune and genetic features in biopsy sample associated with the efficacy of pembrolizumab in patients with non-squamous non-small cell lung cancer. J Cancer Res Clin Oncol, 147(4), 1227-1237. doi:10.1007/s00432-020-03413-5
Sakanoue, I., Hamakawa, H., Kaji, R., Imai, Y., Katakami, N., & Takahashi, Y. (2018). Sleeve lobectomy for lung adenocarcinoma treated with neoadjuvant afatinib. J Thorac Dis, 10(3), E170-e174. doi:10.21037/jtd.2018.02.03
Sakatani, T., Maemura, K., Hiyama, N., Amano, Y., Watanabe, K., Kage, H., . . . Takai, D. (2017). High expression of IRE1 in lung adenocarcinoma is associated with a lower rate of recurrence. Jpn J Clin Oncol, 47(6), 543-550. doi:10.1093/jjco/hyx031
Sato, H., Sasajima, J., Okada, T., Hayashi, A., Kawabata, H., Goto, T., . . . Okumura, T. (2020). Resection for pancreatic cancer metastases contributes to survival: A case report with sequential tumor genotype profiling during the long-term postoperative course. Medicine (Baltimore), 99(25), e20564. doi:10.1097/md.0000000000020564
Sato, K., Takeyama, Y., Yoshihara, M., Kato, T., Hashimoto, H., Fukui, Y., . . . Suzuki, R. (2012). CBDCA + Pemetrexed + Bevacizumab and Its Maintenance Chemotherapy in a Case of Solitary Breast Metastasis from a Lung Adenocarcinoma Resistant to Gefitinib. Case Rep Oncol, 5(3), 546-553. doi:10.1159/000343678
Sato, Y., Fujimoto, D., Uehara, K., Shimizu, R., Ito, J., Kogo, M., . . . Tomii, K. (2016). The prognostic value of serum CA 19-9 for patients with advanced lung adenocarcinoma. BMC Cancer, 16(1), 890. doi:10.1186/s12885-016-2897-6
Sato, Y., Sekine, A., Hagiwara, E., Sato, M., Yamaya, T., Asaoka, M., . . . Ogura, T. (2021). Successful treatment with afatinib following the failure of osimertinib rechallenge with osimertinib-induced interstitial lung disease: A case report. Respir Med Case Rep, 33, 101450. doi:10.1016/j.rmcr.2021.101450
Satoh, H., Kagohashi, K., & Kurishima, K. (2013). Peritoneal relapse from lung adenocarcinoma after a response to EGFR-TKI. Tuberk Toraks, 61(4), 346-347. doi:10.5578/tt.6534
Schneider, T. C., Kapiteijn, E., van Wezel, T., Smit, J. W. A., van der Hoeven, J. J. M., & Morreau, H. (2016). (Secondary) solid tumors in thyroid cancer patients treated with the multi-kinase inhibitor sorafenib may present diagnostic challenges. BMC Cancer, 16, 31. doi:10.1186/s12885-016-2060-4
Schuster, K., Venkateswaran, N., Rabellino, A., Girard, L., Peña-Llopis, S., & Scaglioni, P. P. (2014). Nullifying the CDKN2AB locus promotes mutant K-ras lung tumorigenesis. Mol Cancer Res, 12(6), 912-923. doi:10.1158/1541-7786.Mcr-13-0620-t
Schweiger, T., Hegedüs, B., Nikolowsky, C., Hegedüs, Z., Szirtes, I., Mair, R., . . . Hoetzenecker, K. (2014). EGFR, BRAF and KRAS status in patients undergoing pulmonary metastasectomy from primary colorectal carcinoma: a prospective follow-up study. Ann Surg Oncol, 21(3), 946-954. doi:10.1245/s10434-013-3386-7
Seegobin, K., Majeed, U., Lou, Y., Zhao, Y., & Manochakian, R. (2020). Patients with high-grade alectinib-induced skin rash: How do we desensitize these patients? A case report and review of literature. SAGE Open Med Case Rep, 8, 2050313x20966895. doi:10.1177/2050313x20966895
Sengupta, S., Weyand, A. C., Upadhyaya, S. A., Wu, Y. M., Robinson, D. R., & Mody, R. J. (2019). Clinical Implications of Real-time Integrative Sequencing in Management of Patients With Suspected Germline BAP1 Mutations. J Pediatr Hematol Oncol, 41(4), e263-e265. doi:10.1097/mph.0000000000001122
Seo, J. S., Ju, Y. S., Lee, W. C., Shin, J. Y., Lee, J. K., Bleazard, T., . . . Kim, Y. T. (2012). The transcriptional landscape and mutational profile of lung adenocarcinoma. Genome Res, 22(11), 2109-2119. doi:10.1101/gr.145144.112
Sereno, M., Moreno, V., Moreno Rubio, J., Gómez-Raposo, C., García Sánchez, S., Hernández Jusdado, R., . . . Casado Sáenz, E. (2015). A significant response to sorafenib in a woman with advanced lung adenocarcinoma and a BRAF non-V600 mutation. Anticancer Drugs, 26(9), 1004-1007. doi:10.1097/cad.0000000000000277
Shao, K., Wang, Y., Xue, Q., Mu, J., Gao, Y., Wang, Y., . . . Gao, S. (2019). Clinicopathological features and prognosis of ciliated muconodular papillary tumor. J Cardiothorac Surg, 14(1), 143. doi:10.1186/s13019-019-0962-3
Sheikine, Y., Pavlick, D., Klempner, S. J., Trabucco, S. E., Chung, J. H., Rosenzweig, M., . . . Ali, S. M. (2018). BRAF in Lung Cancers: Analysis of Patient Cases Reveals Recurrent BRAF Mutations, Fusions, Kinase Duplications, and Concurrent Alterations. JCO Precis Oncol, 2. doi:10.1200/po.17.00172
Shen, L., Lin, J., Wang, B., Xu, H., Zhao, K., & Zhang, L. (2019). [Computed tomography findings, clinicopathological features, genetic characteristics and prognosis of in situ and minimally invasive lung adenocarcinomas]. Nan Fang Yi Ke Da Xue Xue Bao, 39(9), 1107-1112. doi:10.12122/j.issn.1673-4254.2019.09.17
Shen, M., Jiang, K., Sui, Y., Xu, Z., Cui, H., Wang, Y., . . . Chen, Y. (2021). Characterization of CD66b and its relationship between immune checkpoints and their synergistic impact in the prognosis of surgically resected lung adenocarcinoma. Lung Cancer, 160, 84-91. doi:10.1016/j.lungcan.2021.08.012
Shen, W., Chen, J., Gao, L., Ma, G., Yang, L., Liang, H., . . . Zhou, Q. (2021). Ureteral metastasis from pulmonary adenocarcinoma: A case report and literature review. Thorac Cancer, 12(23), 3277-3280. doi:10.1111/1759-7714.14172
Shi, R., Filho, S. N. M., Li, M., Fares, A., Weiss, J., Pham, N. A., . . . Liu, G. (2020). BRAF V600E mutation and MET amplification as resistance pathways of the second-generation anaplastic lymphoma kinase (ALK) inhibitor alectinib in lung cancer. Lung Cancer, 146, 78-85. doi:10.1016/j.lungcan.2020.05.018
Shi, X., Tan, H., Le, X., Xian, H., Li, X., Huang, K., . . . Zhang, J. (2018). An expression signature model to predict lung adenocarcinoma-specific survival. Cancer Manag Res, 10, 3717-3732. doi:10.2147/cmar.S159563
Shih, J., Bashir, B., Gustafson, K. S., Andrake, M., Dunbrack, R. L., Goldstein, L. J., & Boumber, Y. (2015). Cancer Signature Investigation: ERBB2 (HER2)-Activating Mutation and Amplification-Positive Breast Carcinoma Mimicking Lung Primary. J Natl Compr Canc Netw, 13(8), 947-952. doi:10.6004/jnccn.2015.0115
Shim, H. S., Kenudson, M., Zheng, Z., Liebers, M., Cha, Y. J., Hoang Ho, Q., . . . Iafrate, A. J. (2015). Unique Genetic and Survival Characteristics of Invasive Mucinous Adenocarcinoma of the Lung. J Thorac Oncol, 10(8), 1156-1162. doi:10.1097/jto.0000000000000579
Shimizu, K., Hirami, Y., Saisho, S., Yukawa, T., Maeda, A., Yasuda, K., & Nakata, M. (2012). Membrane-bound estrogen receptor-α expression and epidermal growth factor receptor mutation are associated with a poor prognosis in lung adenocarcinoma patients. World J Surg Oncol, 10, 141. doi:10.1186/1477-7819-10-141
Shimizu, K., Nakazawa, S., Mogi, A., & Kuwano, H. (2018). Segmentectomy of the left superior segment (S(6)) 4 years after segmentectomy of the left dorsobasal segment (S(10)). J Thorac Cardiovasc Surg, 155(3), 1302-1304. doi:10.1016/j.jtcvs.2017.08.119
Shintani, H., Oura, S., Yamaguchi, T., & Makimoto, S. (2021). Bone Metastasis of Non-Small-Cell Lung Cancer Showing Pathological Complete Response to Osimertinib Monotherapy. Case Rep Oncol, 14(3), 1876-1881. doi:10.1159/000520473
Shiono, S., & Yanagawa, N. (2016). Spread through air spaces is a predictive factor of recurrence and a prognostic factor in stage I lung adenocarcinoma. Interact Cardiovasc Thorac Surg, 23(4), 567-572. doi:10.1093/icvts/ivw211
Shiroyama, T., Tamiya, M., Hayama, M., Nishihara, T., Nishida, T., Tanaka, A., . . . Hirashima, T. (2016). A heterochronic genetic change from an EGFR mutation to an ALK rearrangement in a patient with lung adenocarcinoma: a case report. J Thorac Dis, 8(5), E345-348. doi:10.21037/jtd.2016.03.43
Sivakumar, S., San Lucas, F. A., Jakubek, Y. A., McDowell, T. L., Lang, W., Kallsen, N., . . . Scheet, P. (2019). Genomic landscape of allelic imbalance in premalignant atypical adenomatous hyperplasias of the lung. EBioMedicine, 42, 296-303. doi:10.1016/j.ebiom.2019.03.020
Somoza, A. D., & Aly, F. Z. (2014). Utility of molecular tests in cytopathology. Cytojournal, 11, 5. doi:10.4103/1742-6413.129183
Song, Q., Shang, J., Yang, Z., Zhang, L., Zhang, C., Chen, J., & Wu, X. (2019). Identification of an immune signature predicting prognosis risk of patients in lung adenocarcinoma. J Transl Med, 17(1), 70. doi:10.1186/s12967-019-1824-4
Song, W., Di, S., Liu, J., Fan, B., Zhao, J., Zhou, S., . . . Gong, T. (2020). Salvage surgery for advanced non-small cell lung cancer after targeted therapy: A case series. Thorac Cancer, 11(4), 1061-1067. doi:10.1111/1759-7714.13366
Song, Y., Chen, D., Zhang, X., Luo, Y., & Li, S. (2019). Integrating genetic mutations and expression profiles for survival prediction of lung adenocarcinoma. Thorac Cancer, 10(5), 1220-1228. doi:10.1111/1759-7714.13072
Song, Z., Su, H., & Zhang, Y. (2016). Patients with ROS1 rearrangement-positive non-small-cell lung cancer benefit from pemetrexed-based chemotherapy. Cancer Med, 5(10), 2688-2693. doi:10.1002/cam4.809
Song, Z., Yu, X., & Zhang, Y. (2016). Mutation and prognostic analyses of PIK3CA in patients with completely resected lung adenocarcinoma. Cancer Med, 5(10), 2694-2700. doi:10.1002/cam4.852
Sonobe, M., Kobayashi, M., Ishikawa, M., Kikuchi, R., Nakayama, E., Takahashi, T., . . . Date, H. (2012). Impact of KRAS and EGFR gene mutations on recurrence and survival in patients with surgically resected lung adenocarcinomas. Ann Surg Oncol, 19 Suppl 3, S347-354. doi:10.1245/s10434-011-1799-8
Sonoda, D., Matsuura, Y., Kondo, Y., Ichinose, J., Nakao, M., Ninomiya, H., . . . Mun, M. (2022). A Reasonable Definition of Oligo-Recurrence in Non-Small-Cell Lung Cancer. Clin Lung Cancer, 23(1), 82-90. doi:10.1016/j.cllc.2021.10.013
Sonzogni, A., Bianchi, F., Fabbri, A., Cossa, M., Rossi, G., Cavazza, A., . . . Pelosi, G. (2017). Pulmonary adenocarcinoma with mucin production modulates phenotype according to common genetic traits: a reappraisal of mucinous adenocarcinoma and colloid adenocarcinoma. J Pathol Clin Res, 3(2), 139-152. doi:10.1002/cjp2.67
Spans, L., Fletcher, C. D., Antonescu, C. R., Rouquette, A., Coindre, J. M., Sciot, R., & Debiec-Rychter, M. (2016). Recurrent MALAT1-GLI1 oncogenic fusion and GLI1 up-regulation define a subset of plexiform fibromyxoma. J Pathol, 239(3), 335-343. doi:10.1002/path.4730
Staaf, J., Isaksson, S., Karlsson, A., Jönsson, M., Johansson, L., Jönsson, P., . . . Planck, M. (2013). Landscape of somatic allelic imbalances and copy number alterations in human lung carcinoma. Int J Cancer, 132(9), 2020-2031. doi:10.1002/ijc.27879
Stahl, J. M., Walther, Z., Chang, B. W., Hochster, H. S., & Johung, K. L. (2017). A Long-Term Survivor of Metastatic Pancreatic Adenocarcinoma: Free of Recurrence 12 Years After Treatment of Oligometastatic Disease. Cureus, 9(2), e1007. doi:10.7759/cureus.1007
Suda, K., Ohara, S., Fujino, T., Hamada, A., Chiba, M., Shimoji, M., . . . Mitsudomi, T. (2022). Frequent EGFR Mutations and Better Prognosis in Positron Emission Tomography-Negative, Solid-Type Lung Cancer. Clin Lung Cancer, 23(1), e60-e68. doi:10.1016/j.cllc.2021.10.003
Suh, Y. J., Lee, H. J., Kim, Y. T., Kang, C. H., Park, I. K., Jeon, Y. K., & Chung, D. H. (2018). Added prognostic value of CT characteristics and IASLC/ATS/ERS histologic subtype in surgically resected lung adenocarcinomas. Lung Cancer, 120, 130-136. doi:10.1016/j.lungcan.2018.04.007
Sukrithan, V., Barbaro, A., Chergui, A., Ko, B., Lin, J., Cheng, H., & Goel, S. (2020). Differential Efficacy of Anti-VEGF Antibodies Based on Sex and Race in a Diverse Cohort of Advanced Nonsquamous Non-Small Cell Lung Cancer. Am J Clin Oncol, 43(1), 64-68. doi:10.1097/coc.0000000000000628
Sumiyoshi, S., Yoshizawa, A., Sonobe, M., Kobayashi, M., Fujimoto, M., Tsuruyama, T., . . . Haga, H. (2013). Pulmonary adenocarcinomas with micropapillary component significantly correlate with recurrence, but can be well controlled with EGFR tyrosine kinase inhibitors in the early stages. Lung Cancer, 81(1), 53-59. doi:10.1016/j.lungcan.2013.04.003
Sun, D., Chen, J., Liu, L., Zhao, G., Dong, P., Wu, B., . . . Dong, L. (2018). Establishment of a 12-gene expression signature to predict colon cancer prognosis. PeerJ, 6, e4942. doi:10.7717/peerj.4942
Sun, J. M., Lira, M., Pandya, K., Choi, Y. L., Ahn, J. S., Mao, M., . . . Kim, J. (2014). Clinical characteristics associated with ALK rearrangements in never-smokers with pulmonary adenocarcinoma. Lung Cancer, 83(2), 259-264. doi:10.1016/j.lungcan.2013.11.009
Sun, L., Li, B., Wang, B., Li, J., & Li, J. (2022). Afatinib in the treatment of brain metastases of lung cancer with one rare EGFR mutation: a two-case report. Anticancer Drugs, 33(1), 112-118. doi:10.1097/cad.0000000000001138
Sun, P. L., Liu, J. N., Cao, L. Q., Yao, M., & Gao, H. W. (2017). [To evaluate the clinicopathologic characteristics and outcome of tumor cells spreading through air spaces in patients with adenocarcinoma of lung]. Zhonghua Bing Li Xue Za Zhi, 46(5), 303-308. doi:10.3760/cma.j.issn.0529-5807.2017.05.004
Sun, X., Li, K., Zhao, R., Sun, Y., Xu, J., Peng, Z. Y., . . . Tang, S. C. (2021). Lung cancer pathogenesis and poor response to therapy were dependent on driver oncogenic mutations. Life Sci, 265, 118797. doi:10.1016/j.lfs.2020.118797
Sun, Y., Chen, C., Zhang, P., Xie, H., Hou, L., Hui, Z., . . . Gao, W. (2014). Reduced miR-3127-5p expression promotes NSCLC proliferation/invasion and contributes to dasatinib sensitivity via the c-Abl/Ras/ERK pathway. Sci Rep, 4, 6527. doi:10.1038/srep06527
Sun, Z., Wang, L., Eckloff, B. W., Deng, B., Wang, Y., Wampfler, J. A., . . . Yang, P. (2014). Conserved recurrent gene mutations correlate with pathway deregulation and clinical outcomes of lung adenocarcinoma in never-smokers. BMC Med Genomics, 7, 32. doi:10.1186/1755-8794-7-32
Sun, Z., Wang, L., Eckloff, B. W., Deng, B., Wang, Y., Wampfler, J. A., . . . Yang, P. (2017). Erratum to: Conserved recurrent gene mutations correlate with pathway deregulation and clinical outcomes of lung adenocarcinoma in never-smokers. BMC Med Genomics, 10(1), 1. doi:10.1186/s12920-016-0237-y
Suzuki, M., Ikeda, K., Shiraishi, K., Eguchi, A., Mori, T., Yoshimoto, K., . . . Baba, H. (2014). Aberrant methylation and silencing of IRF8 expression in non-small cell lung cancer. Oncol Lett, 8(3), 1025-1030. doi:10.3892/ol.2014.2234
Suzuki, S., Sakurai, H., Masai, K., Asakura, K., Nakagawa, K., Motoi, N., & Watanabe, S. I. (2017). A Proposal for Definition of Minimally Invasive Adenocarcinoma of the Lung Regardless of Tumor Size. Ann Thorac Surg, 104(3), 1027-1032. doi:10.1016/j.athoracsur.2017.02.067
Tabbò, F., Nottegar, A., Guerrera, F., Migliore, E., Luchini, C., Maletta, F., . . . Chilosi, M. (2018). Cell of origin markers identify different prognostic subgroups of lung adenocarcinoma. Hum Pathol, 75, 167-178. doi:10.1016/j.humpath.2018.01.017
Tai, M. L., Tan, E. C., Ang, C. C., & Liam, C. K. (2016). Recurrent cerebral infarcts secondary to marantic endocarditis in a patient with adenocarcinoma of the lung. Singapore Med J, 57(9), 524-525. doi:10.11622/smedj.2016157
Taieb, J., Balogoun, R., Le Malicot, K., Tabernero, J., Mini, E., Folprecht, G., . . . Laurent-Puig, P. (2017). Adjuvant FOLFOX +/- cetuximab in full RAS and BRAF wildtype stage III colon cancer patients. Ann Oncol, 28(4), 824-830. doi:10.1093/annonc/mdw687
Takamochi, K., Hara, K., Hayashi, T., Kohsaka, S., Takahashi, F., Suehara, Y., & Suzuki, K. (2021). Programmed death-ligand 1 expression and its associations with clinicopathological features, prognosis, and driver oncogene alterations in surgically resected lung adenocarcinoma. Lung Cancer, 161, 163-170. doi:10.1016/j.lungcan.2021.09.011
Takamochi, K., Oh, S., Matsunaga, T., & Suzuki, K. (2017). Prognostic impacts of EGFR mutation status and subtype in patients with surgically resected lung adenocarcinoma. J Thorac Cardiovasc Surg, 154(5), 1768-1774.e1761. doi:10.1016/j.jtcvs.2017.06.062
Takamori, S., Matsubara, T., Haratake, N., Toyokawa, G., Fujishita, T., Toyozawa, R., . . . Seto, T. (2021). Targeted Therapy for RET Fusion Lung Cancer: Breakthrough and Unresolved Issue. Front Oncol, 11, 704084. doi:10.3389/fonc.2021.704084
Takanashi, Y., Tajima, S., Hayakawa, T., Takahashi, T., Neyatani, H., & Funai, K. (2016). Pulmonary micropapillary-type adenosquamous carcinoma sharing epidermal growth factor receptor mutation in adenocarcinoma and squamous cell carcinoma. Respirol Case Rep, 4(5), e00179. doi:10.1002/rcr2.179
Takeda, M., Sakai, K., Terashima, M., Kaneda, H., Hayashi, H., Tanaka, K., . . . Nishio, K. (2015). Clinical application of amplicon-based next-generation sequencing to therapeutic decision making in lung cancer. Ann Oncol, 26(12), 2477-2482. doi:10.1093/annonc/mdv475
Takeda-Miyata, N., Miyagawa-Hayashino, A., Hamada, S., Nagamine, M., Fujii, T., Imura, T., . . . Konishi, E. (2022). A clinicopathologic and molecular analysis of five cases of bronchiolar adenoma with rare mutations. Pathol Int. doi:10.1111/pin.13213
Tamura, K., Nukiwa, T., Gemma, A., Yamamoto, N., Mizushima, M., Ochai, K., . . . Nakanishi, Y. (2019). Real-world treatment of over 1600 Japanese patients with EGFR mutation-positive non-small cell lung cancer with daily afatinib. Int J Clin Oncol, 24(8), 917-926. doi:10.1007/s10147-019-01439-5
Tamura, T., Kawakado, K., Makimoto, G., Nakanishi, M., & Kuyama, S. (2021). Limited effect of afatinib in a non-small cell lung cancer patient harboring an epidermal growth factor receptor K860I missense mutation: A case report. Thorac Cancer, 12(11), 1770-1774. doi:10.1111/1759-7714.13941
Tamura, Y., Fujiwara, Y., Yamamoto, N., Nokihara, H., Horinouchi, H., Kanda, S., . . . Ohe, Y. (2015). Retrospective analysis of the efficacy of chemotherapy and molecular targeted therapy for advanced pulmonary pleomorphic carcinoma. BMC Res Notes, 8, 800. doi:10.1186/s13104-015-1762-z
Tan, Q., Cui, J., Huang, J., Ding, Z., Lin, H., Niu, X., . . . Lu, S. (2016). Genomic Alteration During Metastasis of Lung Adenocarcinoma. Cell Physiol Biochem, 38(2), 469-486. doi:10.1159/000438644
Tanaka, H., Okamoto, T., Shinyama, M., Matsumura, S., & Fujii, T. (2013). [A case of lung adenocarcinoma wtih exon19 and T790M mutations in EGFR having good response to erlotinib after gefitinib treatment failure]. Gan To Kagaku Ryoho, 40(8), 1067-1069. 
Tanaka, K., Hida, T., Oya, Y., Oguri, T., Yoshida, T., Shimizu, J., . . . Yatabe, Y. (2015). EGFR Mutation Impact on Definitive Concurrent Chemoradiation Therapy for Inoperable Stage III Adenocarcinoma. J Thorac Oncol, 10(12), 1720-1725. doi:10.1097/jto.0000000000000675
Tanaka, K., Shimizu, K., Kakegawa, S., Ohtaki, Y., Nagashima, T., Kaira, K., . . . Takeyoshi, I. (2016). Prognostic significance of aromatase and estrogen receptor beta expression in EGFR wild-type lung adenocarcinoma. Am J Transl Res, 8(1), 81-97. 
Tang, H., Wang, H., Xi, S., He, C., Chang, Y., Wang, Q., & Wu, Y. (2018). Perioperative chemotherapy with pemetrexed and cisplatin for pulmonary large-cell neuroendocrine carcinoma: a case report and literature review. Onco Targets Ther, 11, 2557-2563. doi:10.2147/ott.S160565
Tang, H., Wang, S., Xiao, G., Schiller, J., Papadimitrakopoulou, V., Minna, J., . . . Xie, Y. (2017). Comprehensive evaluation of published gene expression prognostic signatures for biomarker-based lung cancer clinical studies. Ann Oncol, 28(4), 733-740. doi:10.1093/annonc/mdw683
Taniguchi, H., Yamada, T., Wang, R., Tanimura, K., Adachi, Y., Nishiyama, A., . . . Yano, S. (2019). AXL confers intrinsic resistance to osimertinib and advances the emergence of tolerant cells. Nat Commun, 10(1), 259. doi:10.1038/s41467-018-08074-0
Tao, C. W., Chen, M. Y., Tseng, C. M., Lapke, N., Chen, S. J., & Tan, K. T. (2020). Advanced Lung Adenocarcinoma Patient with ERBB2 Amplification Identified by Comprehensive Genomic Profiling Benefits from Trastuzumab. Case Rep Oncol Med, 2020, 9072173. doi:10.1155/2020/9072173
Testa, U., Castelli, G., & Pelosi, E. (2018). Lung Cancers: Molecular Characterization, Clonal Heterogeneity and Evolution, and Cancer Stem Cells. Cancers (Basel), 10(8). doi:10.3390/cancers10080248
Thomas, A., Xi, L., Carter, C. A., Rajan, A., Khozin, S., Szabo, E., . . . Raffeld, M. (2013). Concurrent molecular alterations in tumors with germ line epidermal growth factor receptor T790M mutations. Clin Lung Cancer, 14(4), 452-456. doi:10.1016/j.cllc.2013.01.005
Tian, W. J., Liu, S. S., & Li, B. R. (2020). The Combined Detection of Immune Genes for Predicting the Prognosis of Patients With Non-Small Cell Lung Cancer. Technol Cancer Res Treat, 19, 1533033820977504. doi:10.1177/1533033820977504
Tian, Y., Feng, J., Jiang, L., Ning, J., Gu, Z., Huang, J., & Luo, Q. (2021). Integration of clinicopathological and mutational data offers insight into lung cancer with tumor spread through air spaces. Ann Transl Med, 9(12), 985. doi:10.21037/atm-21-2256
Tímár, J., Lotz, G., Rásó, E., & Moldvay, J. (2017). [Molecular diagnostics of ALK-positive lung cancer]. Magy Onkol, 61(3), 301-311. 
Timsah, Z., Berrout, J., Suraokar, M., Behrens, C., Song, J., Lee, J. J., . . . Ladbury, J. E. (2015). Expression pattern of FGFR2, Grb2 and Plcγ1 acts as a novel prognostic marker of recurrence recurrence-free survival in lung adenocarcinoma. Am J Cancer Res, 5(10), 3135-3148. 
Toba, H., Sakiyama, S., Takizawa, H., & Tangoku, A. (2016). Safe and successful treatment with afatinib in three postoperative non-small cell lung cancer patients with recurrences following gefitinib/erlotinib-induced hepatotoxicity. J Med Invest, 63(1-2), 149-151. doi:10.2152/jmi.63.149
Togashi, Y., Masago, K., Hamatani, Y., Sakamori, Y., Nagai, H., Kim, Y. H., & Mishima, M. (2012). Successful erlotinib rechallenge for leptomeningeal metastases of lung adenocarcinoma after erlotinib-induced interstitial lung disease: a case report and review of the literature. Lung Cancer, 77(2), 464-468. doi:10.1016/j.lungcan.2012.04.013
Toyokawa, G., Yamada, Y., Tagawa, T., Kozuma, Y., Matsubara, T., Haratake, N., . . . Maehara, Y. (2018). Significance of Spread Through Air Spaces in Resected Pathological Stage I Lung Adenocarcinoma. Ann Thorac Surg, 105(6), 1655-1663. doi:10.1016/j.athoracsur.2018.01.037
Tsai, L. H., Chen, P. M., Cheng, Y. W., Chen, C. Y., Sheu, G. T., Wu, T. C., & Lee, H. (2014). LKB1 loss by alteration of the NKX2-1/p53 pathway promotes tumor malignancy and predicts poor survival and relapse in lung adenocarcinomas. Oncogene, 33(29), 3851-3860. doi:10.1038/onc.2013.353
Tseng, J. S., Hsu, K. H., Zheng, Z. R., Yang, T. Y., Chen, K. C., Huang, Y. H., . . . Chang, G. C. (2021). Primary Tumor Resection Is Associated with a Better Outcome among Advanced EGFR-Mutant Lung Adenocarcinoma Patients Receiving EGFR-TKI Treatment. Oncology, 99(1), 32-40. doi:10.1159/000509664
Tsunezuka, Y., Tanaka, N., Fujimori, H., Togashi, Y., Baba, S., Takeuchi, K., . . . Yano, S. (2017). The case of double primary lung adenocarcinomas with an EGFR mutation and ALK translocation successfully treated with alectinib at the post-surgical recurrence. J Med Invest, 64(3.4), 305-307. doi:10.2152/jmi.64.305
Tsutani, Y., Ito, M., Shimada, Y., Ito, H., Ikeda, N., Nakayama, H., & Okada, M. (2022). The impact of epidermal growth factor receptor mutation status on adjuvant chemotherapy for patients with high-risk stage I lung adenocarcinoma. J Thorac Cardiovasc Surg. doi:10.1016/j.jtcvs.2022.01.025
Tsutsumi, H., Yoneshima, Y., Ota, K., Otsubo, K., Iwama, E., Inoue, H., . . . Okamoto, I. (2020). Multiclonality and Radiosensitivity of Granulocyte-colony Stimulating Factor-Producing Lung Adenocarcinoma Positive for an Activating EGFR Mutation. Clin Lung Cancer, 21(1), e21-e24. doi:10.1016/j.cllc.2019.09.001
Ueda, D., Ito, M., Tsutani, Y., Giménez-Capitán, A., Román-Lladó, R., Pérez-Rosado, A., . . . Okada, M. (2021). Comprehensive analysis of the clinicopathological features, targetable profile, and prognosis of mucinous adenocarcinoma of the lung. J Cancer Res Clin Oncol, 147(12), 3709-3718. doi:10.1007/s00432-021-03609-3
Ueda, M., Namba, M., Tokumo, K., Senoo, T., Okamoto, W., Yamauchi, M., . . . Sugiyama, K. (2021). Conversion from Positive to Negative EGFR Mutation due to Clonal Selection during Long-Term Treatment with Epidermal Growth Factor Receptor-Tyrosine Kinase Inhibitors: A Case Report. Case Rep Oncol, 14(3), 1447-1453. doi:10.1159/000518246
Uozu, S., Imaizumi, K., Yamaguchi, T., Goto, Y., Kawada, K., Minezawa, T., . . . Hasegawa, Y. (2017). Feasibility of tissue re-biopsy in non-small cell lung cancers resistant to previous epidermal growth factor receptor tyrosine kinase inhibitor therapies. BMC Pulm Med, 17(1), 175. doi:10.1186/s12890-017-0514-3
van Riel, S., Thunnissen, E., Heideman, D., Smit, E. F., & Biesma, B. (2012). A patient with simultaneously appearing adenocarcinoma and small-cell lung carcinoma harbouring an identical EGFR exon 19 mutation. Ann Oncol, 23(12), 3188-3189. doi:10.1093/annonc/mds525
Vaz, D., Conde, S., Tente, D., Machado, J. C., & Barroso, A. (2017). Role of epidermal growth factor mutational status for distinction between recurrent lung cancer and second primary lung cancer: case report. Clin Respir J, 11(6), 854-858. doi:10.1111/crj.12427
Verma, A., Chopra, A., Lee, Y. W., Bharwani, L. D., Asmat, A. B., Aneez, D. B., . . . Abisheganaden, J. (2016). Can EGFR-Tyrosine Kinase Inhibitors (TKI) Alone Without Talc Pleurodesis Prevent Recurrence of Malignant Pleural Effusion (MPE) in Lung Adenocarcinoma. Curr Drug Discov Technol, 13(2), 68-76. doi:10.2174/1570163813666160524142846
Villaruz, L. C., & Socinski, M. A. (2014). Temsirolimus therapy in a patient with lung adenocarcinoma harboring an FBXW7 mutation. Lung Cancer, 83(2), 300-301. doi:10.1016/j.lungcan.2013.11.018
Villaruz, L. C., Socinski, M. A., Cunningham, D. E., Chiosea, S. I., Burns, T. F., Siegfried, J. M., & Dacic, S. (2013). The prognostic and predictive value of KRAS oncogene substitutions in lung adenocarcinoma. Cancer, 119(12), 2268-2274. doi:10.1002/cncr.28039
Vin Chang, G., Xie, C., Shahid, M., Draine, J., Alpers, J., Reindl, B., & McHale, M. (2021). Bilateral Breast Radiation Associated Angiosarcoma After Radiotherapy for Bilateral Invasive Ductal Adenocarcinoma. S D Med, 74(6), 260-263. 
Vitellius, C., Griveaux, O., Morvant, B., Pedrono, E., Venara, A., Ingster, O., . . . Caroli-Bosc, F. X. (2020). Impact of Driver Mutations on the Evolution of Isolated Metachronous Lung Metastasis of Pancreatic Ductal adenocarcinoma. Mol Diagn Ther, 24(4), 443-449. doi:10.1007/s40291-020-00472-9
Volckmar, A. L., Endris, V., Bozorgmehr, F., Lier, C., Porcel, C., Kirchner, M., . . . Stenzinger, A. (2016). Next-generation sequencing facilitates detection of the classic E13-A20 EML4-ALK fusion in an ALK-FISH/IHC inconclusive biopsy of a stage IV lung cancer patient: a case report. Diagn Pathol, 11(1), 133. doi:10.1186/s13000-016-0581-4
Wang, B., & Jin, H. (2022). A case report of low grade fetal lung adenocarcinoma with TP53 mutation. Medicine (Baltimore), 101(11). doi:10.1097/md.0000000000029047
Wang, F., Xie, X., Song, M., Ji, L., Liu, M., Li, P., . . . Zhou, C. (2020). Tumor immune microenvironment and mutational analysis of tracheal adenoid cystic carcinoma. Ann Transl Med, 8(12), 750. doi:10.21037/atm-20-3433
Wang, H., Wei, C., Pan, P., Yuan, F., & Cheng, J. (2021). Identification of a methylomics-associated nomogram for predicting overall survival of stage I-II lung adenocarcinoma. Sci Rep, 11(1), 9938. doi:10.1038/s41598-021-89429-4
Wang, J., Nong, J., Jia, H., Qin, N., Li, X., Zhang, H., . . . Zhang, S. (2014). Efficacy and predictors of EGFR tyrosine kinase inhibitors in Chinese advanced lung adenocarcinoma: analyses of 253 cases from a single institute. Oncol Res, 21(5), 237-246. doi:10.3727/096504014x13907540404833
Wang, J. L., Fang, C. L., Tzeng, Y. T., Hsu, H. L., Lin, S. E., Yu, M. C., . . . Liu, H. E. (2018). Prognostic value of localization of epidermal growth factor receptor in lung adenocarcinoma. J Biomed Sci, 25(1), 53. doi:10.1186/s12929-018-0451-3
Wang, R., Zhang, Y., Pan, Y., Li, Y., Hu, H., Cai, D., . . . Chen, H. (2015). Comprehensive investigation of oncogenic driver mutations in Chinese non-small cell lung cancer patients. Oncotarget, 6(33), 34300-34308. doi:10.18632/oncotarget.5549
Wang, W. P., Wang, K. N., Gao, Q., & Chen, L. Q. (2012). Lack of EGFR mutations benefiting gefitinib treatment in adenocarcinoma of esophagogastric junction. World J Surg Oncol, 10, 14. doi:10.1186/1477-7819-10-14
Wang, X., Huang, L., Cai, J., & Liu, A. (2021). A Novel KIF5B-EGFR Fusion Variant in Non-Small-Cell Lung Cancer and Response to Afatinib: A Case Report. Onco Targets Ther, 14, 3739-3744. doi:10.2147/ott.S313896
Wang, X., Min, S., Liu, H., Wu, N., Liu, X., Wang, T., . . . Chen, Y. (2019). Nf1 loss promotes Kras-driven lung adenocarcinoma and results in Psat1-mediated glutamate dependence. EMBO Mol Med, 11(6). doi:10.15252/emmm.201809856
Wang, X., Peng, W., Zeng, Z., Cai, J., & Liu, A. (2021). Emerging a Novel VOPP1-EGFR Fusion Coexistent With T790M as an Acquired Resistance Mechanism to Prior Icotinib and Sensitive to Osimertinib in a Patient With EGFR L858R Lung Adenocarcinoma: A Case Report. Front Oncol, 11, 720819. doi:10.3389/fonc.2021.720819
Wang, Y., Xu, Y., Wang, X., Sun, C., Guo, Y., Shao, G., . . . Ma, K. (2019). RET fusion in advanced non-small-cell lung cancer and response to cabozantinib: A case report. Medicine (Baltimore), 98(3), e14120. doi:10.1097/md.0000000000014120
Wang, Y., Yang, X., Tian, X., Jia, Z., Bing, Z., Cao, L., . . . Liang, N. (2020). Neoadjuvant immunotherapy plus chemotherapy achieved pathologic complete response in stage IIIB lung adenocarcinoma harbored EGFR G779F: a case report. Ann Palliat Med, 9(6), 4339-4345. doi:10.21037/apm-20-1692
Warth, A., Muley, T., Kossakowski, C., Stenzinger, A., Schirmacher, P., Dienemann, H., & Weichert, W. (2015). Prognostic impact and clinicopathological correlations of the cribriform pattern in pulmonary adenocarcinoma. J Thorac Oncol, 10(4), 638-644. doi:10.1097/jto.0000000000000490
Watanabe, A., Inada, R., Nagasaka, T., Yagi, T., Matsumoto, H., Toshima, T., . . . Fujiwara, T. (2015). [A successful multimodality therapy for a case of recurrent rectal cancer with KRAS mutation]. Gan To Kagaku Ryoho, 42(2), 237-239. 
Watanabe, H., Saito, H., Yokose, T., Sakuma, Y., Murakami, S., Kondo, T., . . . Iwazaki, M. (2015). Relation between thin-section computed tomography and clinical findings of mucinous adenocarcinoma. Ann Thorac Surg, 99(3), 975-981. doi:10.1016/j.athoracsur.2014.10.065
Watanabe, K., Haratake, N., Takenaka, T., Nagano, T., Oku, Y., Kosai, K., . . . Oda, Y. (2021). Long-term complete response to gefitinib after treatment termination in a patient with recurrent post-operative EGFR-mutated lung adenocarcinoma: case report and literature review. Transl Cancer Res, 10(11), 5010-5013. doi:10.21037/tcr-21-1140
Watanabe, K., Yasumoto, A., Amano, Y., Kage, H., Goto, Y., Yatomi, Y., . . . Nagase, T. (2018). Mean platelet volume and lymphocyte-to-monocyte ratio are associated with shorter progression-free survival in EGFR-mutant lung adenocarcinoma treated by EGFR tyrosine kinase inhibitor. PLoS One, 13(9), e0203625. doi:10.1371/journal.pone.0203625
Watanabe, M., Yokose, T., Tetsukan, W., Imai, K., Tsuboi, M., Ito, H., . . . Fujino, S. (2013). Micropapillary components in a lung adenocarcinoma predict stump recurrence 8 years after resection: a case report. Lung Cancer, 80(2), 230-233. doi:10.1016/j.lungcan.2013.01.011
Wei, X. W., Gao, X., Zhang, X. C., Yang, J. J., Chen, Z. H., Wu, Y. L., & Zhou, Q. (2020). Mutational landscape and characteristics of ERBB2 in non-small cell lung cancer. Thorac Cancer, 11(6), 1512-1521. doi:10.1111/1759-7714.13419
Weichert, W., & Warth, A. (2014). Early lung cancer with lepidic pattern: adenocarcinoma in situ, minimally invasive adenocarcinoma, and lepidic predominant adenocarcinoma. Curr Opin Pulm Med, 20(4), 309-316. doi:10.1097/mcp.0000000000000065
Woo, C. G., Son, S. M., Lee, H. C., Han, H. S., Lee, K. H., Kim, D., . . . Lee, O. J. (2021). Histologic Changes in Non-Small Cell Lung Cancer Under Various Treatments: A Comparison of Histology and Mutation Status in Serial Samples. Cancer Res Treat. doi:10.4143/crt.2021.773
Woodard, G. A., Kratz, J. R., Haro, G., Gubens, M. A., Blakely, C. M., Jones, K. D., . . . Jablons, D. M. (2021). Molecular Risk Stratification is Independent of EGFR Mutation Status in Identifying Early-Stage Non-Squamous Non-Small Cell Lung Cancer Patients at Risk for Recurrence and Likely to Benefit From Adjuvant Chemotherapy. Clin Lung Cancer, 22(6), 587-595. doi:10.1016/j.cllc.2021.08.008
Wu, D. W., Tsai, L. H., Chen, P. M., Lee, M. C., Wang, L., Chen, C. Y., . . . Lee, H. (2012). Loss of TIMP-3 promotes tumor invasion via elevated IL-6 production and predicts poor survival and relapse in HPV-infected non-small cell lung cancer. Am J Pathol, 181(5), 1796-1806. doi:10.1016/j.ajpath.2012.07.032
Wu, D. W., Wu, T. C., Chen, C. Y., & Lee, H. (2016). PAK1 Is a Novel Therapeutic Target in Tyrosine Kinase Inhibitor-Resistant Lung Adenocarcinoma Activated by the PI3K/AKT Signaling Regardless of EGFR Mutation. Clin Cancer Res, 22(21), 5370-5382. doi:10.1158/1078-0432.Ccr-15-2724
Wu, H. H., Chu, Y. C., Wang, L., Tsai, L. H., Lee, M. C., Chen, C. Y., . . . Lee, H. (2013). Cytoplasmic Ape1 expression elevated by p53 aberration may predict survival and relapse in resected non-small cell lung cancer. Ann Surg Oncol, 20 Suppl 3, S336-347. doi:10.1245/s10434-012-2431-2
Wu, L., Zhong, W., Li, A., Qiu, Z., Xie, R., Shi, H., & Lu, S. (2021). Successful treatment of EGFR T790M-mutant non-small cell lung cancer with almonertinib after osimertinib-induced interstitial lung disease: a case report and literature review. Ann Transl Med, 9(11), 950. doi:10.21037/atm-21-2823
Wu, N., Liu, S., Li, J., Hu, Z., Yan, S., Duan, H., . . . Lu, X. (2021). Deep sequencing reveals the genomic characteristics of lung adenocarcinoma presenting as ground-glass nodules (GGNs). Transl Lung Cancer Res, 10(3), 1239-1255. doi:10.21037/tlcr-20-1086
Wu, Y., Zhu, Z., Chen, Y., & Chai, Y. (2017). Tonsillar metastasis of nonsmall cell lung cancer with G719S mutation in exon 18: A case report. Medicine (Baltimore), 96(49), e9003. doi:10.1097/md.0000000000009003
Xiang, C., Zhang, W., Xiong, L. W., Cai, X. W., Teng, H. H., Zhao, R. Y., . . . Han, Y. C. (2021). EGFR Thr790Leu as a Potential Resistance Mechanism to First-Generation EGFR Tyrosine Kinase Inhibitor May Respond to Osimertinib in Patients With Lung Adenocarcinoma. JTO Clin Res Rep, 2(7), 100185. doi:10.1016/j.jtocrr.2021.100185
Xiao, Z. J., Liu, J., Wang, S. Q., Zhu, Y., Gao, X. Y., Tin, V. P., . . . Wong, M. P. (2017). NFATc2 enhances tumor-initiating phenotypes through the NFATc2/SOX2/ALDH axis in lung adenocarcinoma. Elife, 6. doi:10.7554/eLife.26733
Xing, P., Li, J., Shi, Y., & Zhang, X. (2014). Recurrent response to advanced lung adenocarcinoma with erlotinib developing leptomeningeal metastases during gefitinib therapy and two case reports. Thorac Cancer, 5(1), 38-42. doi:10.1111/1759-7714.12049
Xu, F., Yang, G., Xu, H., Yang, L., Qiu, W., & Wang, Y. (2020). Treatment outcome and clinical characteristics of HER2 mutated advanced non-small cell lung cancer patients in China. Thorac Cancer, 11(3), 679-685. doi:10.1111/1759-7714.13317
Xu, J., Liu, X., Yang, S., & Shi, Y. (2020). Apatinib Monotherapy or Combination Therapy for Non-Small Cell Lung Cancer Patients With Brain Metastases. Oncol Res, 28(2), 127-133. doi:10.3727/096504019x15707896762251
Xu, S. B., Xie, M. R., Li, C. W., Wu, X. N., & Xu, M. Q. (2019). Correlation of pulmonary venous circulating tumor cells with clinicopathological parameters in patients with early-stage lung adenocarcinoma. Transl Cancer Res, 8(3), 887-898. doi:10.21037/tcr.2019.05.19
Xu, Y., Li, J., Tong, B., Chen, M., Liu, X., Zhong, W., . . . Wang, M. (2020). Positive tumour CD47 expression is an independent prognostic factor for recurrence in resected non-small cell lung cancer. ESMO Open, 5(4). doi:10.1136/esmoopen-2020-000823
Xu, Y., Zheng, M., Wang, N., & Wang, R. (2019). Comprehensive Study of Surgical Treated Lung Adenocarcinoma with Ground Glass Nodule Component. Med Sci Monit, 25, 8492-8498. doi:10.12659/msm.919532
Xu, Y., Zhu, C., Qian, W., & Zheng, M. (2017). Comprehensive study of mutational and clinicopathologic characteristics of adenocarcinoma with lepidic pattern in surgical resected lung adenocarcinoma. J Cancer Res Clin Oncol, 143(1), 181-186. doi:10.1007/s00432-016-2255-8
Xue, D., Lin, H., Lin, L., Wei, Q., Yang, S., & Chen, X. (2021). TTN/TP53 mutation might act as the predictor for chemotherapy response in lung adenocarcinoma and lung squamous carcinoma patients. Transl Cancer Res, 10(3), 1284-1294. doi:10.21037/tcr-20-2568
Yabe, N., Masuda, M., Tamura, E., Morishige, S., Saito, A., Harada, Y., . . . Murai, S. (2018). [A Case of Complete Response to Computed Tomography-Guided Celiac Plexus Neurolysis of Pain Associated with Postoperative Recurrence of Colon Cancer]. Gan To Kagaku Ryoho, 45(13), 1877-1879. 
Yagishita, S., Horinouchi, H., Katsui Taniyama, T., Nakamichi, S., Kitazono, S., Mizugaki, H., . . . Tamura, T. (2015). Epidermal growth factor receptor mutation is associated with longer local control after definitive chemoradiotherapy in patients with stage III nonsquamous non-small-cell lung cancer. Int J Radiat Oncol Biol Phys, 91(1), 140-148. doi:10.1016/j.ijrobp.2014.08.344
Yamaguchi, F., Kato, E., Wakabayashi, A., & Shikama, Y. (2019). Effect of osimertinib treatment on lung adenocarcinoma with squamous cell transformation harboring the T790M mutation: A case report and literature review. Mol Clin Oncol, 11(2), 127-131. doi:10.3892/mco.2019.1880
Yamaguchi, T., Isogai, S., Okamura, T., Uozu, S., Mieno, Y., Hoshino, T., . . . Imaizumi, K. (2015). Pharmacokinetics of gefitinib in a patient with non-small cell lung cancer undergoing continuous ambulatory peritoneal dialysis. Case Rep Oncol, 8(1), 78-82. doi:10.1159/000375485
Yamamoto, H., Toyooka, S., Ninomiya, T., Matsumoto, S., Kanai, M., Tomida, S., . . . Ladanyi, M. (2018). Therapeutic Potential of Afatinib for Cancers with ERBB2 (HER2) Transmembrane Domain Mutations G660D and V659E. Oncologist, 23(2), 150-154. doi:10.1634/theoncologist.2017-0345
Yamazaki, S., Higuchi, Y., Ishibashi, M., Hashimoto, H., Yasunaga, M., Matsumura, Y., . . . Ishii, G. (2018). Collagen type I induces EGFR-TKI resistance in EGFR-mutated cancer cells by mTOR activation through Akt-independent pathway. Cancer Sci, 109(6), 2063-2073. doi:10.1111/cas.13624
Yan, L., Zhang, J., Guo, D., Ma, J., Shui, S. F., & Han, X. W. (2019). IL-21R functions as an oncogenic factor and is regulated by the lncRNA MALAT1/miR-125a-3p axis in gastric cancer. Int J Oncol, 54(1), 7-16. doi:10.3892/ijo.2018.4612
Yanai, Y., Hayashi, T., Akazawa, Y., Yatagai, N., Tsuyama, S., Yao, T., & Saito, T. (2020). Clinicopathological and mutational differences between tumors with multiple metastases and single lung metastasis in colorectal cancer. Oncol Lett, 20(1), 541-550. doi:10.3892/ol.2020.11627
Yang, B., Luo, L., Luo, W., Zhou, Y., Yang, C., Xiong, T., . . . Wang, Z. (2017). The genomic dynamics during progression of lung adenocarcinomas. J Hum Genet, 62(8), 783-788. doi:10.1038/jhg.2017.40
Yang, L., Pang, C., Xu, F., Yang, G., Xu, H., Wang, C., & Wang, Y. (2020). Tumor Differentiation and EGFR Mutation Associated with Disease-Free Survival in Stage IA Lung Adenocarcinoma Patients with Curative Surgery. Cancer Manag Res, 12, 12549-12556. doi:10.2147/cmar.S286503
Yang, S., Song, Z., & Cheng, G. (2019). Genomic alterations and survival in young patients aged under 40 years with completely resected non-small cell lung cancer. Ann Transl Med, 7(7), 140. doi:10.21037/atm.2019.03.39
Yang, W., You, N., Jia, M., Yeung, S. J., Ou, W., Yu, M., . . . Cheng, C. (2020). Undetectable circulating tumor DNA levels correlate with low risk of recurrence/metastasis in postoperative pathologic stage I lung adenocarcinoma patients. Lung Cancer, 146, 327-334. doi:10.1016/j.lungcan.2020.06.009
Yang, X. N., Yan, H. H., Wang, J., Chu, X. Y., Liu, Z. D., Shen, Y., . . . Wu, Y. L. (2022). Real-World Survival Outcomes Based on EGFR Mutation Status in Chinese Patients With Lung Adenocarcinoma After Complete Resection: Results From the ICAN Study. JTO Clin Res Rep, 3(1), 100257. doi:10.1016/j.jtocrr.2021.100257
Yang, Y., Pu, Y., Dai, N., Wang, D., & Xu, M. (2020). Complete response of radioresistant brain metastases from non-small cell lung cancer with temozolomide: A case report and literature review. Medicine (Baltimore), 99(51), e23592. doi:10.1097/md.0000000000023592
Yang, Y., Xie, X., Jiang, G., Zhang, L., & Liu, H. (2022). Clinicopathological characteristic of ciliated muconodular papillary tumour of the lung. J Clin Pathol, 75(2), 128-132. doi:10.1136/jclinpath-2020-207205
Yang, Z. M., Ding, X. P., Pen, L., Mei, L., & Liu, T. (2014). Analysis of CEA expression and EGFR mutation status in non-small cell lung cancers. Asian Pac J Cancer Prev, 15(8), 3451-3455. doi:10.7314/apjcp.2014.15.8.3451
Yao, Y., Zhang, T., Qi, L., Liu, R., Liu, G., Li, J., & Sun, C. (2021). Identification of Four Genes as Prognosis Signatures in Lung Adenocarcinoma Microenvironment. Pharmgenomics Pers Med, 14, 15-26. doi:10.2147/pgpm.S283414
Yasukawa, M., Kawaguchi, T., Ota, M., Tojo, T., & Taniguchi, S. (2021). [Effectiveness of Osimertinib for Postoperative Recurrence of Lung Cancer with L861Q Activating EGFR Mutation:Report of a Case]. Kyobu Geka, 74(2), 156-159. 
Yi, L., Huang, P., Gu, Y., Wu, G., Zou, X., Guo, L., . . . Zhao, D. (2022). Clinical Significance and Immune Landscape of Recurrence-Associated Ferroptosis Signature in Early-Stage Lung Adenocarcinoma. Front Oncol, 12, 794293. doi:10.3389/fonc.2022.794293
Yin, J., Xi, J., Liang, J., Zhan, C., Jiang, W., Lin, Z., . . . Wang, Q. (2021). Solid Components in the Mediastinal Window of Computed Tomography Define a Distinct Subtype of Subsolid Nodules in Clinical Stage I Lung Cancers. Clin Lung Cancer, 22(4), 324-331. doi:10.1016/j.cllc.2021.02.015
Yokosuka, T., Kobayashi, T., Enomoto, T., & Takeda, A. (2013). [A case of recurrent non-small cell lung cancer successfully treated with multiple modality therapies including S-1 monotherapy as fifth-line chemotherapy hospital)]. Gan To Kagaku Ryoho, 40(9), 1201-1203. 
Yokoyama, Y., Sonobe, M., Yamada, T., Sato, M., Menju, T., Aoyama, A., . . . Date, H. (2015). Gefitinib treatment in patients with postoperative recurrent non-small-cell lung cancer harboring epidermal growth factor receptor gene mutations. Int J Clin Oncol, 20(6), 1122-1129. doi:10.1007/s10147-015-0838-z
Yomo, S., & Oda, K. (2018). Impacts of EGFR-mutation status and EGFR-TKI on the efficacy of stereotactic radiosurgery for brain metastases from non-small cell lung adenocarcinoma: A retrospective analysis of 133 consecutive patients. Lung Cancer, 119, 120-126. doi:10.1016/j.lungcan.2018.03.013
Yoshida, K., & Teramoto, S. (2015). [A case of cardiac tamponade due to malignant pericarditis with lung adenocarcinoma, effectively treated with pericardial drainage and pemetrexed plus cisplatin chemotherapy]. Nihon Ronen Igakkai Zasshi, 52(4), 421-424. doi:10.3143/geriatrics.52.421
Yoshida, T., Ishii, G., Goto, K., Neri, S., Hashimoto, H., Yoh, K., . . . Ochiai, A. (2015). Podoplanin-positive cancer-associated fibroblasts in the tumor microenvironment induce primary resistance to EGFR-TKIs in lung adenocarcinoma with EGFR mutation. Clin Cancer Res, 21(3), 642-651. doi:10.1158/1078-0432.Ccr-14-0846
Yoshida, T., Ishii, G., Goto, K., Yoh, K., Niho, S., Umemura, S., . . . Ochiai, A. (2013). Solid predominant histology predicts EGFR tyrosine kinase inhibitor response in patients with EGFR mutation-positive lung adenocarcinoma. J Cancer Res Clin Oncol, 139(10), 1691-1700. doi:10.1007/s00432-013-1495-0
Yoshida, T., Kuroda, H., Oya, Y., Shimizu, J., Horio, Y., Sakao, Y., . . . Yatabe, Y. (2017). Clinical outcomes of platinum-based chemotherapy according to T790M mutation status in EGFR-positive non-small cell lung cancer patients after initial EGFR-TKI failure. Lung Cancer, 109, 89-91. doi:10.1016/j.lungcan.2017.05.001
Yoshimura, K., Inoue, Y., Mori, K., Iwashita, Y., Kahyo, T., Kawase, A., . . . Sugimura, H. (2017). Distinct prognostic roles and heterogeneity of TTF1 copy number and TTF1 protein expression in non-small cell lung cancer. Genes Chromosomes Cancer, 56(7), 570-581. doi:10.1002/gcc.22461
Yotsukura, M., Asamura, H., Motoi, N., Kashima, J., Yoshida, Y., Nakagawa, K., . . . Watanabe, S. I. (2021). Long-Term Prognosis of Patients With Resected Adenocarcinoma In Situ and Minimally Invasive Adenocarcinoma of the Lung. J Thorac Oncol, 16(8), 1312-1320. doi:10.1016/j.jtho.2021.04.007
Yu, H. A., Perez, L., Chang, Q., Gao, S. P., Kris, M. G., Riely, G. J., & Bromberg, J. (2017). A Phase 1/2 Trial of Ruxolitinib and Erlotinib in Patients with EGFR-Mutant Lung Adenocarcinomas with Acquired Resistance to Erlotinib. J Thorac Oncol, 12(1), 102-109. doi:10.1016/j.jtho.2016.08.140
Yu, H. A., Planchard, D., & Lovly, C. M. (2018). Sequencing Therapy for Genetically Defined Subgroups of Non-Small Cell Lung Cancer. Am Soc Clin Oncol Educ Book, 38, 726-739. doi:10.1200/edbk_201331
Yu, H. A., Sima, C. S., Hellmann, M. D., Naidoo, J., Busby, N., Rodriguez, K., . . . Kris, M. G. (2015). Differences in the survival of patients with recurrent versus de novo metastatic KRAS-mutant and EGFR-mutant lung adenocarcinomas. Cancer, 121(12), 2078-2082. doi:10.1002/cncr.29313
Yu, H. A., Sima, C. S., Shen, R., Kass, S., Gainor, J., Shaw, A., . . . Riely, G. J. (2015). Prognostic impact of KRAS mutation subtypes in 677 patients with metastatic lung adenocarcinomas. J Thorac Oncol, 10(3), 431-437. doi:10.1097/jto.0000000000000432
Yu, R., He, Z., Lou, Y., Jiang, H., Wu, Y., Liu, Z., . . . Han, W. (2017). Clinical characteristics and programmed cell death ligand-1 expression in adenocarcinoma in situ and minimally invasive adenocarcinoma of lung. Oncotarget, 8(58), 97801-97810. doi:10.18632/oncotarget.22082
Yu, S., Zhang, Y., Pan, Y., Cheng, C., Sun, Y., & Chen, H. (2017). The non-small cell lung cancer EGFR extracellular domain mutation, M277E, is oncogenic and drug-sensitive. Onco Targets Ther, 10, 4507-4515. doi:10.2147/ott.S131999
Yuan, X., Yu, G., Hou, X., Shih Ie, M., Clarke, R., Zhang, J., . . . Wang, Y. (2012). Genome-wide identification of significant aberrations in cancer genome. BMC Genomics, 13, 342. doi:10.1186/1471-2164-13-342
Yuan, X., Zhang, J., Zhang, S., Yu, G., & Wang, Y. (2012). Comparative analysis of methods for identifying recurrent copy number alterations in cancer. PLoS One, 7(12), e52516. doi:10.1371/journal.pone.0052516
Zaric, B., Brcic, L., Buder, A., Brandstetter, A., Buresch, J. O., Traint, S., . . . Filipits, M. (2018). PD-1 and PD-L1 Protein Expression Predict Survival in Completely Resected Lung Adenocarcinoma. Clin Lung Cancer, 19(6), e957-e963. doi:10.1016/j.cllc.2018.08.014
Zenali, M. J., Weissferdt, A., Solis, L. M., Ali, S., Tang, X., Mehran, R. J., . . . Kalhor, N. (2015). An update on clinicopathological, immunohistochemical, and molecular profiles of colloid carcinoma of the lung. Hum Pathol, 46(6), 836-842. doi:10.1016/j.humpath.2014.10.032
Zhang, C., Wang, X., Zhang, M., Liu, D., & Yang, D. P. (2020). Neoadjuvant apatinib plus S-1 in locally advanced pulmonary adenocarcinoma: A case report and review of the literature. Medicine (Baltimore), 99(3), e18767. doi:10.1097/md.0000000000018767
Zhang, C., Yang, H., Lang, B., Yu, X., Xiao, P., Zhang, D., . . . Zhang, X. (2018). Surgical significance and efficacy of epidermal growth factor receptor tyrosine kinase inhibitors in patients with primary lung adenosquamous carcinoma. Cancer Manag Res, 10, 2401-2407. doi:10.2147/cmar.S165660
Zhang, C., Zhang, Z., Zhang, G., Zhang, Z., Luo, Y., Wang, F., . . . He, J. (2020). Clinical significance and inflammatory landscapes of a novel recurrence-associated immune signature in early-stage lung adenocarcinoma. Cancer Lett, 479, 31-41. doi:10.1016/j.canlet.2020.03.016
Zhang, J., Fujimoto, J., Zhang, J., Wedge, D. C., Song, X., Zhang, J., . . . Futreal, P. A. (2014). Intratumor heterogeneity in localized lung adenocarcinomas delineated by multiregion sequencing. Science, 346(6206), 256-259. doi:10.1126/science.1256930
Zhang, L., Li, S., Choi, Y. L., Lee, J., Gong, Z., Liu, X., . . . Chen, R. (2017). Systematic identification of cancer-related long noncoding RNAs and aberrant alternative splicing of quintuple-negative lung adenocarcinoma through RNA-Seq. Lung Cancer, 109, 21-27. doi:10.1016/j.lungcan.2017.04.009
Zhang, S., Yan, B., Zheng, J., Zhao, J., & Zhou, J. (2016). Gene status and clinicopathologic characteristics of lung adenocarcinomas with mediastinal lymph node metastasis. Oncotarget, 7(39), 63758-63766. doi:10.18632/oncotarget.11494
Zhang, X., Han, J., Du, L., Li, X., Hao, J., Wang, L., . . . Wang, C. (2020). Unique metastasis-associated lncRNA signature optimizes prediction of tumor relapse in lung adenocarcinoma. Thorac Cancer, 11(3), 728-737. doi:10.1111/1759-7714.13325
Zhang, X., Jiang, Y., Yu, H., Xia, H., & Wang, X. (2020). A comprehensive study on the oncogenic mutation and molecular pathology in Chinese lung adenocarcinoma patients. World J Surg Oncol, 18(1), 172. doi:10.1186/s12957-020-01947-z
Zhang, Y., Li, J., Wang, R., Li, Y., Pan, Y., Cai, D., . . . Chen, H. (2014). The prognostic and predictive value of solid subtype in invasive lung adenocarcinoma. Sci Rep, 4, 7163. doi:10.1038/srep07163
Zhang, Y., Ma, Y., Li, Y., Shen, X., Yu, Y., Pan, Y., . . . Chen, H. (2018). Are exon 19 deletions and L858R different in early stage lung adenocarcinoma? J Cancer Res Clin Oncol, 144(1), 165-171. doi:10.1007/s00432-017-2526-z
Zhang, Y., Ma, Y., Li, Y., Shen, X., Yu, Y., Pan, Y., . . . Chen, H. (2019). Comparative analysis of co-occurring mutations of specific tumor suppressor genes in lung adenocarcinoma between Asian and Caucasian populations. J Cancer Res Clin Oncol, 145(3), 747-757. doi:10.1007/s00432-018-02828-5
Zhang, Y., Sun, Y., Li, Y., Fang, Z., Wang, R., Pan, Y., . . . Ji, H. (2013). ANCCA protein expression is a novel independent poor prognostic marker in surgically resected lung adenocarcinoma. Ann Surg Oncol, 20 Suppl 3, S577-582. doi:10.1245/s10434-013-3027-1
Zhang, Y., Wang, R., Cai, D., Li, Y., Pan, Y., Hu, H., . . . Chen, H. (2014). A comprehensive investigation of molecular features and prognosis of lung adenocarcinoma with micropapillary component. J Thorac Oncol, 9(12), 1772-1778. doi:10.1097/jto.0000000000000341
Zhang, Y., Wang, R., Li, Y., Pan, Y., Hu, H., Zhang, Y., . . . Chen, H. (2015). Negative Thyroid Transcription Factor 1 Expression Defines an Unfavorable Subgroup of Lung Adenocarcinomas. J Thorac Oncol, 10(10), 1444-1450. doi:10.1097/jto.0000000000000626
Zhao, D., Chen, X., Qin, N., Su, D., Zhou, L., Zhang, Q., . . . Wang, J. (2017). The prognostic role of EGFR-TKIs for patients with advanced non-small cell lung cancer. Sci Rep, 7, 40374. doi:10.1038/srep40374
Zhao, M., Zhang, Y., Zhang, H., Wang, S., Zhang, M., Chen, X., . . . Zhou, C. (2015). Hypoxia-induced cell stemness leads to drug resistance and poor prognosis in lung adenocarcinoma. Lung Cancer, 87(2), 98-106. doi:10.1016/j.lungcan.2014.11.017
Zhao, P., Zhen, H., Zhao, H., Zhao, L., & Cao, B. (2022). Efficacy and safety of adjuvant EGFR-TKIs for resected non-small cell lung cancer: a systematic review and meta-analysis based on randomized control trials. BMC Cancer, 22(1), 328. doi:10.1186/s12885-022-09444-0
Zhao, Q. Y., Liu, L. P., Lu, L., Gui, R., & Luo, Y. W. (2021). A Novel Intercellular Communication-Associated Gene Signature for Prognostic Prediction and Clinical Value in Patients With Lung Adenocarcinoma. Front Genet, 12, 702424. doi:10.3389/fgene.2021.702424
Zhao, S., Cong, X., & Liu, Z. (2021). Successful treatment of 2 patients with brain metastases from non-small cell lung cancer with epidermal growth factor receptor mutation receiving dacomitinib: A case report. Medicine (Baltimore), 100(30), e26680. doi:10.1097/md.0000000000026680
Zhao, X., Yue, D., Qian, J., Zhang, L., Song, J., Zhang, B., . . . Wang, C. (2022). Case Report: Sarcoid-Like Reactions and Tertiary Lymphoid Structures Following Dual Checkpoint Inhibition in a Patient with Early-Stage Lung Adenocarcinoma. Front Immunol, 13, 794217. doi:10.3389/fimmu.2022.794217
Zhao, Y., Han, H., Gao, Z., Hu, H., Xiang, J., Sun, Y., & Chen, H. (2021). Evolutionary Action Score of TP53 Enhances the Prognostic Prediction for Stage I Lung Adenocarcinoma. Semin Thorac Cardiovasc Surg, 33(1), 221-229. doi:10.1053/j.semtcvs.2020.04.005
Zhao, Y., Pan, Y., Cheng, C., Zheng, D., Zhang, Y., Gao, Z., . . . Chen, H. (2020). EGFR-mutant lung adenocarcinoma harboring co-mutational tumor suppressor genes predicts poor prognosis. J Cancer Res Clin Oncol, 146(7), 1781-1789. doi:10.1007/s00432-020-03237-3
Zhao, Y., Varn, F. S., Cai, G., Xiao, F., Amos, C. I., & Cheng, C. (2018). A P53-Deficiency Gene Signature Predicts Recurrence Risk of Patients with Early-Stage Lung Adenocarcinoma. Cancer Epidemiol Biomarkers Prev, 27(1), 86-95. doi:10.1158/1055-9965.Epi-17-0478
Zhao, Y., Yang, J., Chen, Z., Gao, Z., Zhou, F., Li, X., . . . He, J. (2014). Identification of somatic alterations in stage I lung adenocarcinomas by next-generation sequencing. Genes Chromosomes Cancer, 53(4), 289-298. doi:10.1002/gcc.22138
Zheng, D., Wang, R., Pan, Y., Zheng, S., Zhang, Y., Li, H., . . . Chen, H. (2015). Prevalence and Clinicopathological Characteristics of BRAF Mutations in Chinese Patients with Lung Adenocarcinoma. Ann Surg Oncol, 22 Suppl 3, S1284-1291. doi:10.1245/s10434-015-4640-y
Zheng, D., Wang, R., Zhang, Y., Pan, Y., Cheng, X., Cheng, C., . . . Chen, H. (2016). Prevalence and clinicopathological characteristics of ALK fusion subtypes in lung adenocarcinomas from Chinese populations. J Cancer Res Clin Oncol, 142(4), 833-843. doi:10.1007/s00432-015-2081-4
Zheng, Y., Fang, W., Liu, X., & Xu, N. (2012). New EGFR-TKI: a case report of recurrent lung adenocarcinoma successfully treated with icotinib. Tumori, 98(4), e102-104. doi:10.1700/1146.12653
Zhong, Y., Jiang, L., Lin, H., Li, X., Long, X., Zhou, Y., . . . Li, Z. (2019). Overexpression of KIF18A promotes cell proliferation, inhibits apoptosis, and independently predicts unfavorable prognosis in lung adenocarcinoma. IUBMB Life, 71(7), 942-955. doi:10.1002/iub.2030
Zhong, Y., Jiang, L., Long, X., Zhou, Y., Deng, S., Lin, H., & Li, X. (2019). Clinical Significance And Integrative Analysis Of Kinesin Family Member 18B In Lung Adenocarcinoma. Onco Targets Ther, 12, 9249-9264. doi:10.2147/ott.S227438
Zhou, S., Wang, P., Su, X., Chen, J., Chen, H., Yang, H., . . . Yang, J. (2017). High ECT2 expression is an independent prognostic factor for poor overall survival and recurrence-free survival in non-small cell lung adenocarcinoma. PLoS One, 12(10), e0187356. doi:10.1371/journal.pone.0187356
Zhou, W., Liu, Z., Wang, Y., Zhang, Y., Qian, F., Lu, J., . . . Zhang, W. (2022). The clinicopathological and molecular characteristics of resected EGFR-mutant lung adenocarcinoma. Cancer Med, 11(5), 1299-1309. doi:10.1002/cam4.4543
Zhou, X., Cai, L., Liu, J., Hua, X., Zhang, Y., Zhao, H., . . . Gai, P. (2018). Analyzing EGFR mutations and their association with clinicopathological characteristics and prognosis of patients with lung adenocarcinoma. Oncol Lett, 16(1), 362-370. doi:10.3892/ol.2018.8681
Zhou, X., Liu, G., Lin, M., Li, L., & Cao, D. (2022). Identification of LHFPL3-AS2 as a prognostic biomarker in lung adenocarcinoma. Indian J Pathol Microbiol, 65(2), 288-295. doi:10.4103/ijpm.Ijpm_1404_20
Zhu, H., Wong, M. P., & Tin, V. (2014). High-resolution detection of recurrent aberrations in lung adenocarcinomas by array comparative genomic hybridization and expression analysis of selective genes by quantitative PCR. Int J Oncol, 44(6), 2068-2076. doi:10.3892/ijo.2014.2384
Zhu, J., Lu, Q., Li, B., Li, H., Wu, C., Li, C., & Jin, H. (2021). Potential of the cell-free blood-based biomarker uroplakin 2 RNA to detect recurrence after surgical resection of lung adenocarcinoma. Oncol Lett, 22(1), 520. doi:10.3892/ol.2021.12781
Zhu, M., Ren, Y., Liu, Y., Ban, C., Gu, H., Wang, Z., & Zhang, Y. (2016). [Analysis of Clinicopathological Feature and Prognosis for  Leptomeningeal Metastasis in Non-small Cell Lung Cancer]. Zhongguo Fei Ai Za Zhi, 19(8), 533-538. doi:10.3779/j.issn.1009-3419.2016.08.09
Zhu, M., Zeng, Q., Fan, T., Lei, Y., Wang, F., Zheng, S., . . . He, J. (2022). Clinical Significance and Immunometabolism Landscapes of a Novel Recurrence-Associated Lipid Metabolism Signature In Early-Stage Lung Adenocarcinoma: A Comprehensive Analysis. Front Immunol, 13, 783495. doi:10.3389/fimmu.2022.783495
Zhu, S., Zhao, S., Zhang, Q., Li, S., Ren, D., Ren, F., . . . Xu, S. (2021). Complete disease remission in a TP53 and KRAS co-mutated brain oligometastatic lung cancer patient after immuno-chemotherapy and surgical resection: a case report. Transl Lung Cancer Res, 10(5), 2298-2305. doi:10.21037/tlcr-21-380
Zhu, X., Hou, R., Li, X., Jiang, C., Xia, W., & Fu, X. (2020). Predictive model of the first failure pattern in patients receiving definitive chemoradiotherapy for inoperable locally advanced non-small cell lung cancer (LA-NSCLC). Radiat Oncol, 15(1), 43. doi:10.1186/s13014-020-1467-x
Zhu, Y. C., Liao, X. H., Wang, W. X., Xu, C. W., Zhuang, W., Wei, J. G., & Du, K. Q. (2018). Dual drive coexistence of EML4-ALK and TPM3-ROS1 fusion in advanced lung adenocarcinoma. Thorac Cancer, 9(2), 324-327. doi:10.1111/1759-7714.12578
Ziv, E., Erinjeri, J. P., Yarmohammadi, H., Boas, F. E., Petre, E. N., Gao, S., . . . Solomon, S. B. (2017). Lung Adenocarcinoma: Predictive Value of KRAS Mutation Status in Assessing Local Recurrence in Patients Undergoing Image-guided Ablation. Radiology, 282(1), 251-258. doi:10.1148/radiol.2016160003
