# Supplementary material for: Prognosis of recurrence after complete resection in early-stage lung adenocarcinoma based on molecular alterations: a systematic review and meta-analysis
Source: Sci Rep. 2023 Oct 31;13:18710. doi: 10.1038/s41598-023-42851-2 (PMC10618289; doi:10.1038/s41598-023-42851-2)
Supplement: Supplementary file 1 — Supplementary Information 1. [file 41598_2023_42851_MOESM1_ESM.zip › Additional Files/Web of Science-lung adenocarcinoma, recurrence, genomic characteristics, and mutation.rtf]

Ahmad, Zeeshan, Raza, Ahmad, & Patel, Manish R. (2015). Endometrial metastasis of lung adenocarcinoma: a report of two cases. The American journal of case reports, 16, 296-299. doi:10.12659/ajcr.892495
Aoki, Masaya, Ueda, Kazuhiro, Umehara, Tadashi, Kamimura, Go, Tokunaga, Takuya, Harada-Takeda, Aya, . . . Sato, Masami. (2020). Targeted Therapy Followed by Cytotoxic Chemotherapy in Preoperative Patients With Locally Advanced Lung Adenocarcinoma. Anticancer Research, 40(5), 2911-2916. doi:10.21873/anticanres.14268
Bai, Jinsong, Deng, Chaoqiang, Zheng, Qiang, Li, Di, Fu, Fangqiu, Li, Yuan, . . . Chen, Haiquan. (2022). Comprehensive analysis of mutational profile and prognostic significance of complex glandular pattern in lung adenocarcinoma. Translational Lung Cancer Research, 11(7), 1337-+. doi:10.21037/tlcr-22-127
Behrens, Carmen, Solis, Luisa M., Lin, Heather, Yuan, Ping, Tang, Ximing, Kadara, Humam, . . . Wistuba, Ignacio I. (2013). EZH2 Protein Expression Associates with the Early Pathogenesis, Tumor Progression, and Prognosis of Non-Small Cell Lung Carcinoma. Clinical Cancer Research, 19(23), 6556-6565. doi:10.1158/1078-0432.Ccr-12-3946
Brunner, N. A., Christensen, I. J., Moreira, J., Pfeiffer, P., & Tarpgaard, L. S. WO2015144184-A1; WO2015144184-A8.
Bulutay, Pinar, AkyUrek, Nalan, & MemiS, Leyla. (2021). Clinicopathological and Prognostic Significance of the EML4-ALK Translocation and IGFR1, TTF1, Napsin A Expression in Patients with Lung Adenocarcinoma. [Clinicopathological and Prognostic Significance of the EML4-ALK Translocation and IGFR1, TTF1, Napsin A Expression in Patients with Lung Adenocarcinoma.]. Turk patoloji dergisi, 37(1), 7-17. doi:10.5146/tjpath.2020.01503
Cai, Deng, Li, Hang, Wang, Rui, Li, Yuan, Pan, Yunjian, Hu, Haichuan, . . . Chen, Haiquan. (2014). Comparison of clinical features, molecular alterations, and prognosis in morphological subgroups of lung invasive mucinous adenocarcinoma. Oncotargets and Therapy, 7, 2127-2132. doi:10.2147/ott.S70984
Caso, Raul, Sanchez-Vega, Francisco, Tan, Kay See, Mastrogiacomo, Brooke, Zhou, Jian, Jones, Gregory D., . . . Jones, David R. (2020). The Underlying Tumor Genomics of Predominant Histologic Subtypes in Lung Adenocarcinoma. Journal of Thoracic Oncology, 15(12), 1844-1856. doi:10.1016/j.jtho.2020.08.005
Chang, Jason C., Offin, Michael, Falcon, Christina, Brown, David, Houck-Loomis, Brian R., Meng, Fanli, . . . Rekhtman, Natasha. (2021). Comprehensive Molecular and Clinicopathologic Analysis of 200 Pulmonary Invasive Mucinous Adenocarcinomas Identifies Distinct Characteristics of Molecular Subtypes. Clinical Cancer Research, 27(14), 4066-4076. doi:10.1158/1078-0432.Ccr-21-0423
Chen, Shiqi, Yang, Siqian, Zhang, Yang, Xiang, Jiaqing, Zhang, Yawei, Hu, Hong, . . . Ye, Ting. (2021). Clinicopathologic features and prognostic value of epidermal growth factor receptor mutation in patients with pT1a and pT1b invasive lung adenocarcinoma after surgical resection. Journal of Thoracic Disease, 13(9), 5496-+. doi:10.21037/jtd-21-924
Cheng, Chao, Wang, Rui, Li, Yuan, Pan, Yunjian, Zhang, Yang, Li, Hang, . . . Chen, Haiquan. (2015). EGFR Exon 18 Mutations in East Asian Patients with Lung Adenocarcinomas: A Comprehensive Investigation of Prevalence, Clinicopathologic Characteristics and Prognosis. Scientific Reports, 5. doi:10.1038/srep13959
Choi, Helen, Kratz, Johannes, Pham, Patrick, Lee, Sharon, Ray, Roshni, Kwon, Yong-Won, . . . Kim, Il-Jin. (2012). Development of a rapid and practical mutation screening assay for human lung adenocarcinoma. International Journal of Oncology, 40(6), 1900-1906. doi:10.3892/ijo.2012.1396
Chow, Oliver S., Villena-Vargas, Jonathan, Nasar, Abu, Sun, Brian, Harrison, Sebron, Lee, Benjamin, . . . Stiles, Brendon M. (2022). Outcomes After Surgical Resection of Early-stage Lung Adenocarcinomas With Epidermal Growth Factor Receptor Mutations. Annals of Thoracic Surgery, 114(3), 905-910. doi:10.1016/j.athoracsur.2021.07.077
D'Angelo, Sandra P., Janjigian, Yelena Y., Ahye, Nicholas, Riely, Gregory J., Chaft, Jamie E., Sima, Camelia S., . . . Azzoli, Christopher G. (2012). Distinct Clinical Course of EGFR-Mutant Resected Lung Cancers Results of Testing of 1118 Surgical Specimens and Effects of Adjuvant Gefitinib and Erlotinib. Journal of Thoracic Oncology, 7(12), 1815-1822. doi:10.1097/JTO.0b013e31826bb7b2
Deng, Chaoqiang, Zhang, Yang, Fu, Fangqiu, Ma, Xiangyi, Wen, Zhexu, Ma, Zelin, . . . Chen, Haiquan. (2021). Genetic-pathological prediction for timing and site-specific recurrence pattern in resected lung adenocarcinoma. European Journal of Cardio-Thoracic Surgery, 60(5), 1223-1231. doi:10.1093/ejcts/ezab288
Devarashetty, Sindhu, Chennapragada, Suma Sri, & Mansour, Richard. (2022). Not Your Typical Adenocarcinoma: A Case of Mesonephric Adenocarcinoma of the Cervix With Fibroblast Growth Factor Receptor 2 (FGFR2) Mutation. Cureus, 14(5), e25098-e25098. doi:10.7759/cureus.25098
Dong, Yuanmei, Liu, Yang, Bai, Hui, & Jiao, Shunchang. (2019). Systematic assessment of the clinicopathological prognostic significance of tissue cytokine expression for lung adenocarcinoma based on integrative analysis of TCGA data. Scientific Reports, 9. doi:10.1038/s41598-019-42345-0
Eide, Inger Johanne Zwicky, Nilssen, Yngvar, Lund-Iversen, Marius, & Brustugun, Odd Terje. (2022). Factors affecting outcome in resected EGFR-mutated lung cancer. Acta Oncologica, 61(6), 749-756. doi:10.1080/0284186x.2022.2066984
Fan, Lei, & He, Ping. (2022). Research Progress on Spread Through Air Spaces of Lung Cancer. Zhongguo fei ai za zhi = Chinese journal of lung cancer, 25(1), 54-60. doi:10.3779/j.issn.1009-3419.2021.101.49
Gao, Mao-Gang, Wang, Shi-Ze, Han, Kai-Hong, Xie, Shao-Nan, & Liu, Qing-Yi. (2022). Clinical characteristics and prognostic value of EGFR mutation in stage I lung adenocarcinoma with spread through air spaces after surgical resection. Neoplasma, 69(6), 1480-1489. doi:10.4149/neo_2022_220619N649
Gao, Xuejuan, Zhao, Yanfeng, Bao, Yi, Yin, Wei, Liu, Liyu, Liu, Ruchuan, . . . Shuai, Jianwei. (2019). Poor Prognosis With Coexistence Of EGFR T790M Mutation And Common EGFR-Activating Mutation In Non- Small Cell Lung Cancer. Cancer Management and Research, 11, 9621-9630. doi:10.2147/cmar.S216721
Ge, Jianjun, Yao, Bin, Huang, Jia, Wu, Xue, Bao, Hua, Ou, Qiuxiang, . . . Chen, Jun. (2019). Molecular genetic characterization reveals linear tumor evolution in a pulmonary sarcomatoid carcinomas patient with a novel PHF20-NTRK1 fusion: a case report. Bmc Cancer, 19. doi:10.1186/s12885-019-5780-4
Guidry, Kayla, Vasudevaraja, Varshini, Labbe, Kristen, Mohamed, Hussein, Serrano, Jonathan, Guidry, Brett W., . . . Wong, Kwok-Kin. (2022). DNA Methylation Profiling Identifies Subgroups of Lung Adenocarcinoma with Distinct Immune Cell Composition, DNA Methylation Age, and Clinical Outcome. Clinical Cancer Research, 28(17), 3824-3835. doi:10.1158/1078-0432.Ccr-22-0391
Hamada, Kenichi, Tian, Ying, Fujimoto, Mao, Takahashi, Yoriko, Kohno, Takashi, Tsuta, Koji, . . . Arai, Eri. (2021). DNA hypermethylation of the ZNF132 gene participates in the clinicopathological aggressiveness of 'pan-negative'-type lung adenocarcinomas. Carcinogenesis, 42(2), 169-179. doi:10.1093/carcin/bgaa115
Hayasaka, Kazuki, Shiono, Satoshi, Matsumura, Yuki, Yanagawa, Naoki, Suzuki, Hiroyuki, Abe, Jiro, . . . Okada, Yoshinori. (2018). Epidermal Growth Factor Receptor Mutation as a Risk Factor for Recurrence in Lung Adenocarcinoma. Annals of Thoracic Surgery, 105(6), 1648-1654. doi:10.1016/j.athoracsur.2018.01.052
Higuchi, Mitsunori, Owada, Yuki, Inoue, Takuya, Watanabe, Yuzuru, Yamaura, Takumi, Fukuhara, Mitsuro, . . . Suzuki, Hiroyuki. (2016). FDG-PET in the evaluation of response to nivolumab in recurrent non-small-cell lung cancer. World Journal of Surgical Oncology, 14. doi:10.1186/s12957-016-0998-y
Horn, Lars-Christian, Hoehn, Anne Kathrin, Kruecken, Irene, Stiller, Mathias, Obeck, Ulrike, & Brambs, Christine E. (2020). Mesonephric-like adenocarcinomas of the uterine corpus: report of a case series and review of the literature indicating poor prognosis for this subtype of endometrial adenocarcinoma. Journal of Cancer Research and Clinical Oncology, 146(4), 971-983. doi:10.1007/s00432-019-03123-7
Hsu, Yi-Chiung, Chang, Ya-Hsuan, Chang, Gee-Chen, Ho, Bing-Ching, Yuan, Shin-Sheng, Li, Yu-Cheng, . . . Chen, Hsuan-Yu. (2019). Tumor mutation burden and recurrent tumors in hereditary lung cancer. Cancer Medicine, 8(5), 2179-2187. doi:10.1002/cam4.2120
Huang, Yen-Lin, Chen, Yi-Jung, Juan, Yi-Hsiu, Wu, Shang-Gin, & Chung, Kuei-Pin. (2022). Prognostic significance of dynamin-related protein 1 expression in advanced lung adenocarcinoma. Pathology Research and Practice, 234. doi:10.1016/j.prp.2022.153931
Igarashi, Yasuaki, Hamabashiri, Masato, Nishi, Mariko, Sugi, Yuri, Kakimoto, Hideki, Uchiyama, Masanobu, . . . Kamimura, Hidetoshi. (2021). A Case of Rheumatoid Arthritis Caused by Pembrolizumab Treatment for Non-Small Cell Lung Cancer. Gan to kagaku ryoho. Cancer & chemotherapy, 48(6), 837-839. 
Isaka, Tetsuya, Ito, Hiroyuki, Yokose, Tomoyuki, Saito, Haruhiro, Adachi, Hiroyuki, Murakami, Kotaro, . . . Rino, Yasushi. (2022). Prognostic factors for relapse-free survival in stage IB-IIIA primary lung adenocarcinoma by epidermal growth factor receptor mutation status. Bmc Cancer, 22(1). doi:10.1186/s12885-022-10057-w
Isaka, Tetsuya, Nakayama, Haruhiko, Ito, Hiroyuki, Yokose, Tomoyuki, Yamada, Kouzo, & Masuda, Munetaka. (2018). Impact of the epidermal growth factor receptor mutation status on the prognosis of recurrent adenocarcinoma of the lung after curative surgery. Bmc Cancer, 18. doi:10.1186/s12885-018-4849-9
Isaksson, Sofi, George, Anthony M., Jonsson, Mats, Cirenajwis, Helena, Jonsson, Per, Bendahl, Par-Ola, . . . Planck, Maria. (2019). Pre-operative plasma cell-free circulating tumor DNA and serum protein tumor markers as predictors of lung adenocarcinoma recurrence. Acta Oncologica, 58(8), 1079-1086. doi:10.1080/0284186x.2019.1610573
Ito, Masaoki, Miyata, Yoshihiro, Hirano, Shoko, Kimura, Shingo, Irisuna, Fumiko, Ikeda, Kyoko, . . . Okada, Morihito. (2019). Synchronicity of genetic variants between primary sites and metastatic lymph nodes, and prognostic impact in nodal metastatic lung adenocarcinoma. Journal of Cancer Research and Clinical Oncology, 145(9), 2325-2333. doi:10.1007/s00432-019-02978-0
Ito, Masaoki, Miyata, Yoshihiro, Kushitani, Kei, Yoshiya, Tomoharu, Kai, Yuichiro, Tsutani, Yasuhiro, . . . Okada, Morihito. (2018). Increased risk of recurrence in resected EGFR-positive pN0M0 invasive lung adenocarcinoma. Thoracic Cancer, 9(12), 1594-1602. doi:10.1111/1759-7714.12866
Ito, Masaoki, Miyata, Yoshihiro, Tsutani, Yasuhiro, Ito, Hiroyuki, Nakayama, Haruhiko, Imai, Kentaro, . . . Okada, Morihito. (2020). Positive EGFR mutation status is a risk of recurrence in pN0-1 lung adenocarcinoma when combined with pathological stage and histological subtype: A retrospective multi-center analysis. Lung Cancer, 141, 107-113. doi:10.1016/j.lungcan.2020.01.018
Izar, Benjamin, Zhou, Haiyu, Heist, Rebecca S., Azzoli, Christopher G., Muzikansky, Alona, Scribner, Emily E. F., . . . Lanuti, Michael. (2014). The Prognostic Impact of KRAS, Its Codon and Amino Acid Specific Mutations, on Survival in Resected Stage I Lung Adenocarcinoma. Journal of Thoracic Oncology, 9(9), 1363-1369. doi:10.1097/jto.0000000000000266
Jing, Wei, Ma, Jie-Tao, & Han, Cheng-Bo. (2020). Metastatic Breast Cancer Coexisting With HER-2 Amplification and EGFR Exon 19 Deletion Benefits From EGFR-TKI Therapy: A Case Report. Frontiers in Oncology, 10. doi:10.3389/fonc.2020.00771
Kadara, H., Choi, M., Zhang, J., Parra, E. R., Rodriguez-Canales, J., Gaffney, S. G., . . . Herbst, R. S. (2017). Whole-exome sequencing and immune profiling of early-stage lung adenocarcinoma with fully annotated clinical follow-up. Annals of oncology : official journal of the European Society for Medical Oncology, 28(1), 75-82. doi:10.1093/annonc/mdw436
Kadota, Kyuichi, Sima, Camelia S., Arcila, Maria E., Hedvat, Cyrus, Kris, Mark G., Jones, David R., . . . Travis, William D. (2016). KRAS Mutation Is a Significant Prognostic Factor in Early-stage Lung Adenocarcinoma. American Journal of Surgical Pathology, 40(12), 1579-1590. doi:10.1097/pas.0000000000000744
Kamiyoshihara, Mitsuhiro, Igai, Hitoshi, Ibe, Takashi, Ohsawa, Fumi, Yoshikawa, Ryohei, Shimizu, Kimihiro, . . . Kuwano, Hiroyuki. (2018). Multidisciplinary Approach to Recurrence after Resection of Primary Lung Cancer. Kyobu geka. The Japanese journal of thoracic surgery, 71(4), 302-310. 
Kang, Jin-Hyoung, Kim, Tae Jung, Kim, Seungjoon, 김민아, 김인호, 성숙환, & 허솔미. (2016). Coexistence of an EGFR Mutation and an ALK Rearrangement in a Patient with Lung Adenocarcinoma: a Case Report. The Korean Journal of Medicine, 90(1), 72-77. doi:10.3904/kjm.2016.90.1.72
Kaseda, Kaoru, Asakura, Keisuke, Kazama, Akio, & Ozawa, Yukihiko. (2017). Clinicopathological and prognostic features of surgically resected pathological stage I lung adenocarcinoma harboring epidermal growth factor receptor and K-ras mutation. Thoracic Cancer, 8(3), 229-237. doi:10.1111/1759-7714.12428
Kim, In Ae, Hur, Jae Young, Kim, Hee Joung, Lee, Song Am, Hwang, Jae Joon, Kim, Wan Seop, & Lee, Kye Young. (2021). Targeted Next-Generation Sequencing Analysis Predicts the Recurrence in Resected Lung Adenocarcinoma Harboring EGFR Mutations. Cancers, 13(14). doi:10.3390/cancers13143632
Kim, Ji Eun, Kim, Hyojin, Choe, Ji-Young, Sun, Pingli, Jheon, Sanghoon, & Chung, Jin-Haeng. (2013). High Expression of Sonic Hedgehog Signaling Proteins Is Related to the Favorable Outcome, EGFR Mutation, and Lepidic Predominant Subtype in Primary Lung Adenocarcinoma. Annals of Surgical Oncology, 20, S570-S576. doi:10.1245/s10434-013-3022-6
Kim, Min Hwan, Shim, Hyo Sup, Kang, Dae Ryong, Jung, Ji Ye, Lee, Chang Young, Kim, Dae Joon, . . . Cho, Byoung Chul. (2014). Clinical and prognostic implications of ALK and ROS1 rearrangements in never-smokers with surgically resected lung adenocarcinoma. Lung Cancer, 83(3), 389-395. doi:10.1016/j.lungcan.2014.01.003
Kim, Yong-il, Paeng, Jin Chul, Park, Young Sik, Cheon, Gi Jeong, Lee, Dong Soo, Chung, June-Key, & Kang, Keon Wook. (2018). Relation of EGFR Mutation Status to Metabolic Activity in Localized Lung Adenocarcinoma and Its Influence on the Use of FDG PET/CT Parameters in Prognosis. American Journal of Roentgenology, 210(6), 1346-1351. doi:10.2214/ajr.17.18916
Kitahara, Hirokazu, Okamoto, Tatsuro, Shimamatsu, Shinichiro, Kohno, Mikihiro, Morodomi, Yosuke, Tagawa, Tetsuzo, . . . Mori, Masaki. (2020). LINE-1 Hypomethylation Is Associated With Malignant Traits and Cell Proliferation in Lung Adenocarcinoma. Anticancer Research, 40(10), 5659-5666. doi:10.21873/anticanres.14579
Kobayashi, Haruki, Wakuda, Kazushige, & Takahashi, Toshiaki. (2016). Effectiveness of afatinib in lung cancer with paralytic ileus due to peritoneal carcinomatosis. Respirology case reports, 4(6), e00197-e00197. doi:10.1002/rcr2.197
Kratz, Johannes R., Li, Jack Z., Tsui, Jessica, Lee, Jen C., Ding, Vivianne W., Rao, Arjun A., . . . Jablons, David M. (2021). Genetic and immunologic features of recurrent stage I lung adenocarcinoma. Scientific Reports, 11(1). doi:10.1038/s41598-021-02946-0
Kudo, Yujin, Shimada, Yoshihisa, Saji, Hisashi, Kato, Yasufumi, Yoshida, Koichi, Matsubayashi, Jun, . . . Ikeda, Norihiko. (2015). Prognostic Factors for Survival After Recurrence in Patients With Completely Resected Lung Adenocarcinoma: Important Roles of Epidermal Growth Factor Receptor Mutation Status and the Current Staging System. Clinical Lung Cancer, 16(6), E213-E221. doi:10.1016/j.cllc.2015.04.005
Lee, Geun Dong, Lee, Seung Eun, Oh, Doo-Yi, Yu, Dan-Bi, Jeong, Hae Min, Kim, Jooseok, . . . Kim, Hyeong Ryul. (2017). MET Exon 14 Skipping Mutations in Lung Adenocarcinoma: Clinicopathologic Implications and Prognostic Values. Journal of Thoracic Oncology, 12(8), 1233-1246. doi:10.1016/j.jtho.2017.04.031
Lee, Kanghoon, Kim, Hyeong Ryul, Kim, Dong Kwan, Kim, Yong-Hee, Park, Seung-Ill, Choi, Se Hoon, & Han, Junhee. (2017). Post-recurrence survival analysis of stage I non-small-cell lung cancer. Asian cardiovascular & thoracic annals, 25(9), 623-629. doi:10.1177/0218492317737641
Li, Hefei, Dong, Shaoyong, Zhang, Duo, Guo, Zhimin, Li, Ce, Xiang, Jianxing, . . . Li, Wei. (2021). Targeted Sequencing Facilitated Diagnosis of an Uncommon Patient Harboring Both Multiple Primary and Intrapulmonary Metastatic Lung Cancer: A Case Report. Oncotargets and Therapy, 14, 3455-3459. doi:10.2147/ott.S309155
Li, Rui, Liu, Junfang, Fang, Zekui, Liang, Zhenyu, & Chen, Xin. (2020). Identification of Mutations Related to Cisplatin-Resistance and Prognosis of Patients With Lung Adenocarcinoma. Frontiers in Pharmacology, 11. doi:10.3389/fphar.2020.572627
Lin, Chun-Yu, Wu, Yen-Mu, Hsieh, Meng-Heng, Wang, Chih-Wei, Wu, Ching-Yang, Chen, Ying-Jen, & Fang, Yueh-Fu. (2017). Prognostic implication of EGFR gene mutations and histological classification in patients with resected stage I lung adenocarcinoma. Plos One, 12(10). doi:10.1371/journal.pone.0186567
Liu, Ming, Zhou, Chenzhi, & Zheng, Jian. (2015). Cigarette smoking impairs the response of EGFR-TKIs therapy in lung adenocarcinoma patients by promoting EGFR signaling and epithelial-mesenchymal transition. American Journal of Translational Research, 7(10), 2026-2035. 
Liu, Pingping, Wang, Cuiping, Wu, Shafei, Gao, Jie, & Zeng, Xuan. (2014). Clinicopathologic correlation and ALK rearrangement in adenocarcinoma of lung. Zhonghua bing li xue za zhi = Chinese journal of pathology, 43(4), 241-245. 
Liu, Shuli, Liu, Nan, Xiao, Mingming, Wang, Liang, & Wang, En-Hua. (2020). First case of bronchiolar adenoma lined purely by mucinous luminal cells with molecular analysis A case report. Medicine, 99(39). doi:10.1097/md.0000000000022322
Liu, Xianping, Li, Xiao, Zhang, Chao, Jin, Jian, Wang, Zhenfan, Xiao, Rongxin, . . . Yang, Fan. (2022). EGFR mutation is not a prognostic factor for CNS metastasis in curatively resected adenocarcinoma. Lung Cancer, 167, 78-86. doi:10.1016/j.lungcan.2022.03.013
Lui, Natalie S., Benson, Jalen, He, Hao, Imielski, Bartlomiej R., Kunder, Christian A., Liou, Douglas Z., . . . Shrager, Joseph B. (2020). Sub-solid lung adenocarcinoma in Asian versus Caucasian patients: different biology but similar outcomes. Journal of Thoracic Disease, 12(5), 2161-2171. doi:10.21037/jtd.2020.04.37
Lv, Tangfeng, Zou, Jiawei, Liu, Hongbing, Shen, Qin, Lu, Zhenfeng, Zhou, XiaoJun, . . . Song, Yong. (2017). Detection of oncogenic mutations in resected bronchial margins by next-generation sequencing indicates early relapse in stage IA lung adenocarcinoma patients. Oncotarget, 8(25), 40643-40653. doi:10.18632/oncotarget.16539
Lv, Yan-Ling, Liu, Hong-Bing, Yuan, Dong-Mei, Zhou, Li, Jin, Shu-Xian, & Song, Yong. (2019). Carcinoembryonic antigen in pleural effusion of patients with lung adenocarcinoma: a predictive marker for EGFR mutation. Translational Cancer Research, 8(4), 1027-1034. doi:10.21037/tcr.2019.06.10
Makimoto, Go, Ninomiya, Kiichiro, Kubo, Toshio, Sunami, Ryota, Kato, Yuka, Ichihara, Eiki, . . . Kiura, Katsuyuki. (2021). A novel osimertinib-resistant human lung adenocarcinoma cell line harbouring mutant EGFR and activated IGF1R. Japanese Journal of Clinical Oncology, 51(6), 956-965. doi:10.1093/jjco/hyab048
Masubuchi, Hiroaki, Maeno, Toshitaka, Uchida, Megumi, Kono, Shunichi, Suzuki, Masafumi, Takemura, Masao, . . . Kurabayashi, Masahiko. (2015). A case of Trousseau syndrome caused by pulmonary adenocarcinoma that was controlled for one year and 10 months with thrombosis treatment using an EGFR tyrosine kinase inhibitor and chemotherapy. Respiratory medicine case reports, 15, 101-105. doi:10.1016/j.rmcr.2015.05.001
Matsumura, Yuki, Owada, Yuki, Yamaura, Takumi, Muto, Satoshi, Osugi, Jun, Hoshino, Mika, . . . Gotoh, Mitsukazu. (2016). Epidermal growth factor receptor gene mutation as risk factor for recurrence in patients with surgically resected lung adenocarcinoma: a matched-pair analysis. Interactive Cardiovascular and Thoracic Surgery, 23(2), 216-222. doi:10.1093/icvts/ivw116
Matsumura, Yuki, Suzuki, Hiroyuki, Ohira, Tetsuya, Shiono, Satoshi, Abe, Jiro, Sagawa, Motoyasu, . . . Okada, Yoshinori. (2017). Matched-pair analysis of a multi-institutional cohort reveals that epidermal growth factor receptor mutation is not a risk factor for postoperative recurrence of lung adenocarcinoma. Lung Cancer, 114, 23-30. doi:10.1016/j.lungcan.2017.09.003
Matsuura, Yosuke, Ninomiya, Hironori, Ichinose, Junji, Nakao, Masayuki, Okumura, Sakae, Nishio, Makoto, & Mun, Mingyon. (2022). Prognostic impact and distinctive characteristics of surgically resected anaplastic lymphoma kinase-rearranged lung adenocarcinoma. Journal of Thoracic and Cardiovascular Surgery, 163(2), 441-+. doi:10.1016/j.jtcvs.2020.09.120
Mincer, J., Fischer, G., & McCormick, P. WO2022197770-A1.
Mirtavoos-Mahyari, Hanifeh, Abbasi Dezfouli, Azizollah, Esfahani-Monfared, Zahra, Khosravi, Adnan, Seifi, Sharareh, & Sheikhy, Kambiz. (2022). T790M and Acquired Resistance of Epidermal Growth Factor Receptor to Tyrosine Kinase Inhibitors in Patients with Lung Adenocarcinoma. Tanaffos, 21(3), 376-383. 
Miyata, Ryo, Hamaji, Masatsugu, Kawaguchi, Atsushi, Shimazu, Yumeta, Ikeda, Masaki, Ishikawa, Masashi, . . . Date, Hiroshi. (2022). Epidermal growth factor receptor tyrosine kinase inhibitors as first-line treatment for postoperative recurrent EGFR-mutated lung adenocarcinoma: a multi-institutional retrospective study. European Journal of Cardio-Thoracic Surgery, 62(5). doi:10.1093/ejcts/ezac430
Muto, Satoshi, Ozaki, Yuki, Okabe, Naoyuki, Matsumura, Yuki, Hasegawa, Takeo, Shio, Yutaka, . . . Suzuki, Hiroyuki. (2020). Successful Treatment of Combined Large Cell Neuroendocrine Carcinoma Harboring an EGFR Mutation with EGFR-TKIs plus Bevacizumab: A Case Report. Case reports in oncology, 13(3), 1387-1392. doi:10.1159/000511112
Ohba, Taro, Toyokawa, Gouji, Osoegawa, Atsushi, Hirai, Fumihiko, Yamaguchi, Masafumi, Taguchi, Ken-ichi, . . . Sugio, Kenji. (2016). Mutations of the EGFR, K-ras, EML4-ALK, and BRAF genes in resected pathological stage I lung adenocarcinoma. Surgery Today, 46(9), 1091-1098. doi:10.1007/s00595-015-1295-z
Ohtaki, Yoichi, Shimizu, Kimihiro, Kakegawa, Seiichi, Nagashima, Toshiteru, Nakano, Tetsuhiro, Atsumi, Jun, . . . Takeyoshi, Izumi. (2014). Postrecurrence survival of surgically resected pulmonary adenocarcinoma patients according to EGFR and KRAS mutation status. Molecular and clinical oncology, 2(2), 187-196. 
Oiwa, Hiroaki, Aokage, Keiju, Suzuki, Ayako, Sato, Kei, Kuroe, Takashi, Mimaki, Sachiyo, . . . Ishii, Genichiro. (2021). Clinicopathological, gene expression and genetic features of stage I lung adenocarcinoma with necrosis. Lung Cancer, 159, 74-83. doi:10.1016/j.lungcan.2021.07.001
Okamoto, Tatsuro, Kohno, Mikihiro, Ito, Kensaku, Takada, Kazuki, Katsura, Masakazu, Morodomi, Yosuke, . . . Maehara, Yoshihiko. (2017). Clinical Significance of DNA Damage Response Factors and Chromosomal Instability in Primary Lung Adenocarcinoma. Anticancer Research, 37(4), 1729-1735. doi:10.21873/anticanres.11505
Osawa, Junichiro, Shimada, Yoshihisa, Maehara, Sachio, Hagiwara, Masaru, Kakihana, Masatoshi, Kajiwara, Naohiro, . . . Ikeda, Norihiko. (2021). Clinical usefulness of the 3-tier classification according to the proportion of morphological patterns for patients with pathological stage I invasive lung adenocarcinoma. General Thoracic and Cardiovascular Surgery, 69(6), 943-949. doi:10.1007/s11748-020-01559-0
Otsuka, Tomoyuki, Tanaka, Aya, Azukizawa, Hiroaki, Sasaki, Shoko, Ishijima, Mikako, Matsuki, Takanori, . . . Kijima, Takashi. (2017). Successful treatment with gefitinib after Stevens-Johnson syndrome associated with afatinib therapy in a patient with adenocarcinoma of the lung. International cancer conference journal, 6(1), 38-41. doi:10.1007/s13691-016-0269-3
Paik, Jin Ho, Choi, Chang-Min, Kim, Hyojin, Jang, Se Jin, Choe, Gheeyoung, Kim, Dong Kwan, . . . Chung, Jin-Haeng. (2012). Clinicopathologic implication of ALK rearrangement in surgically resected lung cancer A proposal of diagnostic algorithm for ALK-rearranged adenocarcinoma. Lung Cancer, 76(3), 403-409. doi:10.1016/j.lungcan.2011.11.008
Pakvisal, Nussara, Chantranuwat, Poonchavist, Vinayanuwattikun, Chanida, Sitthideatphaiboon, Piyada, Teerapakpinyo, Chinachote, Shuangshoti, Shanop, . . . Sriuranpong, Virote. (2022). Prognostic factors in completely resected lymph-node-negative pulmonary adenocarcinoma. Translational Cancer Research, 11(7), 2238-+. doi:10.21037/tcr-21-2633
Park, In Kyu, Hyun, Kwanyong, Kim, Eung Re, Park, Samina, Kang, Chang Hyun, & Kim, Young Tae. (2018). The prognostic effect of the epidermal growth factor receptor gene mutation on recurrence dynamics of lung adenocarcinoma. European Journal of Cardio-Thoracic Surgery, 54(6), 1022-1027. doi:10.1093/ejcts/ezy220
Pedro, Brian Aram. (2020). Defining the Clinical and Biological Relevance of Leader and Follower Cell Mutations in Collective Cancer Invasion.
Pezzuto, Aldo, Terzo, Fabrizio, Graziani, Maria Laura, Ricci, Alberto, Bruno, Pierdonato, & Mariotta, Salvatore. (2017). Lung cancer requires multidisciplinary treatment to improve patient survival: A case report. Oncology Letters, 14(3), 3035-3038. doi:10.3892/ol.2017.6511
Press, Robert H., Zhang, Chao, Cassidy, Richard J., Ferris, Matthew J., Zhong, Jim, Steuer, Conor E., . . . Higgins, Kristin A. (2018). Targeted sequencing and intracranial outcomes of patients with lung adenocarcinoma brain metastases treated with radiotherapy. Cancer, 124(17), 3586-3595. doi:10.1002/cncr.31589
Qu, Rirong, Ye, Fan, Tu, Dehao, Cai, Yixin, & Fu, Xiangning. (2022). Clinical Features and Surgical Treatment of Synchronous Multiple Primary Lung Adenocarcinomas With Different EGFR Mutations. Frontiers in Oncology, 11. doi:10.3389/fonc.2021.785777
Ricaurte, Luisa Maria, Arrieta, Oscar, Zatarain-Barron, Zyanya Lucia, & Cardona, Andres F. (2018). Comprehensive review of fetal adenocarcinoma of the lung. Lung Cancer (Auckland, N.Z.), 9, 57-63. doi:10.2147/lctt.S137410
Sakai, Asao, Kasahara, Kazuo, & Sone, Takashi. (2013). Detection of EGFR T790M Mutation in Pericardial Effusion from a Non-Small Cell Lung Cancer Patient with Erlotinib Therapy. Case reports in oncology, 6(1), 15-20. doi:10.1159/000345947
Sakai, Hiroki, Kimura, Hiroyuki, Otsubo, Kanji, Miyazawa, Tomoyuki, Marushima, Hideki, Kojima, Koji, . . . Saji, Hisashi. (2022). Minichromosome maintenance 2 is an independent predictor of survival in patients with lung adenocarcinoma. Molecular and clinical oncology, 16(1), 22-22. doi:10.3892/mco.2021.2455
Sakatani, Toshio, Maemura, Keita, Hiyama, Noriko, Amano, Yosuke, Watanabe, Kousuke, Kage, Hidenori, . . . Takai, Daiya. (2017). High expression of IRE1 in lung adenocarcinoma is associated with a lower rate of recurrence. Japanese Journal of Clinical Oncology, 47(6), 543-550. doi:10.1093/jjco/hyx031
Scoccianti, Chiara, Vesin, Aurelien, Martel, Ghislaine, Olivier, Magali, Brambilla, Elisabeth, Timsit, Jean-Francois, . . . European Early Lung, Canc. (2012). Prognostic value of TP53, KRAS and EGFR mutations in nonsmall cell lung cancer: the EUELC cohort. European Respiratory Journal, 40(1), 177-184. doi:10.1183/09031936.00097311
Sharpnack, Michael Frederick. (2018). Integrative Genomics Methods for Personalized Treatment of Non-Small-Cell LungCancer.
Shen, Leilei, Lin, Jixing, Wang, Bailin, Xu, Hengliang, Zhao, Kai, & Zhang, Lianbin. (2019). Computed tomography findings, clinicopathological features, genetic characteristics and prognosis of in situ and minimally invasive lung adenocarcinomas. Nan fang yi ke da xue xue bao = Journal of Southern Medical University, 39(9), 1107-1112. doi:10.12122/j.issn.1673-4254.2019.09.17
Shim, Hyo Sup, Mari, Kenudson, Zheng, Zongli, Liebers, Matthew, Cha, Yoon Jin, Ho, Quan Hoang, . . . Iafrate, A. John. (2015). Unique Genetic and Survival Characteristics of Invasive Mucinous Adenocarcinoma of the Lung. Journal of Thoracic Oncology, 10(8), 1156-1162. doi:10.1097/jto.0000000000000579
Shimizu, Katsuhiko, Hirami, Yuji, Saisho, Shinsuke, Yukawa, Takuro, Maeda, Ai, Yasuda, Koichiro, & Nakata, Masao. (2012). Membrane-bound estrogen receptor-alpha expression and epidermal growth factor receptor mutation are associated with a poor prognosis in lung adenocarcinoma patients. World Journal of Surgical Oncology, 10. doi:10.1186/1477-7819-10-141
Song, Ruiqiang, Cheng, Yanbo, & Zheng, Tianxi. (2022). The Effect of Gefitinib on Treatment Necessity and Prognosis of NSCLC Patients with Early EGFR Mutations. Contrast Media & Molecular Imaging, 2022. doi:10.1155/2022/2228744
Song, Zhengbo, Lv, Tangfeng, Zhang, Yiping, & Song, Yong. (2017). Frequencies of actionable mutations and survival in variants of invasive adenocarcinoma of lung. Translational Cancer Research, 6(6), 1162-1169. doi:10.21037/tcr.2017.11.19
Sonoda, Dai, Matsuura, Yosuke, Kondo, Yasuto, Ichinose, Junji, Nakao, Masayuki, Ninomiya, Hironori, . . . Mun, Mingyon. (2022). A Reasonable Definition of Oligo-Recurrence in Non-Small-Cell Lung Cancer. Clinical Lung Cancer, 23(1), 82-90. doi:10.1016/j.cllc.2021.10.013
Stahl, John M., Walther, Zenta, Chang, Bryan W., Hochster, Howard S., & Johung, Kimberly L. (2017). A Long-Term Survivor of Metastatic Pancreatic Adenocarcinoma: Free of Recurrence 12 Years After Treatment of Oligometastatic Disease. Cureus, 9(2), e1007-e1007. doi:10.7759/cureus.1007
Suh, Young Joo, Lee, Hyun-Ju, Kim, Young Tae, Kang, Chang Hyun, Park, In Kyu, Jeon, Yoon Kyung, & Chung, Doo Hyun. (2018). Added prognostic value of CT characteristics and IASLC/ATS/ERS histologic subtype in surgically resected lung adenocarcinomas. Lung Cancer, 120, 130-136. doi:10.1016/j.lungcan.2018.04.007
Sun, P. L., Liu, J. N., Cao, L. Q., Yao, M., & Gao, H. W. (2017). To evaluate the clinicopathologic characteristics and outcome of tumor cells spreading through air spaces in patients with adenocarcinoma of lung. Zhonghua bing li xue za zhi = Chinese journal of pathology, 46(5), 303-308. doi:10.3760/cma.j.issn.0529-5807.2017.05.004
Takamochi, Kazuya, Oh, Shiaki, Matsunaga, Takeshi, & Suzuki, Kenji. (2017). Prognostic impacts of EGFR mutation status and subtype in patients with surgically resected lung adenocarcinoma. Journal of Thoracic and Cardiovascular Surgery, 154(5), 1768-+. doi:10.1016/j.jtcvs.2017.06.062
Tanaka, Hidenori, Okamoto, Takashi, Shinyama, Mami, Matsumura, Saeko, & Fujii, Tatsuo. (2013). A case of lung adenocarcinoma wtih exon19 and T790M mutations in EGFR having good response to erlotinib after gefitinib treatment failure. Gan to kagaku ryoho. Cancer & chemotherapy, 40(8), 1067-1069. 
Tanaka, Kazumi, Shimizu, Kimihiro, Kakegawa, Seiichi, Ohtaki, Yoichi, Nagashima, Toshiteru, Kaira, Kyoichi, . . . Takeyoshi, Izumi. (2016). Prognostic significance of aromatase and estrogen receptor beta expression in EGFR wild-type lung adenocarcinoma. American Journal of Translational Research, 8(1), 81-97. 
Timsah, Zahra, Berrout, Jonathan, Suraokar, Milind, Behrens, Carmen, Song, Juhee, Lee, J. Jack, . . . Ladbury, John E. (2015). Expression pattern of FGFR2, Grb2 and Plc gamma 1 acts as a novel prognostic marker of recurrence recurrence-free survival in lung adenocarcinoma. American Journal of Cancer Research, 5(10), 3135-+. 
Togashi, Yosuke, Masago, Katsuhiro, Hamatani, Yasuhiro, Sakamori, Yuichi, Nagai, Hiroki, Kim, Young Hak, & Mishima, Michiaki. (2012). Successful erlotinib rechallenge for leptomeningeal metastases of lung adenocarcinoma after erlotinib-induced interstitial lung disease: A case report and review of the literature. Lung Cancer, 77(2), 464-468. doi:10.1016/j.lungcan.2012.04.013
Tsunezuka, Yoshio, Tanaka, Nobuyoshi, Fujimori, Hideki, Togashi, Yuki, Baba, Satoko, Takeuchi, Kengo, . . . Yano, Seiji. (2017). The case of double primary lung adenocarcinomas with an EGFR mutation and ALK translocation successfully treated with alectinib at the post-surgical recurrence. The journal of medical investigation : JMI, 64(3.4), 305-307. doi:10.2152/jmi.64.305
Ueda, Daisuke, Ito, Masaoki, Tsutani, Yasuhiro, Gimenez-Capitan, Ana, Roman-Llado, Ruth, Perez-Rosado, Ana, . . . Okada, Morihito. (2021). Comprehensive analysis of the clinicopathological features, targetable profile, and prognosis of mucinous adenocarcinoma of the lung. Journal of Cancer Research and Clinical Oncology, 147(12), 3709-3718. doi:10.1007/s00432-021-03609-3
Ueda, Masatomo, Namba, Masashi, Tokumo, Kentaro, Senoo, Tadashi, Okamoto, Wataru, Yamauchi, Masami, . . . Sugiyama, Kazuhiko. (2021). Conversion from Positive to Negative EGFR Mutation due to Clonal Selection during Long-Term Treatment with Epidermal Growth Factor Receptor-Tyrosine Kinase Inhibitors: A Case Report. Case reports in oncology, 14(3), 1447-1453. doi:10.1159/000518246
Vaz, Daniel, Conde, Sara, Tente, David, Machado, Jose Carlos, & Barroso, Ana. (2017). Role of epidermal growth factor mutational status for distinction between recurrent lung cancer and second primary lung cancer: case report. Clinical Respiratory Journal, 11(6), 854-858. doi:10.1111/crj.12427
Villaruz, Liza C., Socinski, Mark A., Cunningham, Diana E., Chiosea, Simion I., Burns, Timothy F., Siegfried, Jill M., & Dacic, Sanja. (2013). The prognostic and predictive value of KRAS oncogene substitutions in lung adenocarcinoma. Cancer, 119(12), 2268-2274. doi:10.1002/cncr.28039
Voutsadakis, Ioannis A., & Mozarowski, Paul. (2017). Expression of TTF-1 in breast cancer independently of ER expression: A case report and pathogenic implications. Breast disease, 37(1), 1-6. doi:10.3233/bd-160240
Wang, Bo, & Jin, Huri. (2022). A case report of low grade fetal lung adenocarcinoma with TP53 mutation. Medicine, 101(11). doi:10.1097/md.0000000000029047
Wang, Kun, Xue, Mengchao, Qiu, Jianhao, Liu, Ling, Wang, Yueyao, Li, Rongyang, . . . Tian, Hui. (2022). Genomics Analysis and Nomogram Risk Prediction of Occult Lymph Node Metastasis in Non-Predominant Micropapillary Component of Lung Adenocarcinoma Measuring <= 3 cm. Frontiers in Oncology, 12. doi:10.3389/fonc.2022.945997
Wang, Rui, Zhang, Yang, Pan, Yunjian, Li, Yuan, Hu, Haichuan, Cai, Deng, . . . Chen, Haiquan. (2015). Comprehensive investigation of oncogenic driver mutations in Chinese non-small cell lung cancer patients. Oncotarget, 6(33), 34300-34308. doi:10.18632/oncotarget.5549
Wang, Yucong, Xu, Yinghui, Wang, Xu, Sun, Chao, Guo, Ye, Shao, Guoguang, . . . Ma, Kewei. (2019). RET fusion in advanced non-small-cell lung cancer and response to cabozantinib A case report. Medicine, 98(3). doi:10.1097/md.0000000000014120
Wang, Yadong, Yang, Xiaoying, Tian, Xu, Jia, Ziqi, Bing, Zhongxing, Cao, Lei, . . . Liang, Naixin. (2020). Neoadjuvant immunotherapy plus chemotherapy achieved pathologic complete response in stage IIIB lung adenocarcinoma harbored EGFR G779F: a case report. Annals of Palliative Medicine, 9(6), 4339-4345. doi:10.21037/apm-20-1692
Watanabe, Ayako, Inada, Ryo, Nagasaka, Takeshi, Yagi, Tomohiko, Matsumoto, Hijiri, Toshima, Toshiaki, . . . Fujiwara, Toshiyoshi. (2015). A successful multimodality therapy for a case of recurrent rectal cancer with KRAS mutation. Gan to kagaku ryoho. Cancer & chemotherapy, 42(2), 237-239. 
Watanabe, Hajime, Saito, Haruhiro, Yokose, Tomoyuki, Sakuma, Yuji, Murakami, Shuji, Kondo, Tetsuro, . . . Iwazaki, Masayuki. (2015). Relation Between Thin-Section Computed Tomography and Clinical Findings of Mucinous Adenocarcinoma. Annals of Thoracic Surgery, 99(3), 975-982. doi:10.1016/j.athoracsur.2014.10.065
Watanabe, Masato, Yokose, Tomoyuki, Tetsukan, Woo, Imai, Kentaro, Tsuboi, Masahiro, Ito, Hiroyuki, . . . Fujino, Shozo. (2013). Micropapillary components in a lung adenocarcinoma predict stump recurrence 8 years after resection: A case report. Lung Cancer, 80(2), 230-233. doi:10.1016/j.lungcan.2013.01.011
Wu, De-Wei, Wu, Tzu-Chin, Chen, Chih-Yi, & Lee, Huei. (2016). PAK1 Is a Novel Therapeutic Target in Tyrosine Kinase Inhibitor-Resistant Lung Adenocarcinoma Activated by the PI3K/AKT Signaling Regardless of EGFR Mutation. Clinical Cancer Research, 22(21), 5370-5382. doi:10.1158/1078-0432.Ccr-15-2724
Wu, Xiaoxuan, Song, Peng, Guo, Lei, Ying, Jianming, & Li, Wenbin. (2022). Mutant-Allele Tumor Heterogeneity, a Favorable Biomarker to Assess Intra-Tumor Heterogeneity, in Advanced Lung Adenocarcinoma. Frontiers in Oncology, 12. doi:10.3389/fonc.2022.888951
Wu, Yimin, Zhu, Zhouyu, Chen, Yongyuan, & Chai, Ying. (2017). Tonsillar metastasis of nonsmall cell lung cancer with G719S mutation in exon 18 A case report. Medicine, 96(49). doi:10.1097/md.0000000000009003
Xu, Shi-Bin, Xie, Ming-Ran, Li, Cai-Wei, Wu, Xian-Ning, & Xu, Mei-Qing. (2019). Correlation of pulmonary venous circulating tumor cells with clinicopathological parameters in patients with early-stage lung adenocarcinoma. Translational Cancer Research, 8(3), 887-898. doi:10.21037/tcr.2019.05.19
Xu, Ye, Zhu, Chen, Qian, Wenliang, & Zheng, Min. (2017). Comprehensive study of mutational and clinicopathologic characteristics of adenocarcinoma with lepidic pattern in surgical resected lung adenocarcinoma. Journal of Cancer Research and Clinical Oncology, 143(1), 181-186. doi:10.1007/s00432-016-2255-8
Yang, Shifeng, Song, Zhengbo, & Cheng, Guoping. (2019). Genomic alterations and survival in young patients aged under 40 years with completely resected non-small cell lung cancer. Annals of Translational Medicine, 7(7). doi:10.21037/atm.2019.03.39
Yang, Weixiong, You, Na, Jia, Minghan, Yeung, Sai-Ching Jim, Ou, Wei, Yu, Man, . . . Cheng, Chao. (2020). Undetectable circulating tumor DNA levels correlate with low risk of recurrence/metastasis in postoperative pathologic stage I lung adenocarcinoma patients. Lung Cancer, 146, 327-334. doi:10.1016/j.lungcan.2020.06.009
Yoshida, Tatsuya, Ishii, Genichiro, Goto, Koichi, Yoh, Kiyotaka, Niho, Seiji, Umemura, Shigeki, . . . Ochiai, Atsushi. (2013). Solid predominant histology predicts EGFR tyrosine kinase inhibitor response in patients with EGFR mutation-positive lung adenocarcinoma. Journal of Cancer Research and Clinical Oncology, 139(10), 1691-1700. doi:10.1007/s00432-013-1495-0
Yotsukura, Masaya, Asamura, Hisao, Motoi, Noriko, Kashima, Jumpei, Yoshida, Yukihiro, Nakagawa, Kazuo, . . . Watanabe, Shun-ichi. (2021). Long-Term Prognosis of Patients With Resected Adenocarcinoma In Situ and Minimally Invasive Adenocarcinoma of the Lung. Journal of Thoracic Oncology, 16(8), 1312-1320. doi:10.1016/j.jtho.2021.04.007
Yu, Su, Zhang, Yang, Pan, Yunjian, Cheng, Chao, Sun, Yihua, & Chen, Haiquan. (2017). The non-small cell lung cancer EGFR extracellular domain mutation, M277E, is oncogenic and drug-sensitive. Oncotargets and Therapy, 10, 4507-4515. doi:10.2147/ott.S131999
Zaric, Bojan, Brcic, Luka, Buder, Anna, Brandstetter, Anita, Buresch, Jorun O., Traint, Stefan, . . . Filipits, Martin. (2018). PD-1 and PD-L1 Protein Expression Predict Survival in Completely Resected Lung Adenocarcinoma. Clinical Lung Cancer, 19(6), E957-E963. doi:10.1016/j.cllc.2018.08.014
Zhang, Chu, Wang, Xiang, Zhang, Miao, Liu, Dong, & Yang, Dun-Peng. (2020). Neoadjuvant apatinib plus S-1 in locally advanced pulmonary adenocarcinoma A case report and review of the literature. Medicine, 99(3). doi:10.1097/md.0000000000018767
Zhang, Xilin, Jiang, Yan, Yu, Huanming, Xia, Hui, & Wang, Xiang. (2020). A comprehensive study on the oncogenic mutation and molecular pathology in Chinese lung adenocarcinoma patients. World Journal of Surgical Oncology, 18(1). doi:10.1186/s12957-020-01947-z
Zhang, Yang, Li, Jing, Wang, Rui, Li, Yuan, Pan, Yunjian, Cai, Deng, . . . Chen, Haiquan. (2014). The prognostic and predictive value of solid subtype in invasive lung adenocarcinoma. Scientific Reports, 4. doi:10.1038/srep07163
Zhang, Yiliang, Ma, Yuan, Li, Yuan, Shen, Xuxia, Yu, Yongfu, Pan, Yunjian, . . . Chen, Haiquan. (2018). Are exon 19 deletions and L858R different in early stage lung adenocarcinoma? Journal of Cancer Research and Clinical Oncology, 144(1), 165-171. doi:10.1007/s00432-017-2526-z
Zhang, Yiliang, Ma, Yuan, Li, Yuan, Shen, Xuxia, Yu, Yongfu, Pan, Yunjian, . . . Chen, Haiquan. (2019). Comparative analysis of co-occurring mutations of specific tumor suppressor genes in lung adenocarcinoma between Asian and Caucasian populations. Journal of Cancer Research and Clinical Oncology, 145(3), 747-757. doi:10.1007/s00432-018-02828-5
Zhang, Yang, Wang, Rui, Cai, Deng, Li, Yuan, Pan, Yunjian, Hu, Haichuan, . . . Chen, Haiquan. (2014). A Comprehensive Investigation of Molecular Features and Prognosis of Lung Adenocarcinoma with Micropapillary Component. Journal of Thoracic Oncology, 9(12), 1772-1778. doi:10.1097/jto.0000000000000341
Zhang, Yiliang, Wang, Rui, Li, Yuan, Pan, Yunjian, Hu, Haichuan, Zhang, Yang, . . . Chen, Haiquan. (2015). Negative Thyroid Transcription Factor 1 Expression Defines an Unfavorable Subgroup of Lung Adenocarcinomas. Journal of Thoracic Oncology, 10(10), 1444-1450. doi:10.1097/jto.0000000000000626
Zhao, Yue, Han, Han, Gao, Zhendong, Hu, Hong, Xiang, Jiaqing, Sun, Yihua, & Chen, Haiquan. (2021). Evolutionary Action Score of TP53 Enhances the Prognostic Prediction for Stage I Lung Adenocarcinoma. Seminars in Thoracic and Cardiovascular Surgery, 33(1), 221-229. doi:10.1053/j.semtcvs.2020.04.005
Zhao, Yue, Pan, Yunjian, Cheng, Chao, Zheng, Difan, Zhang, Yang, Gao, Zhendong, . . . Chen, Haiquan. (2020). EGFR-mutant lung adenocarcinoma harboring co-mutational tumor suppressor genes predicts poor prognosis. Journal of Cancer Research and Clinical Oncology, 146(7), 1781-1789. doi:10.1007/s00432-020-03237-3
Zhao, Yanding, Varn, Frederick S., Cai, Guoshuai, Xiao, Feifei, Amos, Christopher I., & Cheng, Chao. (2018). A P53-Deficiency Gene Signature Predicts Recurrence Risk of Patients with Early-Stage Lung Adenocarcinoma. Cancer Epidemiology Biomarkers & Prevention, 27(1), 86-95. doi:10.1158/1055-9965.Epi-17-0478
Zheng, Difan, Wang, Rui, Pan, Yunjian, Zheng, Shanbo, Zhang, Yang, Li, Hang, . . . Chen, Haiquan. (2015). Prevalence and Clinicopathological Characteristics of BRAF Mutations in Chinese Patients with Lung Adenocarcinoma. Annals of Surgical Oncology, 22, S1284-S1291. doi:10.1245/s10434-015-4640-y
Zhou, Tao, Xiong, Qiang, Hong, Chen, Wang, Qian, Wang, Wenxian, Xu, Chunwei, . . . Yangtze River Delta Lung Canc, Coop. (2022). A novel EGFR exon 21 indel mutation in lung adenocarcinoma and response to dacomitinib: A case report. Medicine, 101(34). doi:10.1097/md.0000000000030269
Zhou, Xiuzhi, Cai, Li, Liu, Junjie, Hua, Xiaomin, Zhang, Ying, Zhao, Huilin, . . . Gai, Pengzhou. (2018). Analyzing EGFR mutations and their association with clinicopathological characteristics and prognosis of patients with lung adenocarcinoma. Oncology Letters, 16(1), 362-370. doi:10.3892/ol.2018.8681
Zhu, Shuai, Zhao, Shikang, Zhang, Qian, Li, Shuo, Ren, Dian, Ren, Fan, . . . Xu, Song. (2021). Complete disease remission in a TP53 and KRAS co-mutated brain oligometastatic lung cancer patient after immuno-chemotherapy and surgical resection: a case report. Translational Lung Cancer Research, 10(5), 2298-2305. doi:10.21037/tlcr-21-380
