# Supplementary material for: Prognosis of recurrence after complete resection in early-stage lung adenocarcinoma based on molecular alterations: a systematic review and meta-analysis
Source: Sci Rep. 2023 Oct 31;13:18710. doi: 10.1038/s41598-023-42851-2 (PMC10618289; doi:10.1038/s41598-023-42851-2)
Supplement: Supplementary file 2 — Supplementary Information 2. [file 41598_2023_42851_MOESM2_ESM.pdf]

Prognosis of Recurrence After Complete Resection in Early-stage Lung Adenocarcinoma Based on Molecular Alterations: A Systematic Review and Meta-analysis (Supplementary Materials)

Chu Zhou[1], Zhongying Jing[2], Wei Liu[2], Zihuan Ma[2], Siyao Liu[2], Yueyu Fang[3*]

[1]Department of Thoracic Surgery, Nanjing Drum Tower Hospital, Medical School, Nanjing University, Nanjing 210008, China.

[2] Beijing ChosenMed Clinical Laboratory Co. Ltd., Beijing 100176, China.

[3]Department of Medical Oncology, Nanjing PuKou People's Hospital, Nanjing 211800, China.

*** Correspondence:**

Yueyu Fang

Fyymmy091120@163.com

Supplementary Table S1. Key considerations in study risk of bias assessment (QUIPS tool)

	Study Participation	Study Attrition	Prognostic Factor Measurement	Outcome Measurement	Study Confounding	Statistical Analysis and Reporting
**Aokage et al, 2021 (18)**	✓ Participant description*<br>✓ Sampling frame hospital and community based<br>✓Adequate participation in the study by eligible persons<br>✓ LUAD characteristics†<br>✓ Recruitment time between January 2003 and December 2014<br><br>✕ Unclear inclusion and exclusion criteria<br>✕ Single-center<br>✕ Retrospective	✓ Adequate response rate<br><br>✕ Unclear rate and reasons of participants lost to follow-up<br>✕ Unclear attempts to collect information on participants lost to follow-up	✓ Clear description of PF<br>✓ Method and setting of PF measurement same for all participants<br>✓ Reliable method of PF<br><br>✕ Some samples without complete data for the PF	✓ Clear definition of the outcome<br>✓ Good follow-up (The proportion of post-operative follow-up was more than 98%)<br><br>✕ Unclear method of follow-up<br>✕ Method and setting of outcome measurement for all participants was unclear	✓ Important confounders are measured‡<br>✓ Clear differences in follow-up intensity by recurrent risk<br><br>✕ Unclear differences in systemic therapy in LUAD samples	✓ Reported distributions of available key variables<br>✓ KM survival curves<br>✓ The selected statistical model is adequate for the design of the study
**Deng et al, 2020 (30)**	✓ Participant description*<br>✓ Sampling frame hospital and community based<br>✓Adequate participation in the study by eligible persons<br>✓ LUAD characteristics†<br>✓ Recruitment time between January 2008 and March 2015<br>✓ Clear inclusion and exclusion criteria<br><br>✕ Retrospective<br>✕ Single-center	✓ Adequate response rate<br><br>✕ Unclear rate and reasons of participants lost to follow-up<br>✕ Unclear attempts to collect information on participants lost to follow-up	✓ Clear description of PF<br>✓ Method and setting of PF measurement same for all participants<br>✓ Reliable method of PF<br><br>✕ Participants without successful PF test not described	✓ Clear definition of the outcome<br>✓ Clear method of follow-up<br>✓ Good follow-up<br>✓ Method and setting of outcome measurement for all participants was clear	✓ Important confounders are measured‡<br><br>✕ Unclear differences in systemic therapy in LUAD samples	✓ Reported distributions of available key variables<br>✓ KM survival curves<br>✓ The selected statistical model is adequate for the design of the study<br><br>✕ selective reporting of results to some extent
**Hayasaka et al, 2018 (26)**	✓ Participant description*<br>✓ Sampling frame hospital and community based<br>✓Adequate participation in the study by eligible persons<br>✓ Multi-center<br>✓ LUAD characteristics†<br>✓ Recruitment time between January 2005 and December 2012<br>✓ Clear inclusion and exclusion criteria<br><br>✕ Retrospective	✓ Adequate response rate<br><br>✕ Unclear rate and reasons of participants lost to follow-up<br>✕ Unclear attempts to collect information on participants lost to follow-up	✓ Clear description of PF<br>✓ Reliable method of PF<br>✓ All samples has complete data for the PF<br><br>✕ Method and setting of PF measurement not same for all participants	✓ Clear definition of the outcome<br><br>✕ Unclear method of follow-up<br>✕ Method and setting of outcome measurement for all participants was unclear	✓ Important confounders are measured‡<br><br>✕ Unclear differences in systemic therapy in LUAD samples	✓ Reported distributions of available key variables<br>✓ KM survival curves<br>✓ The selected statistical model is adequate for the design of the study<br><br>✕ Unclear relation of all EGFR alterations to DFS/RFS
**Isaka et al, 2018 (27)**	✓ Participant description*<br>✓Adequate participation in the study by eligible persons<br>✓ Recruitment time between January 2002 and March 2016<br><br>✕ Retrospective<br>✕ Restricted sampling frame<br>✕ Restricted inclusion and exclusion criteria<br>✕ Restricted LUAD characteristics†	✓ Adequate response rate<br>✓ Adequate follow-up<br><br>✕ Unclear rate and reasons of participants lost to follow-up<br>✕ Unclear attempts to collect information on participants lost to follow-up	✓ Clear description of PF<br>✓ Reliable method of PF<br><br>✕ Participants without successful PF test not described<br>✕ Method and setting of PF measurement not same for all participants	✓ Clear definition of the outcome<br>✓ Clear method of follow-up<br>✓ Method and setting of outcome measurement for all participants was clear	✓ Important confounders are measured‡<br>✓ No differences in follow-up intensity by recurrent risk<br><br>✕ Unclear differences in systemic therapy in LUAD samples	✓ Reported distributions of available key variables<br>✓ KM survival curves<br>✓ The selected statistical model is adequate for the design of the study
**Ito et al, 2018 (20)**	✓ Participant description*<br>✓Adequate participation in the study by eligible persons<br>✓ Sampling frame hospital and community based<br>✓ Recruitment time between January 2007 and December 2013<br>✓ LUAD characteristics†<br>✓ Clear inclusion and exclusion criteria<br><br>✕ Retrospective<br>✕ Single-center	✓ Adequate response rate<br><br>✕Unclear rate and reasons of participants lost to follow-up<br>✕ Unclear attempts to collect information on participants lost to follow-up	✓ Clear description of PF<br>✓ Reliable method of PF<br>✓ Method and setting of PF measurement same for all participants<br><br>✕ Participants without successful PF test not described	✓ Clear definition of the outcome<br><br>✕ Unclear method of follow-up<br>✕ Method and setting of outcome measurement for all participants was unclear	✓ Important confounders are measured‡<br><br>✕ Unclear differences in systemic therapy in LUAD samples	✓ Reported distributions of available key variables<br>✓ KM survival curves<br>✓ The selected statistical model is adequate for the design of the study<br><br>✕ selective reporting of results to some extent

**Ito et al, 2020 (19)**	✓ Participant description*<br>✓Adequate participation in the study by eligible persons<br>✓ Multi-center<br>✓ Sampling frame hospital and community based<br>✓ LUAD characteristics†<br>✓ Recruitment time between January 2010 and December 2016<br>✓ Clear inclusion and exclusion criteria<br><br>✕ Retrospective	✓ Adequate response rate<br><br>✕ Unclear rate and reasons of participants lost to follow-up<br>✕ Unclear attempts to collect information on participants lost to follow-up	✓ Clear description of PF<br>✓ Reliable method of PF<br><br>✕ Participants without successful PF test not described<br>✕ Method and setting of PF measurement not same for all participants	✓ Clear definition of the outcome<br><br>✕ Unclear method of follow-up<br>✕ Method and setting of outcome measurement for all participants was unclear	✓ Important confounders are measured‡<br><br>✕ Unclear differences in systemic therapy in LUAD samples	✓ Reported distributions of available key variables<br>✓ KM survival curves<br>✓ The selected statistical model is adequate for the design of the study<br><br>✕ clearly selective reporting of results
**Izar et al, 2014 (28)**	✓ Participant description*<br>✓Adequate participation in the study by eligible persons<br>✓ Sampling frame hospital and community based<br>✓ Recruitment time between January 2008 and December 2011<br>✓ Clear inclusion and exclusion criteria<br><br>✕ Retrospective<br>✕ Single-center<br>✕ Restricted LUAD characteristics†	✓ Adequate response rate<br>✓ Adequate follow-up<br>✓ Clear rate and reasons of participants lost to follow-up<br><br>✕ Unclear attempts to collect information on participants lost to follow-up	✓ Clear description of PF<br>✓ Reliable method of PF<br>✓ Method and setting of PF measurement same for all participants<br><br>✕ Participants without successful PF test not described	✓ Clear definition of the outcome<br>✓ Clear method of follow-up<br>✓ Method and setting of outcome measurement for all participants was clear	✓ Important confounders are measured‡<br>✓ No differences in follow-up intensity by recurrent risk<br>✓ No differences in systemic therapy in LUAD samples	✓ Reported distributions of available key variables<br>✓ KM survival curves<br>✓ The selected statistical model is adequate for the design of the study
**Jones et al, 2021 (25)**	✓ Participant description*<br>✓Adequate participation in the study by eligible persons<br>✓ Sampling frame hospital and community based<br>✓ LUAD characteristics†<br>✓ Recruitment time between February 2010 and December 2018<br>✓ Clear inclusion and exclusion criteria<br>✓ Prospective<br><br>✕ Single-center	✓ Adequate response rate<br><br>✕ Unclear rate and reasons of participants lost to follow-up<br>✕ Unclear attempts to collect information on participants lost to follow-up	✓ Clear description of PF<br>✓ Reliable method of PF<br>✓ Method and setting of PF measurement same for all participants<br><br>✕ Participants without successful PF test not described	✓ Clear definition of the outcome<br><br>✕ Unclear method of follow-up<br>✕ Method and setting of outcome measurement for all participants was unclear	✓ Important confounders are measured‡<br><br>✕ Unclear differences in systemic therapy in LUAD samples	✓ Reported distributions of available key variables<br>✓ KM survival curves<br>✓ The selected statistical model is adequate for the design of the study<br><br>✕ selective reporting of results to some extent
**Kadota et al, 2016 (31)**	✓ Participant description*<br>✓Adequate participation in the study by eligible persons<br>✓ Sampling frame hospital and community based<br>✓ LUAD characteristics†<br>✓ Recruitment time between 1995 and 2005<br><br>✕ Retrospective<br>✕ Single-center<br>✕ Restricted inclusion and exclusion criteria	✓ Adequate response rate<br><br>✕ Unclear rate and reasons of participants lost to follow-up<br>✕ Unclear attempts to collect information on participants lost to follow-up	✓ Clear description of PF<br>✓ Reliable method of PF<br><br>✕ Participants without successful PF test not described<br>✕ Method and setting of PF measurement not same for all participants	✓ Clear definition of the outcome<br><br>✕ Unclear method of follow-up<br>✕ Method and setting of outcome measurement for all participants was unclear	✓ Important confounders are measured‡<br><br>✕ Unclear differences in systemic therapy in LUAD samples	✓ Reported distributions of available key variables<br>✓ KM survival curves<br>✓ The selected statistical model is adequate for the design of the study
**Kim et al, 2021 (14)**	✓ Participant description*<br>✓Adequate participation in the study by eligible persons<br>✓ Sampling frame hospital and community based<br>✓ Recruitment time between September 2005 to May 2017<br><br>✕ Retrospective<br>✕ Single-center<br>✕ Restricted LUAD characteristics†<br>✕ Restricted inclusion and exclusion criteria	✓ Adequate response rate<br><br>✕ Unclear rate and reasons of participants lost to follow-up<br>✕ Unclear attempts to collect information on participants lost to follow-up	✓ Clear description of PF<br>✓ Reliable method of PF<br>✓ Method and setting of PF measurement same for all participants<br><br>✕ Participants without successful PF test not described	✓ Clear definition of the outcome<br><br>✕ Unclear method of follow-up<br>✕ Method and setting of outcome measurement for all participants was unclear	✓ Important confounders are measured‡<br><br>✕ Unclear differences in systemic therapy in LUAD samples	✓ Reported distributions of available key variables<br>✓ The selected statistical model is adequate for the design of the study

**Kneuertz et al, 2020 (15)**	✓ Participant description*<br>✓Adequate participation in the study by eligible persons<br>✓ Sampling frame hospital and community based<br>✓ Recruitment time between October 2011 and September 2017<br><br>× Retrospective<br>× Single-center<br>× Restricted LUAD characteristics†<br>× Restricted inclusion and exclusion criteria	✓ Adequate response rate<br><br>× Unclear rate and reasons of participants lost to follow-up<br>× Unclear attempts to collect information on participants lost to follow-up	✓ Clear description of PF<br>✓ Reliable method of PF<br><br>× Participants without successful PF test not described<br>× Method and setting of PF measurement not same for all participants	✓ Clear definition of the outcome<br>✓ Setting of outcome measurement for all participants was clear<br><br>× Unclear method of follow-up<br>× Method of outcome measurement for all participants was unclear	✓ Important confounders are measured‡<br>✓ No differences in follow-up intensity by recurrent risk<br><br>× Clear differences in systemic therapy in LUAD samples	✓ Reported distributions of available key variables<br>✓ KM survival curves<br>✓ The selected statistical model is adequate for the design of the study
**Kondo et al, 2022 (21)**	✓ Participant description*<br>✓Adequate participation in the study by eligible persons<br>✓ Sampling frame hospital and community based<br>✓ LUAD characteristics†<br>✓ Recruitment time between January 2009 and December 2017<br>✓ Clear inclusion and exclusion criteria<br><br>× Retrospective<br>× Single-center	✓ Adequate response rate<br><br>× Unclear rate and reasons of participants lost to follow-up<br>× Unclear attempts to collect information on participants lost to follow-up	✓ Clear description of PF<br>✓ Reliable method of PF<br><br>× Participants without successful PF test not described<br>× Method and setting of PF measurement not same for all participants	✓ Clear definition of the outcome<br>✓ Clear method of follow-up<br>✓ Method and setting of outcome measurement for all participants was clear<br><br>× Method of outcome measurement not same for all participants	✓ Important confounders are measured‡<br>✓ No differences in follow-up intensity by recurrent risk<br><br>× Clear differences in systemic therapy in LUAD samples	✓ Reported distributions of available key variables<br>✓ KM survival curves<br>✓ The selected statistical model is adequate for the design of the study
**Li et al, 2018 (22)**	✓ Participant description*<br>✓Adequate participation in the study by eligible persons<br>✓ Sampling frame hospital and community based<br>✓ LUAD characteristics†<br>✓ Recruitment time between October 2007 and March 2013<br>✓ Clear inclusion and exclusion criteria<br><br>× Retrospective<br>× Single-center	✓ Adequate response rate<br><br>× Unclear rate and reasons of participants lost to follow-up<br>× Unclear attempts to collect information on participants lost to follow-up	✓ Clear description of PF<br>✓ Reliable method of PF<br>✓ Method and setting of PF measurement same for all participants<br><br>× Participants without successful PF test not described	✓ Clear method of follow-up<br><br>× Method and setting of outcome measurement for all participants was unclear<br>× Unclear definition of the outcome	✓ Important confounders are measured‡<br><br>× Unclear differences in follow-up intensity by recurrent risk<br>× Unclear differences in systemic therapy in LUAD samples	✓ Reported distributions of available key variables<br>✓ KM survival curves<br>✓ The selected statistical model is adequate for the design of the study<br><br>× selective reporting of results to some extent
**Ma et al, 2022 (23)**	✓ Participant description*<br>✓Adequate participation in the study by eligible persons<br>✓ Sampling frame hospital and community based<br>✓ LUAD characteristics†<br>✓ Recruitment time between November 2008 and March 2015<br>✓ Clear inclusion and exclusion criteria<br>✓ Prospective<br><br>× Single-center	✓ Adequate response rate<br><br>× Unclear rate and reasons of participants lost to follow-up<br>× Unclear attempts to collect information on participants lost to follow-up	✓ Clear description of PF<br>✓ Reliable method of PF<br>✓ Method and setting of PF measurement same for all participants<br><br>× Participants without successful PF test not described	✓ Clear definition of the outcome<br>✓ Clear method of follow-up<br>✓ Method and setting of outcome measurement for all participants was clear<br><br>× Method of outcome measurement not same for all participants	✓ Important confounders are measured‡<br>✓ No differences in follow-up intensity by recurrent risk<br><br>× Clear differences in systemic therapy in LUAD samples	✓ Reported distributions of available key variables<br>✓ KM survival curves<br>✓ The selected statistical model is adequate for the design of the study
**Matsumura et al, 2017 (29)**	✓ Participant description*<br>✓Adequate participation in the study by eligible persons<br>✓ Sampling frame hospital and community based<br>✓ LUAD characteristics†<br>✓ Recruitment time between Janury 2007 and December 2013<br>✓ Clear inclusion and exclusion criteria<br>✓ Multi-center<br><br>× Retrospective	✓ Adequate response rate<br><br>× Unclear rate and reasons of participants lost to follow-up<br>× Unclear attempts to collect information on participants lost to follow-up	✓ Clear description of PF<br>✓ Reliable method of PF<br><br>× Participants without successful PF test not described<br>× Method and setting of PF measurement not same for all participants	✓ Clear definition of the outcome<br>✓ Method of outcome measurement for all participants was clear<br><br>× Unclear method of follow-up<br>× Setting of outcome measurement for all participants was unclear<br>× Method of outcome measurement not same for all participants	✓ Important confounders are measured‡<br><br>× Unclear differences in follow-up intensity by recurrent risk<br>× Unclear differences in systemic therapy in LUAD samples	✓ Reported distributions of available key variables<br>✓ KM survival curves<br>✓ The selected statistical model is adequate for the design of the study

**Ohba et al, 2016 (24)**	✓ Participant description*<br>✓Adequate participation in the study by eligible persons<br>✓ Sampling frame hospital and community based<br>✓ Recruitment time between 2002 and 2006<br><br>╳ Single-center<br>╳ Retrospective<br>╳ Restricted LUAD characteristics†<br>╳ Restricted inclusion and exclusion criteria	✓ Adequate response rate<br><br>╳ Unclear rate and reasons of participants lost to follow-up<br>╳ Unclear attempts to collect information on participants lost to follow-up	✓ Clear description of PF<br>✓ Reliable method of PF<br><br>╳ Participants without successful PF test not described<br>╳ Method and setting of PF measurement not same for all participants	✓ Clear definition of the outcome<br><br>╳ Unclear method of follow-up<br>╳ Method and setting of outcome measurement for all participants was unclear	╳Restricted important confounders are measured‡<br>╳ Unclear differences in follow-up intensity by recurrent risk<br>╳ Unclear differences in systemic therapy in LUAD samples	✓ Reported distributions of available key variables<br>✓ KM survival curves<br>✓ The selected statistical model is adequate for the design of the study<br><br>╳ selective reporting of results to some extent
**Shimizu et al, 2017 (16)**	✓ Participant description*<br>✓Adequate participation in the study by eligible persons<br>✓ Sampling frame hospital and community based<br>✓ Recruitment time between June 2003 and April 2013<br><br>╳ Single-center<br>╳ Retrospective<br>╳ Restricted LUAD characteristics†<br>╳ Restricted inclusion and exclusion criteria	✓ Adequate response rate<br><br>╳ Unclear rate and reasons of participants lost to follow-up<br>╳ Unclear attempts to collect information on participants lost to follow-up	✓ Clear description of PF<br>✓ Reliable method of PF<br>✓ Method and setting of PF measurement same for all participants<br><br>╳ Participants without successful PF test not described	✓ Clear definition of the outcome<br>✓ Method and setting of outcome measurement for all participants was clear<br>✓ Method and setting of outcome measurement for all participants was same<br><br>╳ Unclear method of follow-up	╳Restricted important confounders are measured‡<br>╳ Unclear differences in follow-up intensity by recurrent risk<br>╳ Unclear differences in systemic therapy in LUAD samples	✓ Reported distributions of available key variables<br>✓ KM survival curves<br>✓ The selected statistical model is adequate for the design of the study
**Zhou et al, 2021 (13)**	✓ Participant description*<br>✓Adequate participation in the study by eligible persons<br>✓ Sampling frame hospital and community based<br>✓ Recruitment time between January 2018 and December 2018<br>✓ Clear inclusion and exclusion criteria<br><br>╳ Single-center<br>╳ Retrospective<br>╳ Restricted LUAD characteristics†	✓ Adequate response rate<br><br>╳ Unclear rate and reasons of participants lost to follow-up<br>╳ Unclear attempts to collect information on participants lost to follow-up	✓ Clear description of PF<br>✓ Reliable method of PF<br>✓ Method and setting of PF measurement same for all participants<br><br>╳ Participants without successful PF test not described	✓ Clear definition of the outcome<br>✓ Clear method of follow-up<br><br>╳ Method and setting of outcome measurement for all participants was unclear	✓ Important confounders are measured‡<br>✓ No differences in follow-up intensity by recurrent risk<br><br>╳ Clear differences in systemic therapy in LUAD samples	✓ Reported distributions of available key variables<br>✓ KM survival curves<br>✓ The selected statistical model is adequate for the design of the study<br><br>╳ selective reporting of results to some extent

*adequate reporting of age and sex

†adequate reporting of histology, pStage, smoking and mutation rate

‡ age, sex, histological subtype, smoking history, pStage

Abbreviations: LUAD, lung adenocarcinoma; PF, prognostic factor, surgery, neoadjuvant and adjuvant therapy

Supplementary Table S2 Clinical endpoint definitions for clinical researches

**Study**	**Biomarker**	**Outcome**	**Outcome definition**
Aokage et al, 2021 (18)	*EGFR*	RFS	The time from the date of surgery to the date of first recurrence, death from any cause, or last follow-up
Deng et al, 2021 (30)	*EGFR*	RFS	The time from the date of surgery to the date of first recurrence or last follow-up
Hayasaka et al, 2018 (26)	*EGFR*	RFS	The time from the date of surgery to the date of first recurrence, death due to any cause, or last follow-up
Isaka et al, 2018 (27)	*EGFR*	RFS	The time from the date of surgery to the date of first recurrence
Ito et al, 2020 (19)	*EGFR*	RFS	The time from the date of surgery to the date of first recurrence
Ito et al, 2018 (20)	*EGFR*	RFI	The time from the date of surgery to the date of first recurrence
Kim et al, 2021 (14)	*EGFR*/*KRAS*	RFS	The time from the date of surgery to the date of first recurrence or last follow-up
Kondo et al, 2022 (21)	*EGFR*	RFS	The time from the date of surgery to the date of first recurrence or death from any cause
Matsumura et al, 2017 (29)	*EGFR*	RFS	The time from the date of surgery to the date of first recurrence or last follow-up
Shimizu et al, 2017 (16)	*EGFR*/*KRAS*	DFP	The time from the date of surgery to the date of first recurrence
Zhou et al, 2021 (13)	*EGFR*	RFS	The time from the date of surgery to the date of first recurrence or last follow-up
Izar et al, 2014 (28)	*KRAS*	DFS	The time from the date of surgery to the date of first recurrence or death from any cause
Jones et al, 2021 (25)	*KRAS*	DFS	The time from the date of surgery to the date of first recurrence or death from any cause
Kadota et al, 2016 (31)	*KRAS*	CIR	The time from the date of surgery to the date of first recurrence, death due to any cause, or last follow-up
Kneuertz et al, 2020 (15)	*KRAS*	DFS	The time from the date of surgery to the date of biopsy-proven or radiologicl evidence of disease recurrence or death from any cause
Li et al, 2018 (22)	*KRAS*	DFS	NS
Ma et al, 2022 (23)	*KRAS*	RFS	The time from the date of surgery to the date of first recurrence or last negative follow-up.
Ohba et al, 2016 (24)	*KRAS*	DFS	The time from the date of surgery to the date of first recurrence

Abbreviations: CIR, cumulative incidence of recurrence; DFP, disease-free proportion; DFS, disease-free survival; RFI, recurrence-free interval; RFS, relapse-free survival; NS, not sure.

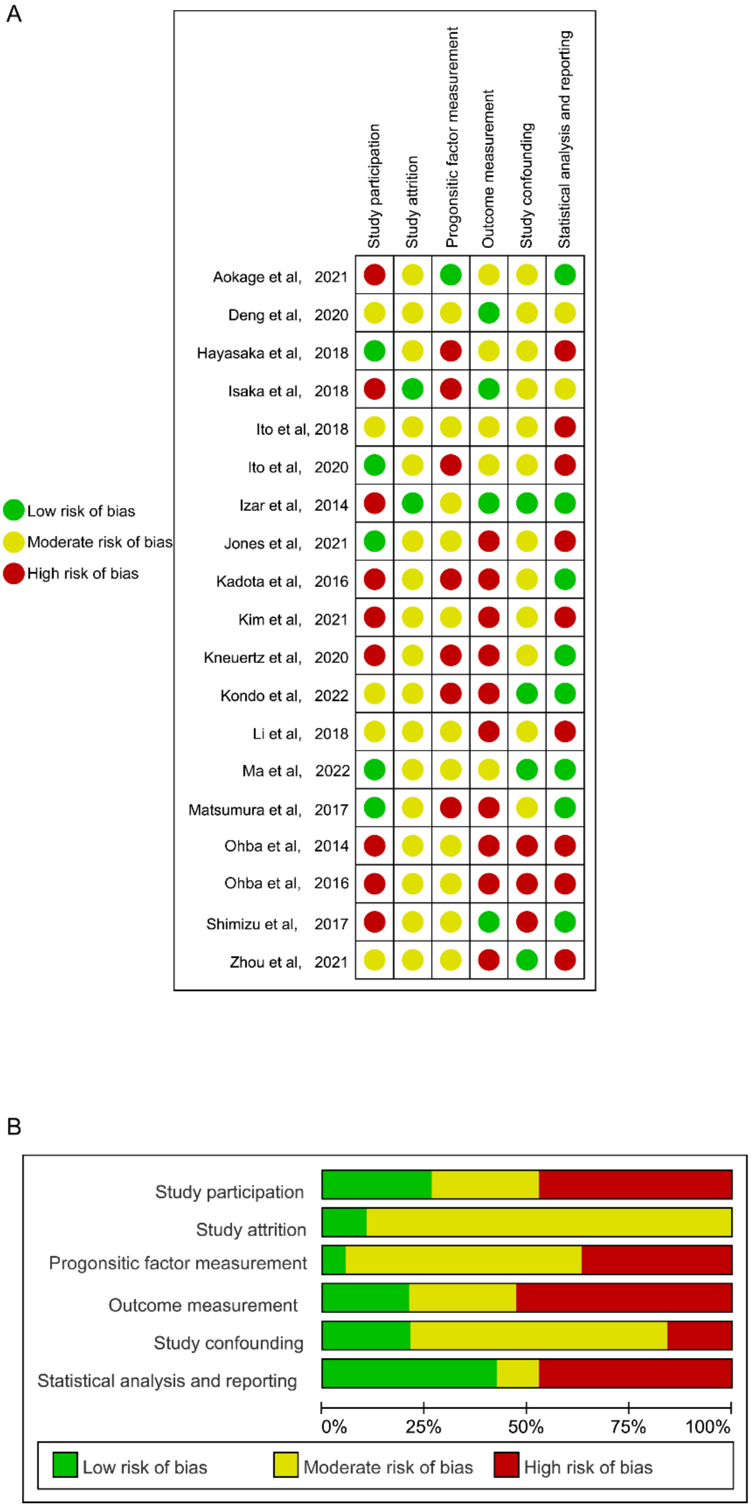

Supplementary Figure S1. Individual risk of bias.

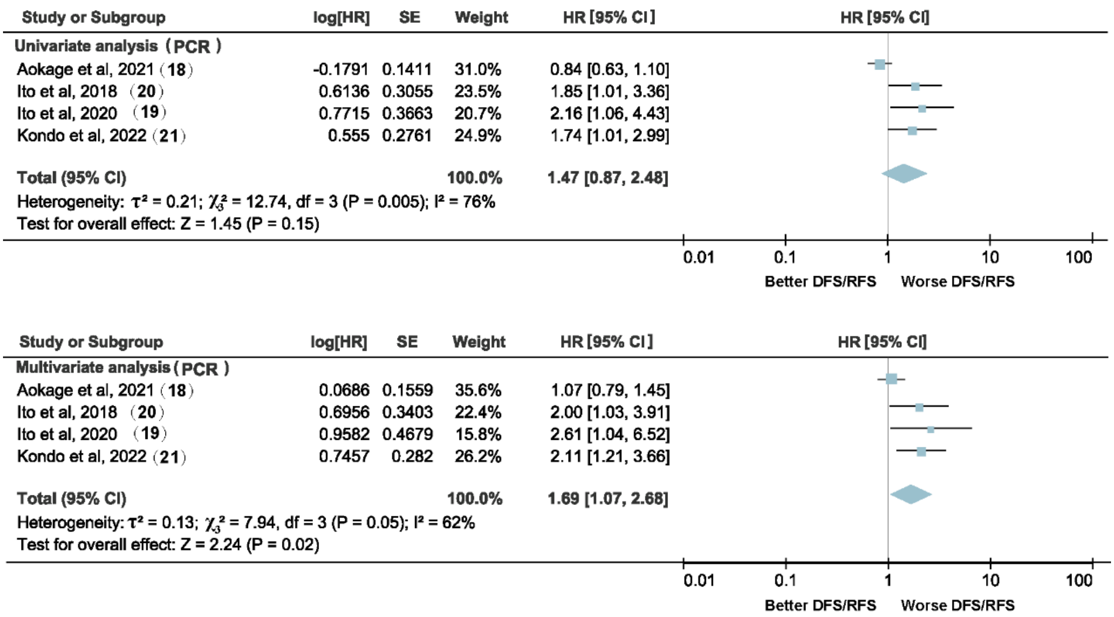

Supplementary Figure S2. Subgroup analysis of random-effects model for the association between *EGFR* mutation status and DFS/RFS in PCR platform. Upper section: Univariate analysis; Lower section: Multivariate analysis.

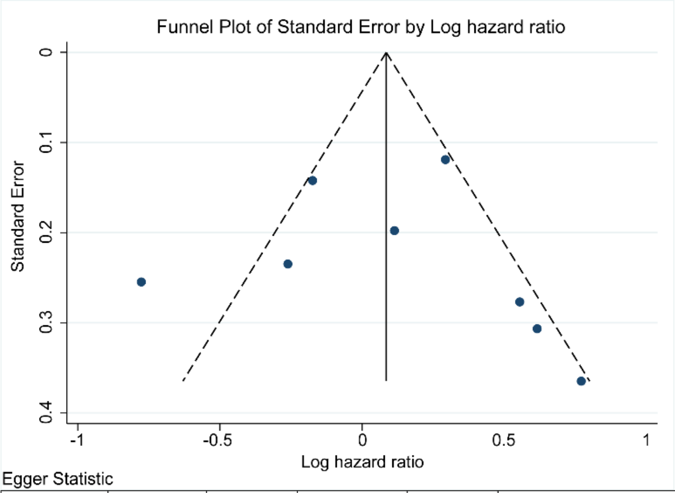

Egger Statistic

					95%CI	
Effect	Coefficient	Std.Err	t-statistic	P-value	Lower	Upper
Slope	-0.0081537	0.423117	-0.02	0.985	-1.043482	1.027175
Bias	0.5093169	2.169927	0.23	0.822	-4.800303	5.818937

Supplementary Figure S3. Funnel plot and Egger test of imputed trials in univariate analysis of *EGFR* mutation in early-stage LUAD

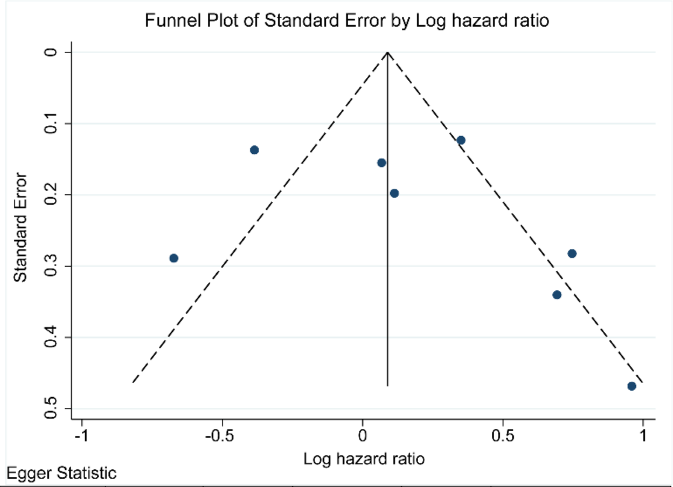

Egger Statistic

					95%CI	
Effect	Coefficient	Std.Err	t-statistic	P-value	Lower	Upper
Slope	-0.1948493	0.3986959	-0.49	0.642	-1.170423	0.7807243
Bias	1.62961	2.109311	0.77	0.469	-3.531689	6.790909

Supplementary Figure S4. Funnel plot and Egger test of imputed trials in multivariate analysis of *EGFR* mutation in early-stage LUAD

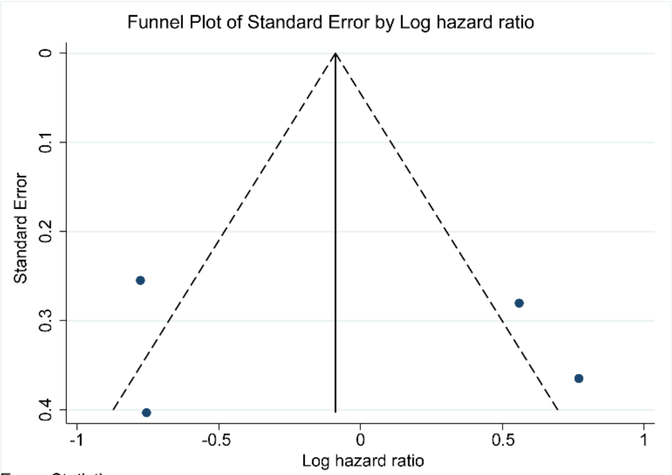

Egger Statistic

					95%CI	
Effect	Coefficient	Std.Err	t-statistic	P-value	Lower	Upper
Slope	-0.8032255	2.69521	-0.3	0.794	-12.39978	10.79333
Bias	2.349922	8.71353	0.27	0.813	-35.14137	39.84121

Supplementary Figure S5. Funnel plot and Egger test of imputed trials in univariate analysis of *EGFR* mutation in early-stage LUAD with pStage 0, I/II

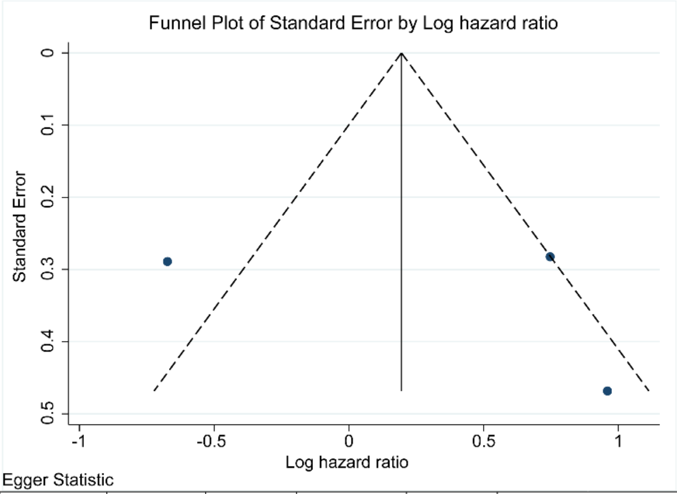

Egger Statistic

					95%CI	
Effect	Coefficient	Std.Err	t-statistic	P-value	Lower	Upper
Slope	-1.221209	3.216356	-0.38	0.769	-42.08889	39.64647
Bias	4.507462	10.01453	0.45	0.731	-122.7392	131.7542

Supplementary Figure S6. Funnel plot and Egger test of imputed trials in multivariate analysis of *EGFR* mutation in early-stage LUAD with pStage 0, I/II

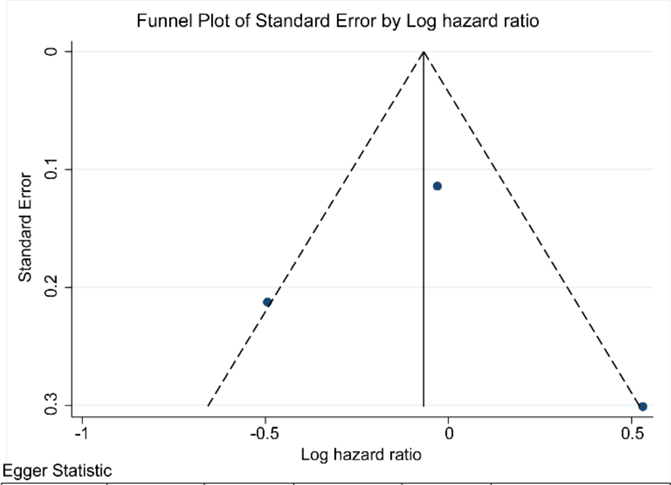

Egger Statistic

					95%CI	
Effect	Coefficient	Std.Err	t-statistic	P-value	Lower	Upper
Slope	-0.1740144	0.701559	-0.25	0.845	-9.088169	8.74014
Bias	0.6969788	4.247741	0.16	0.896	-53.27569	54.66965

Supplementary Figure S7. Funnel plot and Egger test of imputed trials in univariate analysis of alterations in exon 19 deletion and L858R

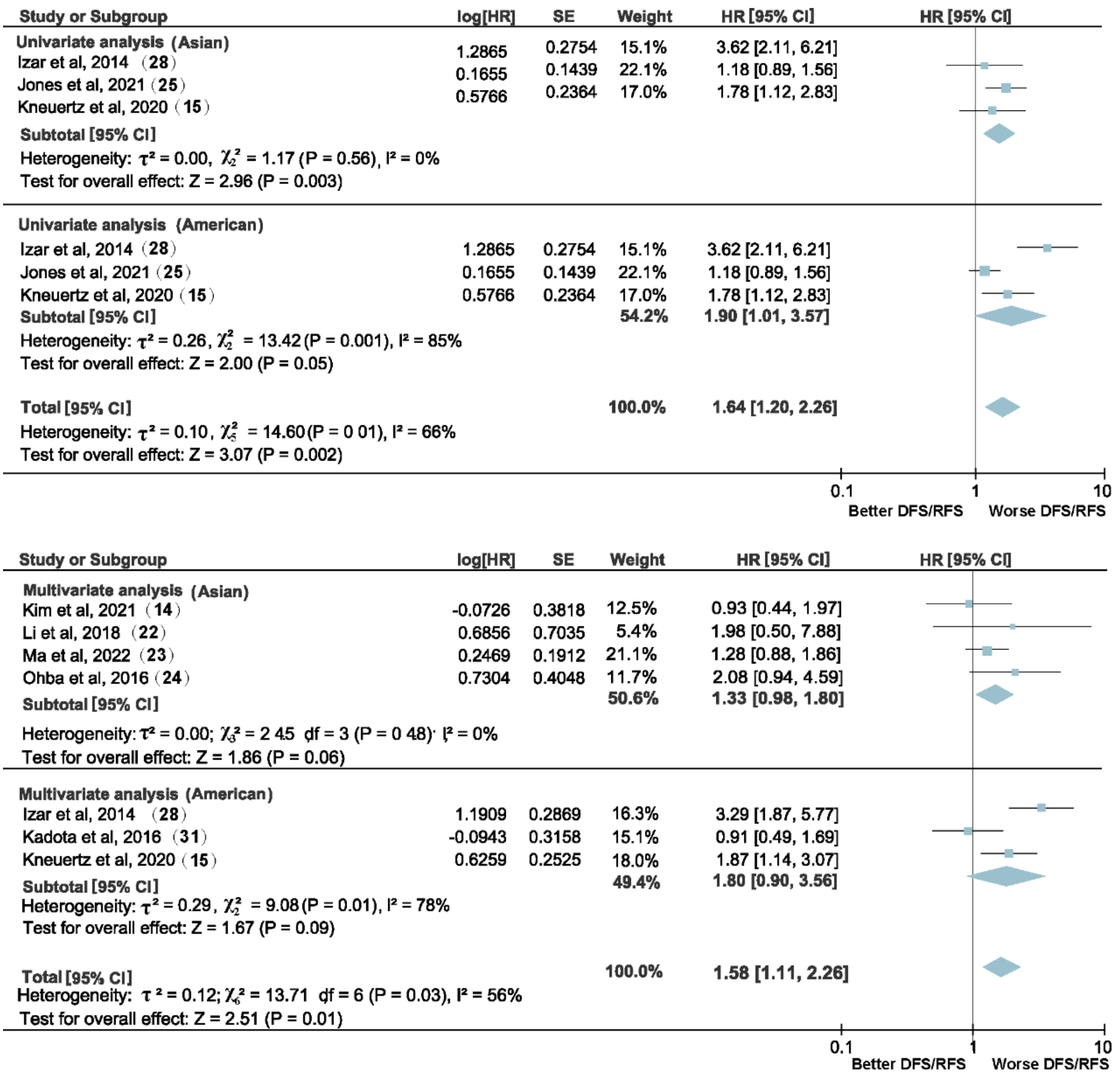

Supplementary Figure S8. Subgroup analysis of random-effects model for the association between *KRAS* mutation status and DFS/RFS in race. Upper section: Univariate analysis; Lower section: Multivariate analysis.

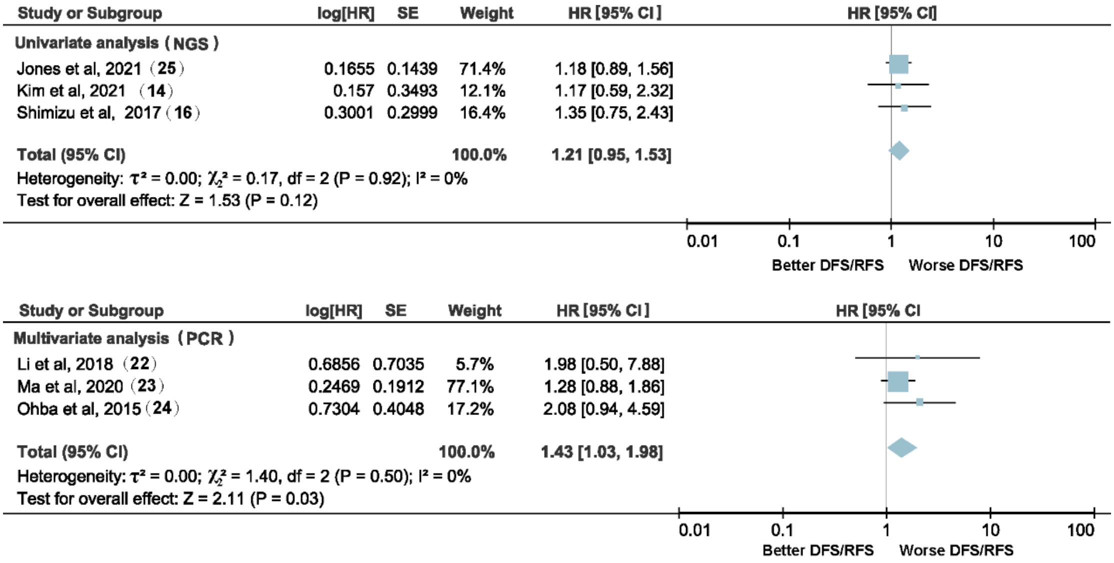

Supplementary Figure S9. Subgroup analysis of random-effects model for the association between *KRAS* mutation status and DFS/RFS in NGS and PCR platform. Upper section: Univariate analysis in NGS platform; Lower section: Multivariate analysis in PCR platform.

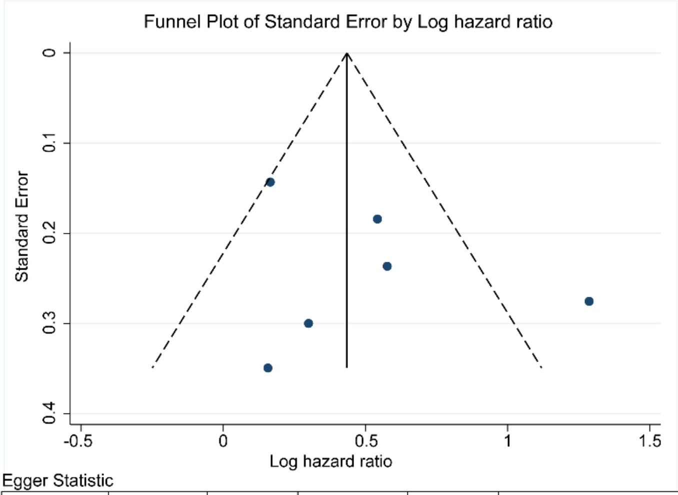

Egger Statistic

					95%CI	
Effect	Coefficient	Std.Err	t-statistic	P-value	Lower	Upper
Slope	0.0087169	0.508467	0.02	0.987	-1.403014	1.420448
Bias	2.071766	2.35456	0.88	0.429	-4.465542	8.609073

Supplementary Figure S10. Funnel plot and Egger test of imputed trials in univariate analysis of *KRAS* mutation in early-stage LUAD

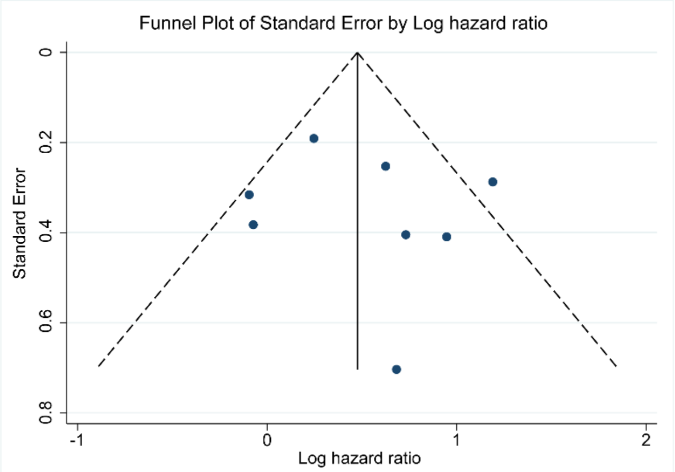

Egger Statistic

					95%CI	
Effect	Coefficient	Std.Err	t-statistic	P-value	Lower	Upper
Slope	0.2416524	0.509582	0.47	0.652	-1.005249	1.488554
Bias	0.8163734	1.672347	0.49	0.643	-3.275714	4.90846

Supplementary Figure S11. Funnel plot and Egger test of imputed trials in multivariate analysis of *KRAS* mutation in early-stage LUAD

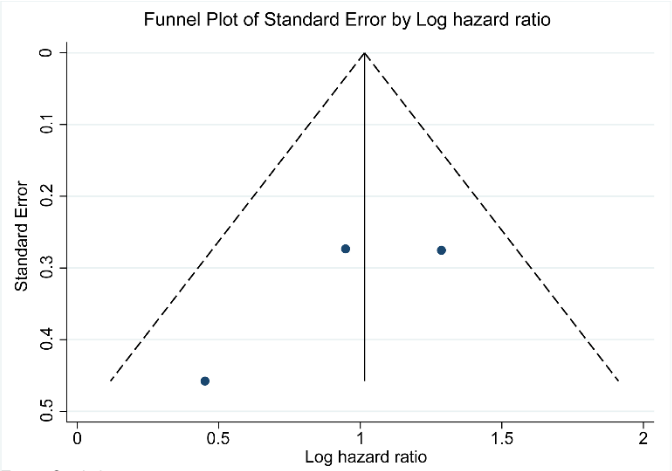

Egger Statistic

					95%CI	
Effect	Coefficient	Std.Err	t-statistic	P-value	Lower	Upper
Slope	2.100778	0.747482	2.81	0.218	-7.396887	11.59844
Bias	-3.592707	2.415848	-1.49	0.377	-34.28897	27.10356

Supplementary Figure S12. Funnel plot and Egger test of imputed trials in univariate analysis of *KRAS* mutation in early-stage LUAD with pStage I

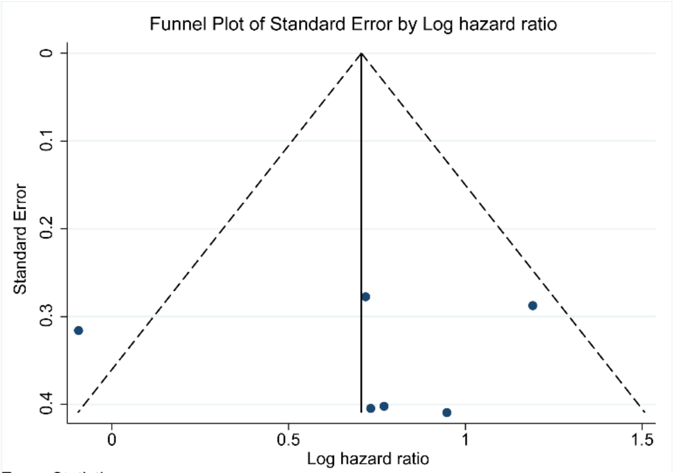

Egger Statistic

					95%CI	
Effect	Coefficient	Std.Err	t-statistic	P-value	Lower	Upper
Slope	0.6843746	1.287469	0.53	0.623	-2.890214	4.258963
Bias	0.0654809	3.840604	0.02	0.987	-10.59775	10.72871

Supplementary Figure S13. Funnel plot and Egger test of imputed trials in multivariate analysis of *KRAS* mutation in early-stage LUAD with pStage I
